# Safety, Pharmacokinetic, and Functional Effects of the Nogo-A Monoclonal Antibody in Amyotrophic Lateral Sclerosis: A Randomized, First-In-Human Clinical Trial

**DOI:** 10.1371/journal.pone.0097803

**Published:** 2014-05-19

**Authors:** Vincent Meininger, Pierre-François Pradat, Andrea Corse, Safa Al-Sarraj, Benjamin Rix Brooks, James B. Caress, Merit Cudkowicz, Stephen J. Kolb, Dale Lange, P. Nigel Leigh, Thomas Meyer, Stefano Milleri, Karen E. Morrison, Richard W. Orrell, Gary Peters, Jeffrey D. Rothstein, Jeremy Shefner, Arseniy Lavrov, Nicola Williams, Phil Overend, Jeffrey Price, Stewart Bates, Jonathan Bullman, David Krull, Alienor Berges, Bams Abila, Guy Meno-Tetang, Jens Wurthner

**Affiliations:** 1 Département des Maladies du Système Nerveux, Assistance Publique – Hôpitaux de Paris, Centre de Référence Maladies Rares SLA, Groupe Hospitalier Pitié-Salpêtrière, Université Pierre-et-Marie-Curie, Paris, France; 2 Neuromuscular Pathology Lab, Johns Hopkins University School of Medicine, Baltimore, Maryland, United States of America; 3 Department of Clinical Neuropathology, Kings College Hospital/Kings College London, London, United Kingdom; 4 Carolinas Neuromuscular/Amyotrophic Lateral Sclerosis-Muscular Dystrophy Association Center, Department of Neurology, Carolinas Medical Center and University of North Carolina School of Medicine-Charlotte Campus, Charlotte, North Carolina, United States of America; 5 Wake Forest School of Medicine, M Reynolds Tower, Medical Center Boulevard, Winston-Salem, North Carolina, United States of America; 6 Department of Neurology, Massachusetts General Hospital, Harvard Medical School, Boston, Massachusetts, United States of America; 7 Department of Neurology, The Ohio State University Wexner Medical Center, Columbus, Ohio, United States of America; 8 Department of Neurology, Weill Cornell School of Medicine, New York, New York, United States of America; 9 Trafford Centre for Biomedical Research, Brighton and Sussex Medical School, Falmer, Sussex, United Kingdom; 10 Department of Neurology, Charité – Universitätsmedizin Berlin, Berlin, Germany; 11 Centro Ricerche Cliniche, University Hospital G.B. Rossi, Verona, Italy; 12 School of Clinical and Experimental Medicine, University of Birmingham and Neurosciences Department, Queen Elizabeth Hospital, Birmingham, United Kingdom; 13 Department of Clinical Neuroscience, Institute of Neurology, University College London, London, United Kingdom; 14 GlaxoSmithKline Clinical Unit Cambridge, Addenbrooke's Hospital, Cambridge, United Kingdom; 15 Brain Science Institute, Johns Hopkins University, Department of Neurology, Baltimore, Maryland, United States of America; 16 Department of Neurology, SUNY Upstate Medical University, Syracuse, New York, United States of America; 17 Neurosciences Therapy Area Unit, Medicines Discovery and Development, GlaxoSmithKline, Stockley Park, Uxbridge, Middlesex, United Kingdom; 18 Clinical Statistics, GlaxoSmithKline, Stevenage, Hertfordshire, United Kingdom; 19 Clinical Pharmacology, Science and Study Operations, BioPharm and Infectious Diseases, GlaxoSmithKline, Stevenage, Hertfordshire, United Kingdom; 20 BioPharm Translational Medicine, GlaxoSmithKline, Stevenage, Hertfordshire, United Kingdom; 21 Clinical Pharmacology Modelling & Simulation (Neurosciences), GlaxoSmithKline, Stevenage, Hertfordshire, United Kingdom; 22 Safety Assessment, GlaxoSmithKline, Research Triangle Park, North Carolina, United States of America; 23 Clinical Pharmacology Modelling & Simulation, GlaxoSmithKline, Stockley Park, Uxbridge, Middlesex, United Kingdom; 24 Oncology Translational Medicine, Novartis Basel, Switzerland; 25 Unité Mixte de Recherche-678, Institut National de la Santé et de la Recherche Médicale - Université Pierre-et-Marie-Curie, Groupe Hospitalier Pitié-Salpêtrière, Paris, France; 26 Department of Neurology, Royal Free London NHS Foundation Trust, London, United Kingdom; Immunology Frontier Research Center, Osaka University, Japan

## Abstract

The neurite outgrowth inhibitor, Nogo-A, has been shown to be overexpressed in skeletal muscle in amyotrophic lateral sclerosis (ALS); it is both a potential biomarker and therapeutic target. We performed a double-blind, two-part, dose-escalation study, in subjects with ALS, assessing safety, pharmacokinetics (PK) and functional effects of ozanezumab, a humanized monoclonal antibody against Nogo-A. In Part 1, 40 subjects were randomized (3∶1) to receive single dose intravenous ozanezumab (0.01, 0.1, 1, 5, or 15 mg/kg) or placebo. In Part 2, 36 subjects were randomized (3∶1) to receive two repeat doses of intravenous ozanezumab (0.5, 2.5, or 15 mg/kg) or placebo, approximately 4 weeks apart. The primary endpoints were safety and tolerability (adverse events [AEs], vital signs, electrocardiogram (ECG), and clinical laboratory tests). Secondary endpoints included PK, immunogenicity, functional endpoints (clinical and electrophysiological), and biomarker parameters. Overall, ozanezumab treatment (0.01–15 mg/kg) was well tolerated. The overall incidence of AEs in the repeat dose 2.5 mg/kg and 15 mg/kg ozanezumab groups was higher than in the repeat dose placebo group and repeat dose 0.5 mg/kg ozanezumab group. The majority were considered not related to study drug by the investigators. Six serious AEs were reported in three subjects receiving ozanezumab; none were considered related to study drug. No study drug-related patterns were identified for ECG, laboratory, or vital signs parameters. One subject (repeat dose 15 mg/kg ozanezumab) showed a weak, positive anti-ozanezumab-antibody result. PK results were generally consistent with monoclonal antibody treatments. No apparent treatment effects were observed for functional endpoints or muscle biomarkers. Immunohistochemical staining showed dose-dependent co-localization of ozanezumab with Nogo-A in skeletal muscle. In conclusion, single and repeat dose ozanezumab treatment was well tolerated and demonstrated co-localization at the site of action. These findings support future studies with ozanezumab in ALS.

**Trial Registration:**

ClinicalTrials.gov NCT00875446 GSK-ClinicalStudyRegister.com GSK ID 111330

## Introduction

Amyotrophic lateral sclerosis (ALS) is characterized by selective and progressive loss of upper motor neurons of the motor cortex and lower motor neurons of the brainstem and spinal cord.[1]–[3] The main manifestations of ALS are progressive widespread muscle weakness and atrophy, leading to severe motor disability that affects speech, swallowing, respiratory function, and the extremities.[4] Cognitive impairment, predominantly in the form of executive dysfunction, may be detected in around 50% of patients, with up to 15% experiencing frontotemporal dementia.[5] Most patients die within 5 years of onset.[1], [4]

Excitotoxicity, i.e. an excessive drive of glutamate, is considered to be one of the mechanisms of neurodegeneration in ALS.[6] Riluzole, the only currently approved drug that alters survival in ALS, is thought to reduce excessive glutamatergic drive on neurons.[3], [7] Although the exact mechanism of action of riluzole is unclear, it is likely to involve several components, including inhibition of glutamate release, blockade of calcium and sodium channels, modulation of γ-Aminobutyric acid (GABA) transmission, as well as effects on N-Methyl-D-aspartate (NMDA) or α-Amino-3-hydroxy-5-methyl-4-isoxazolepropionic acid (AMPA) receptors.[7]–[9]

Nogo-A, a negative regulator of neuronal growth, is a potent neurite outgrowth inhibitor in the adult central nervous system and is expressed by oligodendrocytes.[10], [11] Outside the central nervous system Nogo-A is overexpressed in the skeletal muscle of the superoxide dismutase 1 (SOD1) transgenic mouse model of ALS, as well as in human skeletal muscle, as demonstrated in biopsies taken from patients with ALS.[12] Nogo-A expression in skeletal muscle has been proposed as an early diagnostic biomarker of ALS, with the level of expression reported to correlate with disease severity.[12]–[14] This view is challenged by reports suggesting that Nogo-A is a marker of muscle denervation rather than ALS specifically, noted to be up-regulated in muscle in preclinical denervation models and in muscle biopsies from subjects with a range of myopathies and peripheral neuropathies.[15]–[18] In the SOD1 transgenic mouse genetic ablation of Nogo-A prolonged survival and reduced muscle denervation,[19] while overexpression of Nogo-A in muscle fibers of mice induced neuromuscular junction instability and promoted denervation.[19] There is therefore a strong rationale for testing antibodies against Nogo-A in ALS. It is anticipated that blockade of Nogo-A may inhibit neurite retraction and potentially slow the axonal degeneration pattern in lower motor neurons that begins at the neuromuscular junction.[20] This may enhance motor neurone-muscle coupling, leading to functional improvement and survival benefits in patients with ALS.

Ozanezumab (GSK1223249: GlaxoSmithKline) is a humanized monoclonal antibody against Nogo-A, which is currently being investigated for the treatment of ALS. Ozanezumab has two possible modes of action: preventing binding of Nogo-A to the Nogo-A receptor and/or Nogo-A down-regulation by antibody-induced internalization of cell surface Nogo-A.[21]

Given that the anticipated mechanism of action of ozanezumab is via Nogo-A, which is not appreciably expressed in skeletal muscle under physiological conditions but is overexpressed in ALS, it was felt that conduct of a study in healthy subjects would not adequately reveal the potential risks or effects of treatment. Therefore, the first-in-human, Phase I/IIa study presented here was performed in subjects with ALS to assess the safety, pharmacokinetic (PK), and functional and biomarker effects of ozanezumab.

## Methods

### Study design

This was a randomized, placebo-controlled, double-blind, single and repeat dose-escalation, two-part study in subjects with ALS, conducted at 11 sites in France, Italy, the UK, and the USA, between May 2009 and September 2011. Screening took place within 28 days of the first dose of investigational product. In Part 1, escalating single doses (SD) of ozanezumab (0.01, 0.1, 1, 5, or 15 mg/kg administered intravenously [IV]), were evaluated in five sequential subject cohorts (8 subjects per cohort, randomized 3∶1 to receive ozanezumab or placebo). Part 2 was also of a sequential dose-escalating design: 36 subjects across three cohorts (12 subjects per cohort) were randomized (3∶1) to receive two repeat doses (RD) of ozanezumab (0.5, 2.5, or 15 mg/kg administered IV) or placebo, approximately 4 weeks apart. IV infusions were given over 60 minutes except for the 0.01 mg/kg dose, which was given over 11.2 minutes. Key safety data were reviewed by a blinded Dose Escalation Committee (comprising GlaxoSmithKline staff and an external expert neurologist who was experienced in ALS) before proceeding to the next dosing cohort. To ensure tolerability before proceeding, the first four subjects in all cohorts of Part 1 and the first cohort of Part 2 received treatment on consecutive days, so that only one subject was randomized and dosed within any 24-hour period. Dosing of all other subjects was not staggered. The follow-up period was at least 12 weeks for all subjects. Subjects in Part 2 were followed-up for 16 weeks and subjects receiving 15 mg/kg ozanezumab were followed-up for immunogenicity for 16–20 weeks.

### Ethics statement

The study protocol, protocol amendments, and informed consent were approved by a national, regional or investigational center ethics committee or an institutional review board (IRB), at each of the participating sites: Comité de Protection des Personnes Ile-de-France VI, Hôpital La Pitié-Salpêtrière, Paris, France; Comitato Etico per la Sperimentazione, Azienda Ospedaliera, Universitaria Integrata di Verona, Italy; Guy's Research Ethics Committee, St. Thomas Hospital, London, UK; Carolinas Healthcare System IRB, North Carolina; Wake Forest University Health Sciences, IRB, North Carolina; Johns Hopkins Medicine IRBs, Maryland; Western IRB, Washington; IRB, Weill Cornell Medical Center, New York; and IRB for the Protection of Human Subjects, SUNY Upstate Medical University, New York, USA. This study was conducted in accordance with Good Clinical Practice and the guiding principles of the Declaration of Helsinki, and all subjects provided written informed consent. This study is registered at clinicaltrials.gov/(NCT00875446) and at http://www.gsk-clinicalstudyregister.com (GSK ID 111330). The protocol for this trial and supporting CONSORT checklist are available as supporting information; see Checklist S1 and Protocol S1.

### Randomization and masking

Subjects in each cohort were centrally randomized across all sites via an Interactive Voice Response System. The randomization schedule was computer-generated using the validated in-house RandAll system. Infusions were prepared by a non-blinded pharmacist at the study site and infusion lines were masked in order to maintain the study blind.

### Patients

Eligible subjects were male or female of non-childbearing potential, 18–80 years of age, with a diagnosis of possible, laboratory-supported probable, probable or definite familial or sporadic ALS according to The Revised El Escorial diagnostic criteria,[22] and onset of muscle weakness within 60 months of study entry. Each subject was only allowed to participate in one part of the study. Subjects were also required to have a slow inspiratory vital capacity (SVC) ≥70% of predicted (changed by protocol amendment to include those with SVC <70% at the discretion of the investigator, as long as they did not show respiratory insufficiency). Medications (including riluzole) were required to have been stable within 28 days prior to dosing. Main exclusion criteria were: neuromuscular disorders (in addition to ALS, that could have impacted the study outcomes), dementia or psychiatric illnesses, that may have affected either outcome measures or patient understanding and/or compliance with the study requirements and procedures; positive alcohol or drugs tests at screening or a history of excessive alcohol consumption; vaccination within 3 weeks of study drug administration (originally 2 months; changed by protocol amendment); exposure to a clinical trial product within 6 months or exposure to four investigational products within 12 months of study drug administration; for patients undergoing muscle biopsies, wasted deltoids (Medical Research Council [MRC] score ≤2), and normal deltoids (MRC score 5) or those at a higher risk of bleeding complications.

### Endpoints and assessments

The primary endpoint was the safety and tolerability of SD or RD ozanezumab in subjects with ALS. Secondary endpoints included PK, immunogenicity, functional (clinical and electrophysiological) and biomarker analyses. Assessment timings are provided in Tables S1 and S2.

#### Safety

Adverse events (AEs), serious AEs (SAEs), electrocardiography (ECG), vital signs, and clinical laboratory tests (hematology and biochemistry) were monitored and assessed. ECG data for all subjects were centrally analyzed and reviewed by an independent cardiologist. Evaluation of safety signals also included any adverse effects on functional endpoints (clinical and electrophysiological) or immunogenicity.

#### Pharmacokinetics

Evaluation of plasma ozanezumab PK was performed at various time points in both parts of the study (Tables S1 and S2). PK parameters included: maximum observed plasma concentration (C_max_); area under the plasma concentration-time curve up to Week 4 and infinity (AUC_0–Week 4_ and AUC_0–∞_, respectively); terminal phase half-life; and clearance.

An assessment of ozanezumab concentrations was performed on skeletal muscle biopsies from subjects in Cohorts 3 and 5 from Part 1 and from all cohorts in Part 2.

#### Immunogenicity

Immunogenicity was assessed at various time points (Tables S1 and S2) from serum samples using an immune-electrochemiluminescent assay.

#### Functional endpoints

Functional endpoints (clinical and electrophysiological) were: ALS functional rating scale-revised (ALSFRS-R) score;[23]% predicted SVC;[24] manual muscle strength test (MMT);[25] and two motor unit number estimation (MUNE) endpoints (estimated number of motor units and mean single motor unit potential amplitude).[26]

#### Biomarkers

Exploratory biomarker analyses were performed on muscle biopsies, taken from the weaker deltoid muscle (grade 3 or 4 on the MRC scale) and plasma samples at pre- and post-dose. Ribonucleic acid (RNA) expression was assessed in muscle biopsy samples using quantitative reverse transcriptase polymerase chain reaction/whole genome microarray, while the expression of Nogo-A protein in muscle and plasma was analyzed using enzyme-linked chemiluminescence.

Frozen sections of muscle biopsies were examined by immunohistochemistry (IHC) and laser scanning cytometry (LSC). Expression of Nogo-A protein, ozanezumab, and gamma sarcoglycan was measured to quantify the co-localization of Nogo-A and ozanezumab within the muscle plasma membrane using LSC. Detailed methods are available in Methods S1 and Table S3.

#### Pharmacokinetic/functional endpoint relationship

Graphical exploration of a potential exposure–response relationship for ozanezumab was performed for ALSFRS-R score (monthly rate of decline) at each post-baseline visit, using average plasma ozanezumab concentration over the dosing interval as a measure of exposure.

### Statistical analyses

There was no formal calculation of power or sample size for this early phase clinical trial. The sample size was based on safety and feasibility. Safety data, drug concentration data, and PK parameters were presented in tabular and/or graphical format and summarized descriptively. All statistical analyses were performed in SAS software version 9.1.3 or higher. No formal hypotheses were tested. Point estimates and corresponding 95% confidence intervals were constructed for the difference between the mean of ozanezumab and the mean of placebo, calculated as µ(ozanezumab) - µ(placebo).

A mixed effects analysis of variance model was used to assess the dose proportionality of C_max_, AUC_0–Week 4_, and AUC_0–∞_, for SD and RD ozanezumab. Estimates of slope with respect to log (dose) together with 90% confidence intervals were used to quantify the degree of non-proportionality.

For ALSFRS-R and MMT, the slope (monthly rate of decline) was modelled using a random coefficients regression model. For % predicted SVC and both MUNE endpoints, the percentage change from screening was modeled using a mixed effects repeated measures model. Planned comparisons were made between each dose and placebo.

## Results

### Study population

**Figure 1:**
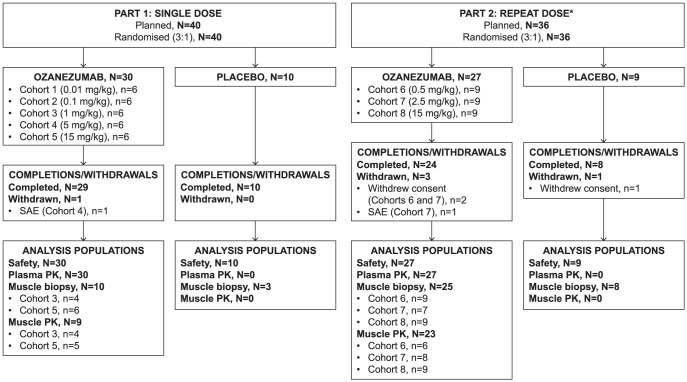
Subject disposition flow diagram for Parts 1 and 2. N, total number of subjects in group; n, number of subjects in category; PK, pharmacokinetics; SAE, serious adverse events. ^*^Two doses, received 4 weeks apart.

Of the 76 subjects who were enrolled, 71 completed the study (53 on ozanezumab, 18 on placebo). Subject disposition and baseline characteristics are presented in Figure 1. Across all subjects, the mean age was 58 years, mean body mass index was 26.2 kg/m^2^, and the majority of subjects were white males (Table 1). The majority of subjects had sporadic ALS (69/76; 91%) with a mean time from diagnosis of 11.1 months and a mean time from onset of muscle weakness of 19.4 months; the mean time from onset of muscle weakness varied considerably between these cohorts (Table 1). Fifty-nine (78%) subjects (35/40 and 24/36 in the SD and RD cohorts, respectively) were taking riluzole.

**Table 1:** Subject demographics and baseline characteristics.

Demographics	Placebo SD n = 10	Ozanezumab SD					Placebo RD n = 9	Ozanezumab RD*		
		0.01 mg/kg n = 6	0.1 mg/kg n = 6	1 mg/kg n = 6	5 mg/kg n = 6	15 mg/kg n = 6		0.5 mg/kg n = 9	2.5 mg/kg n = 9	15 mg/kg n = 9
Age in years, mean (STDV)	54.7 (11.38)	56.8 (9.47)	59.2 (6.82)	62.2 (6.15)	59.5 (11.64)	54.0 (17.58)	59.8 (11.60)	60.9 (8.16)	52.4 (10.99)	57.3 (7.86)
Sex (male), n (%)	7 (70)	4 (67)	5 (83)	6 (100)	6 (100)	3 (50)	6 (67)	7 (78)	7 (78)	6 (67)
BMI in kg/m^2^, mean (STDV)	24.69 (2.853)	27.70 (4.205)	25.90 (1.973)	25.81 (5.311)	27.13 (3.123)	26.80 (4.677)	27.24 (4.133)	26.03 (4.090)	26.47 (2.665)	25.27 (3.912)
Race; n (%)										
White	10 (100)	6 (100)	6 (100)	6 (100)	5 (83)†	6 (100)	9 (100)	9 (100)	9 (100)	9 (100)
Baseline characteristics										
Familial ALS, n (%)	1 (10)	1 (17)	1 (17)	1 (17)	0	0	0	1 (11)	2 (22)	0
Sporadic ALS, n (%)	9 (90)	5 (83)	5 (83)	5 (83)	6 (100)	6 (100)	9 (100)	8 (89)	7 (78)	9 (100)
Bulbar onset ALS, n (%)	0	1 (17)	1 (17)	0	1 (17)	2 (33)	2 (22)	1 (11)	0	3 (33)
Limb onset ALS, n (%)	10 (100)	5 (83)	5 (83)	6 (100)	5 (83)	4 (67)	6 (67)	7 (78)	9 (100)	6 (67)
Non bulbar/limb onset ALS, n (%)	0	0	0	0	0	0	1 (11)	1 (11)	0	0
Mean (STDV) time since onset of muscle weakness, months [min, max]	17.2 (14.37) [2, 29]	17.7 (7.28) [5], [18]	15.0 (3.95) [3], [9]	18.8 (7.31) [2], [24]	28.0 (23.90) [5, 60]	19.0 (7.29) [5], [17]	16.4 (9.81) [1, 34]	17.1(14.54)[0, 21]	31.4 (19.35) [1, 50]	14.2 (6.72) [2], [24]
Mean (STDV) time since ALS diagnosis, months	10.7 (7.85)	8.6 (4.85)	6.2 (2.64)	9.7 (9.69)	17.7 (21.07)	8.8 (4.41)	9.7 (9.81)	9.8 (7.40)	19.8 (16.53)	8.3 (6.83)
Mean (STDV) ALSFRS-R score	35.0 (5.60)	34.0 (6.13)	38.3 (4.08)	38.8 (5.42)	33.5 (3.99)	36.8 (5.08)	38.0 (5.66)	39.9 (6.15)	35.3 (6.98)	37.9 (3.41)

*Two doses, received 4 weeks apart.

† Remaining patient was of African American/African heritage. ALS, amyotrophic lateral sclerosis; ALSFRS-R, ALS functional rating scale-revised; BMI, body mass index; n, number of subjects; STDV, standard deviation; SD, single dose; RD, repeated dose.

### Safety

Overall, ozanezumab was well tolerated. Forty-seven (62%) subjects across all cohorts (35/57 [61%] on active treatment, 12/19 [63%] on placebo) reported at least one AE (Table 2). The proportion of subjects who reported ≥1 AE in the ozanezumab groups was similar when compared with the placebo group (61% and 63%, respectively) although the overall incidence of AEs in the RD 2.5 mg/kg and 15 mg/kg ozanezumab groups (78% and 89% of subjects with any AE, respectively) was higher than in the RD placebo group (56%) and the RD 0.5 mg/kg ozanezumab group (44%). Most AEs were of mild or moderate intensity as judged by the investigator. Seven (9%) subjects (5/57 [8.8%] on active treatment, 2/19 [10.5%] on placebo) reported eight AEs of severe intensity. Among the severe AEs, three subjects experienced severe headache. Two of these were in placebo groups. The third subject reported onset of headache 9 days after receiving the first dose of ozanezumab (RD 2.5 mg/kg group); this was resolved after 6 days and was not considered related to treatment. Another subject experienced severe dysphagia (RD 15 mg/kg ozanezumab), which was unresolved at the end of the study and considered not related to study drug. The remaining four severe AEs in three subjects were classed as SAEs.

**Table 2:** Summary of adverse events in Part 1 (SD) and Part 2 (RD).

Preferred term	Placebo SD	Ozanezumab SD					Placebo RD	Ozanezumab RD† **		
	n = 10	0.01 mg/kg	0.1 mg/kg	1 mg/kg	5 mg/kg	15 mg/kg	n = 9	0.5 mg/kg	2.5 mg/kg	15 mg/kg
		n = 6	n = 6	n = 6	n = 6	n = 6		n = 9	n = 9	n = 9
Subjects with any AE, n (%)*	7 (70)	2 (33)	3 (50)	3 (50)	4 (67)	4 (67)	5 (56)	4 (44)	7 (78)	8 (89)
Back pain	0	0	0	0	1 (17)	0	2 (22)	0	0	1 (11)
Bronchitis	2 (20)	0	1 (17)	0	0	0	0	1 (11)	0	0
Fall	2 (20)	1 (17)	1 (17)	2 (33)	0	0	1 (11)	0	0	3 (33)
Headache	1 (10)	0	0	0	1 (17)	2 (33)	1 (11)	0	2 (22)	1 (11)
Procedural pain	0	0	0	0	0	0	1 (11)	0	2 (22)	2 (22)
Subjects with mild AEs	5 (50)	1 (17)	0	2 (33)	1 (17)	2 (33)	2 (22)	2 (22)	4 (44)	4 (44)
Subjects with moderate AEs	1 (10)	1 (17)	3 (50)	1 (17)	2 (33)	1 (17)	2 (22)	2 (22)	1 (11)	3 (33)
Subjects with severe AEs	1 (10)	0	0	0	1 (17)	1 (17)	1 (11)	0	2 (22)	1 (11)
Subjects with any drug-related AE, n (%)‡	1 (10)	1 (17)	0	0	0	0	1 (11)	0	3 (33)	2 (22)
Number of drug-related AEs	2	2	0	0	0	0	4	0	9	15
Asthenia	0	1 (17)	0	0	0	0	0	0	1 (11)	1 (11)
Headache	0	0	0	0	0	0	1 (11)	0	1 (11)	1 (11)
Paraesthesia	1 (10)	1 (17)	0	0	0	0	0	0	0	0
Procedural pain	0	0	0	0	0	0	0	0	1 (11)	1 (11)
Subjects with serious AE, n (%)	0	0	0	0	1 (17)	1 (17)	0	0	1 (11)	0
Number of serious AEs	0	0	0	0	2	1	0	0	3	0

*Only those occurring in ≥4 subjects across all cohorts are listed.

† Five unreported AEs were identified (in 3 subjects): 1 subject experienced severe diarrhea (2.5 mg/kg), 1 subject experienced shoulder pain, secondary to muscle biopsy (15 mg/kg), and 1 subject experienced some minor skin bruising, a fall and a hard swelling on the left hip (resulting from the fall) (15 mg/kg).

**Two doses, received 4 weeks apart.

‡ Only those occurring in ≥2 subjects across all cohorts are listed. AE, adverse event; n, number of subjects; SD, single dose; RD, repeated dose.

Thirty-two AEs (29 mild, one moderate, one severe, and one of unknown severity), that were considered possibly related to the study medication, were reported by eight subjects (Table 2). None resulted in withdrawal from the study. Most common AEs (reported in ≥4 subjects across all cohorts) were back pain, bronchitis, fall, headache and procedural pain at biopsy site (Table 2). AEs in the cardiac disorders category were reported in four subjects. One of these, sinus tachycardia of mild intensity, was considered possibly related to investigational product (RD 2.5 mg/kg ozanezumab). Other events, not considered related to study drug, included ventricular extrasystoles (SD 5 mg/kg ozanezumab), which resolved after 2 hours, with the subject remaining asymptomatic with no findings of clinical concern on Holter monitoring; second degree atrioventricular block (RD 2.5 mg/kg ozanezumab); and cardiac arrest and cardio-respiratory arrest, as described under SAEs below.

Six SAEs were reported in three subjects receiving ozanezumab, all considered by the investigators as unrelated to study medication. One subject (SD 15 mg/kg ozanezumab) suffered a head injury after an accidental fall, which led to hospitalization and ultimately resolved. The second subject (SD 5 mg/kg ozanezumab) experienced excess bronchial secretions resulting in hospitalization, and later died from respiratory failure, 17 weeks after the dosing. The third subject (RD 2.5 mg/kg ozanezumab) was hospitalized for abdominal pain, for which the etiology remained elusive; this subject later died from cardiac arrest and cardio-respiratory arrest 10.5 weeks after the second dose.

The events common to ALS showed no patterns of reporting to suggest an adverse drug effect on the underlying condition. The most frequently reported event common to ALS was weakness, occurring in 8/19 (42%) and 19/57 (33%) subjects in the placebo and ozanezumab groups, respectively.

Analysis of ECG data showed that the proportion of subjects with QTcB or QTcF values in the lowest category of potential clinical importance (absolute QTc >450 msec and up to 480 msec or QTc change from baseline >30 msec and up to 60 msec) was slightly greater in the ozanezumab RD 15 mg/kg dose group compared with the other RD groups (Table S4). An absolute QTc value >500 msec (or >480 msec) or a QTc change from baseline >60 msec was not observed in any subject at any point. Evaluation of these data demonstrated no clear patterns indicative of a treatment effect on ECG parameters.

Vital signs showed no notable differences between ozanezumab groups and placebo. No clinically significant safety laboratory findings were reported by the investigator. However, there were findings of potential clinical importance (PCI). Three subjects experienced elevations in liver function tests; one subject (SD placebo) had an alanine aminotransferase (ALT) value approximately 2× the upper limit of normal (ULN), elevated aspartate aminotransferase (aspartate aminotransferase [AST]; <2× ULN) and elevated gamma-glutamyl transpeptidase (GGT; approximately 3× ULN), on Day 22. A second subject (SD 5 mg/kg ozanezumab) had elevated total bilirubin (approximately 2× ULN) on Day 50, and a third subject (RD 2.5 mg/kg ozanezumab) had an elevated ALT value (approximately 3× ULN), accompanied by elevations of AST (<2× ULN) and GGT (approximately 3× ULN). Seven subjects had elevated glucose levels of PCI, all of whom received ozanezumab; these elevations occurred at least 1 week after dosing in five of these subjects. Isolated cases of hyperglycemia were seen in patients who received ozanezumab. The findings were based on random blood glucose samples. None of the elevated glucose values were considered clinically significant by the investigator and were not reported as AEs. There was no apparent dose-response. Mild hematological abnormalities occurred in four subjects receiving ozanezumab. However, these were not considered clinically significant. Elevated creatine kinase levels were observed in a number of subjects across all treatment groups but this is a common finding in patients with ALS and was not considered clinically relevant. Urinalysis findings raised no safety concerns. See Results S1 for additional details of laboratory findings.

Changes in heart rate were reported for a small number of subjects but none were considered clinically significant. Two subjects experienced changes in blood pressure that were recorded as AEs. However, neither event was considered related to the study drug.

### Pharmacokinetics

**Figure 2:**
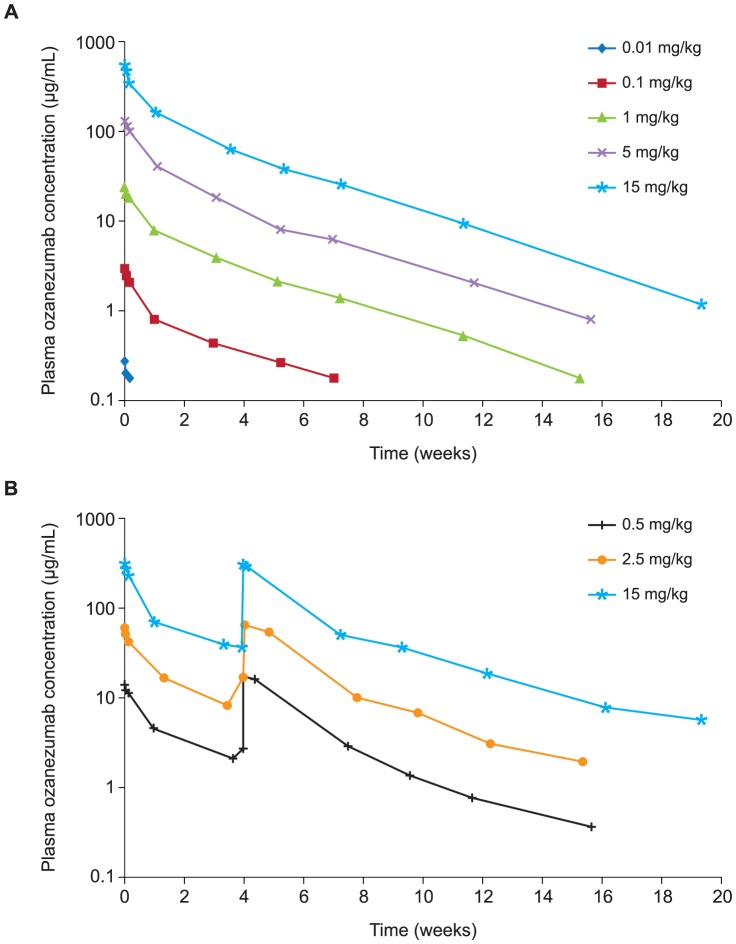
Mean plasma ozanezumab concentration-time profiles following single (A) or two (B) IV infusions of ozanezumab.

Peak plasma ozanezumab concentrations were generally observed at the end of infusion. C_max_ and AUC typically increased proportional to dose, while concentrations declined in a bi-exponential fashion, with a terminal elimination half-life of approximately 20 days (Table 3 and Figure 2).

**Table 3:** Ozanezumab pharmacokinetic parameters.

	Ozanezumab SD					Ozanezumab RD*					
	0.01 mg/kg n = 6	0.1 mg/kg n = 6	1 mg/kg n = 6	5 mg/kg n = 6	15 mg/kg n = 6	0.5 mg/kg n = 9		2.5 mg/kg n = 9		15 mg/kg n = 9	
						Dose 1	Dose 2	Dose 1	Dose 2	Dose 1	Dose 2
T_max_ (h)	1.00 (0.98–1.00)	1.125 (1.00–10.05)	1.06 (1.00–23.17)	1.05 (0.05–1.18)	1.08 (1.00–10.00)	1.02 (1.00–24.00)	1.08 (1.00–6.03)	1.02 (0.97–10.00)	6.00 (1.00–6.10)	1.00 (1.00–10.07)	1.10 (1.02–6.00)
C_max_ ( µg/mL)	0.265 (17.9)	2.93 (27.7)	23.2 (22.8)	125 (28.0)	527 (18.3)	13.8 (17.0)	16.9 (36.2)	58.7 (22.8)	65.9 (23.2)	280 (20.9)	311 (14.9)
AUC_0–Week 4_ ( µg.h/mL)	17.6 (15.4)	504 (27.2)	4647 (14.1)	23888 (28.0)	92776 (17.0)	2808 (20.9)	4063 (53.7)	10461 (36.4)	19133 (25.9)	46704 (18.5)	76337 (17.0)
AUC_0–∞_ ( µg.h/mL)	-	749 (25.5)	6723 (16.8)	32503 (31.7)	128309 (22.4)	-	5420 (44.7)	-	25020 (29.9)	-	109904 (18.8)
Clearance (mL/h)	-	10.0 (31.1)	11.7 (25.1)	12.7 (38.0)	9.19 (36.9)	-	7.21 (50.0)	-	7.80 (25.8)	-	9.69 (26.6)
t_1/2_ (days)	-	19.6 (0.6)	20.8 (0.8)	17.8 (0.8)	18.1 (0.8)	-	18.8 (1.3)	-	16.1 (1.7)	-	22.5 (0.9)

*Two doses, received 4 weeks apart. AUC_0–Week 4_, area under the plasma concentration-time curve up to Week 4; AUC_0–∞_, area under the plasma concentration-time curve up to infinity; C_max_, maximum observed plasma concentration; n, number of subjects; RD, repeated dose; SD, single dose; t_1/2_, apparent terminal phase half-life; T_max_, time at which C_max_ was observed. T_max_ presented as median (range); all other values presented as geometric mean of the log-transformed data (coefficient of variation, CV%).

### Immunogenicity

In one subject (RD 15 mg/kg ozanezumab) anti-ozanezumab antibodies were detected at Week 20 (weak titer of 2), having shown negative results at all previous time points. No AEs indicative of an immune-related reaction were reported for this subject around the time of the positive antibody response. No anti-ozanezumab antibodies were detected in any other subject.

### Functional endpoints (clinical and electrophysiological)

There were no apparent treatment effects on ALSFRS-R slope, % of predicted SVC percentage change from screening or MMT slope following treatment with ozanezumab compared with placebo. However, a numerical difference in favor of ozanezumab versus placebo was observed in the RD 15 mg/kg group for each endpoint (Tables S5 and S6).

In MUNE data analysis, no trends between ozanezumab groups and placebo were observed (Table S6).

### Biomarkers

There was no evidence of a pharmacological response with ozanezumab treatment on protein or RNA biomarkers.

**Figure 3:**
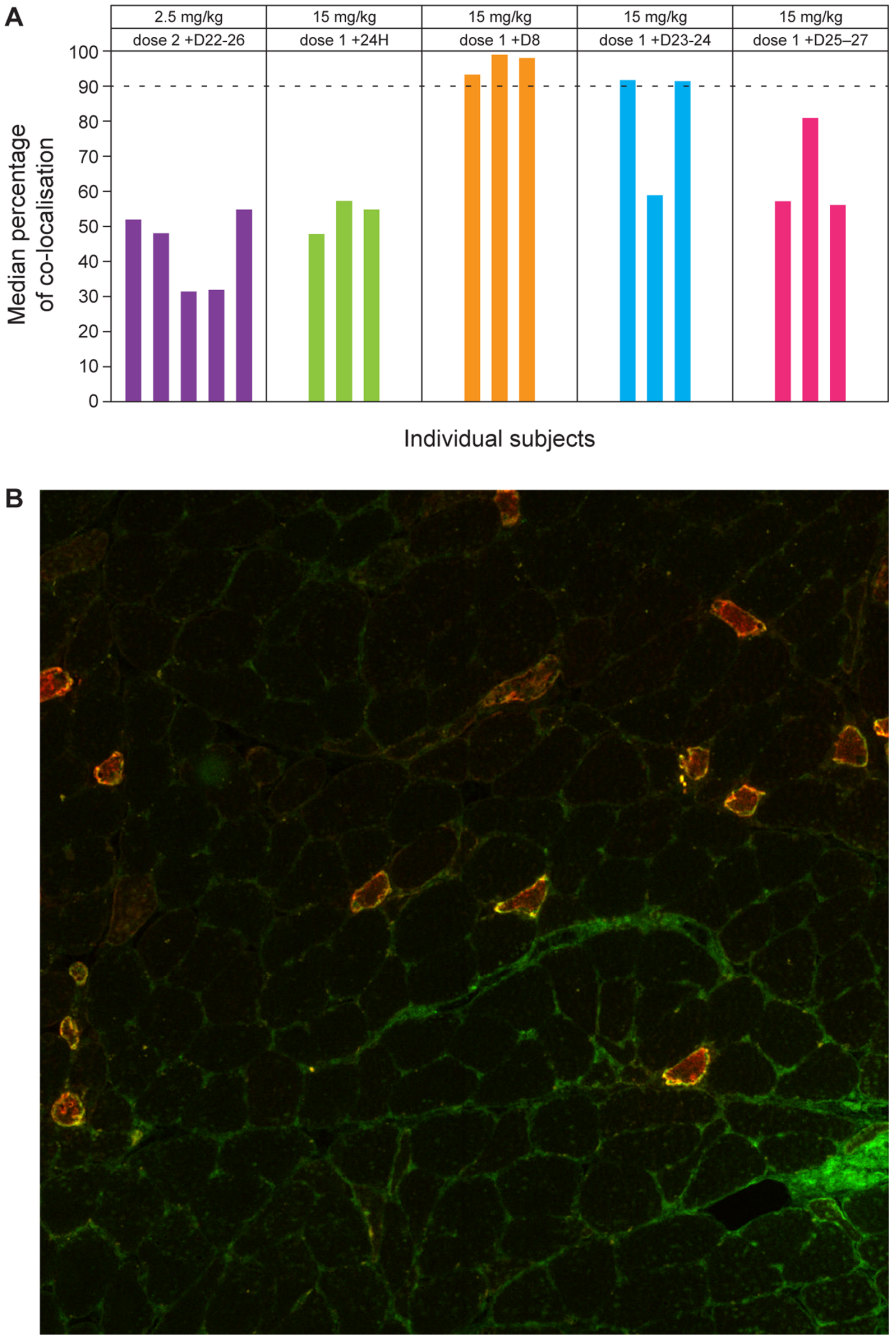
Co-localization of membrane Nogo-A with ozanezumab in skeletal muscle of individual subjects. A. Triplicate readings are provided from biopsies in Cohort 7 and 8 (single reading from Cohort 5) dose 2 +D22–26, biopsy taken 22–26 days after the second dose; dose 1 +24H, biopsy taken 24 hours after first dose. B. Nogo-A (red), ozanezumab (green) and co-localization (yellow), in muscle biopsy, 24 hours post-dose.

Measurement of Nogo-A and ozanezumab using IHC staining of muscle biopsies suggested co-localization of the drug at the site of action in skeletal muscle. Co-localization followed a similar trend to ozanezumab levels in muscle, suggesting that this was related to exposure. Greater than 90% co-localization was observed with the 15 mg/kg dose, 8 days after dosing, with levels dropping below 90% at 3–4 weeks post-dose (Figure 3).

### Pharmacokinetic/functional endpoint relationship

Following graphical exploration of a potential exposure-response relationship of ozanezumab for ALSFRS-R score (monthly rate of decline) at each post-baseline visit, no PK/functional endpoint relationship was identified.

## Discussion

The effort to develop new treatments for ALS has led to repeated failure since the demonstration that riluzole extended survival.[3] Despite initially encouraging results from a Phase II trial, which suggested beneficial effects on ALSFRS-R and survival,[27] the recent negative results of the dexpramipexole Phase III study is yet another disappointing example.[28] Thus, there is still an urgent need for new treatments for ALS. A number of compounds targeting different aspects of ALS pathogenesis are currently being investigated. This was the first study in humans of ozanezumab, a monoclonal antibody targeting Nogo-A.

Generally the baseline characteristics were similar between treatment groups, however, as the inclusion criteria permitted a history of muscle weakness of up to 60 months; 8/76 (11%) patients reported having muscle weakness for 36 months or longer. This could have caused a bias towards long-term survivors and since the patient cohorts were small, the mean disease duration varied considerably between these cohorts.

Ozanezumab was generally well tolerated at single doses of 0.01–15 mg/kg and two repeat doses of 0.5–15 mg/kg, with no adverse drug effects on the underlying condition.

Although there was no clear relationship between reported AEs and increasing doses of ozanezumab, the overall incidence of AEs was numerically higher in the two higher dose RD cohorts compared with placebo and the lowest dose RD cohort. Despite the higher incidence of AEs in the RD cohorts, proportions of mild, moderate and severe AEs were comparable. SAEs and deaths were not attributed to the study drug, though one cardiac non-serious AE (mild sinus tachycardia) was considered possibly related. There were no clinically significant safety laboratory findings and no clear patterns indicative of a treatment effect on ECG parameters or vital signs.

Following IV infusion, the plasma PK characteristics of ozanezumab were generally consistent with those of a humanized immunoglobulin G monoclonal antibody. One observation of note was the approximate 2-fold difference between SD and RD (first dose) ozanezumab 15 mg/kg in average C_max_ and AUC values. Examination of the individual data suggested no overlap in values between the two cohorts. However, detailed investigations of drug substance, subject demographics, individual dosing records, and the PK plasma sample assay methodology revealed no explanation for this difference. Consequently, the difference appeared to be genuine, and possibly attributed to between-cohort variability.

This study was not statistically powered to detect changes in functional (clinical and electrophysiological) endpoints. Despite this, and despite subjects receiving only one or two doses each, a trend was observed for clinical endpoints such as ALSFRS-R, SVC, and MMT, which possibly suggested a response in the highest dose cohorts. Trends observed should be interpreted with great caution given the small sample size, and other studies will be required to confirm and further investigate these trends. The relationship between ozanezumab administration and pharmacological signal on protein or RNA requires further investigation; the current study may have been too short to detect such responses. In future studies, optimum time points, treatment times, and analysis methods will need to be established.

IHC staining of muscle biopsies suggested that there were dose-dependent changes in ozanezumab quantification and detection and that ozanezumab distributed and co-localized with Nogo-A at the site of action in skeletal muscle. Following the full distribution of ozanezumab to muscle tissue and peak co-localization approximately 1 week after dosing, the percentage of co-localisation appeared to be related to levels of ozanezumab, suggesting a possible relationship with exposure.

Overall, ozanezumab was well tolerated and PK parameters were generally consistent with those of humanized monoclonal antibodies. The trends observed on functional endpoints in the present study, along with the co-localization of ozanezumab in skeletal muscle, are encouraging; these observations, along with the lack of emerging safety signals, support future studies of ozanezumab in this devastating disease, and a Phase II study of efficacy and safety of ozanezumab (NCT01753076) is currently underway.

## Supporting Information

Table S1 **Assessment schedules for Part 1.** AE, adverse event; ALSFRS-R, amyotrophic lateral sclerosis functional rating scale-revised; ECG, electrocardiogram; FU, follow-up; MUNE, motor unit number estimation; PK, pharmacokinetic; SAE, serious adverse event. ^*^The precise timing of safety, functional assessments, and PK blood sampling may have been altered during the course of the study based on emerging data. If the profile indicated that more sampling or assessments were needed, additional time points were to be added. Study assessments to follow PK sampling at end of the infusion. (The 1-hour PK sample was collected directly at the end of the infusion, Cohorts 2–8). ^†^Only SAEs related to study participation were collected prior to the start of the investigational product. Once the investigational product infusion began, all AEs and SAEs were collected until the last FU visit. ^‡^Continuous Lead II ECG commenced approximately 1 hour pre-dose on Day 1 until 24 hours post-dose. ^§^Muscle biopsies, which were voluntary collections in Cohort 3 (1 mg/kg) and required collections in Cohort 5 (15 mg/kg) were collected pre-dose and at Week 4. The pre-dose muscle biopsy was only to be done when the subject had passed all screening assessments and eligibility had been reconfirmed. This meant the pre-dose biopsy could be done at any appropriate time before Day 1. Blood samples at pre-dose and at Week 4 were collected regardless of whether or not a muscle biopsy was to be taken. The number and schedule of FU visits after Week 12 for each subject was to vary depending on plasma concentrations of ozanezumab reaching a low enough level to allow a final blood sample to have been assayed for immunogenicity.
(DOCX)

Table S2 **Assessment schedules for Part 2.** AE, adverse event; ALSFRS-R, amyotrophic lateral sclerosis functional rating scale-revised; ECG, electrocardiogram; FU, follow-up; MUNE, motor unit number estimation; PK, pharmacokinetic; SAE, serious adverse event. ^*^The precise timing of safety, functional assessments and PK blood sampling may have been altered during the course of the study based on emerging data. If the profile indicated that more sampling or assessments were needed, additional time points were to be added. Study assessments to follow PK sampling at end of infusion. (The 1-hour PK sample was collected directly at the end of the infusion, Cohorts 2–8). ^†^Only SAEs related to study participation were collected prior to the start of the investigational product. Once the investigational product infusion began, all AEs and SAEs were collected until the last FU visit. ^‡^Continuous Lead II ECG commenced approximately 1 hour pre-dose until 24 hours post-Dose 1 and for 6 hours post-Dose 2. ^§^In cohorts 6 and 7 the pre-dose muscle biopsy and blood sample were only done when the subject had passed all screening assessments and eligibility had been reconfirmed. This meant the pre-dose biopsy and blood sample could be done at any appropriate time before Day 1. The post-dose muscle biopsy and blood sample were scheduled for collection at Week 8 (unless emerging data suggested the post-dose muscle biopsy and blood sample should have been collected at an alternative week). In cohort 8, muscle biopsies and blood sample were collected from subjects at pre-dose and at one time point after the first dose. Subjects were assigned for a post-dose muscle biopsy and blood sample collection at either Day 1 (+24 hours), Day 8 or Week 4 (Day 22–24) based on subject preference determined at screening (see Section 7.3). If the subject had the Day 1 (+24 hours) collection then the pre-dose muscle biopsy and blood sample were collected at least 8 days before Day 1. ^#^The number and schedule of FU visits after Week 16 for each subject varied depending on plasma concentrations of ozanezumab reaching a low enough level to allow a final blood sample to have been taken for immunogenicity assays.
(DOCX)

Table S3 **Primer and probe sets used in biomarker analyses.**
(DOCX)

Table S4 **Summary of QTc values of potential clinical importance at any visit post-baseline.** n, number of subjects; SD, single dose; RD, repeated dose. ^*^Two doses, received 4 weeks apart.
(DOCX)

Table S5 **Summary of ALSFRS-R and MMT analyses.** n, number of evaluable subjects; RD, repeat dose; SD, single dose; SE, standard error. ALSFRS-R, ALS functional rating scale-revised; CI, confidence interval; MMT, manual muscle strength test; Measured at Week 12 for SD study, Week 16 for RD study. ^*^Two doses, received 4 weeks apart.
(DOCX)

Table S6 **Summary of SVC and MUNE analyses.** CI, confidence interval; MUNE, motor unit number estimation; n, number of evaluable subjects; RD, repeat dose; SD, single dose; SE, standard error; SMUP, single motor unit potential; SVC, slow inspiratory vital capacity. Measured at Week 12 for SD study, Week 16 for RD study. ^*^Two doses, received 4 weeks apart.
(DOCX)

Methods S1 (DOC)

Results S1 (DOCX)

Checklist S1 **CONSORT checklist.**
(DOC)

Protocol S1 **Redacted protocol.**
(PDF)
