# Supplementary material for: Safety, Pharmacokinetic, and Functional Effects of the Nogo-A Monoclonal Antibody in Amyotrophic Lateral Sclerosis: A Randomized, First-In-Human Clinical Trial
Source: PLoS One. 2014 May 19;9(5):e97803. doi: 10.1371/journal.pone.0097803 (PMC4026380; doi:10.1371/journal.pone.0097803)
Supplement: Table S1 — Assessment schedules for Part 1. AE, adverse event; ALSFRS-R, amyotrophic lateral sclerosis functional rating scale-revised; ECG, electrocardiogram; FU, follow-up; MUNE, motor unit number estimation; PK, pharmacokinetic; SAE, serious adverse event. *The precise timing of safety, functional assessments, and PK blood sampling may have been altered during the course of the study based on emerging data. If the profile indicated that more sampling or assessments were needed, additional time points were to be added. Study assessments to follow PK sampling at end of the infusion. (The 1-hour PK sample was collected directly at the end of the infusion, Cohorts 2–8). †Only SAEs related to study participation were collected prior to the start of the investigational product. Once the investigational product infusion began, all AEs and SAEs were collected until the last FU visit. ‡Continuous Lead II ECG commenced approximately 1 hour pre-dose on Day 1 until 24 hours post-dose. §Muscle biopsies, which were voluntary collections in Cohort 3 (1 mg/kg) and required collections in Cohort 5 (15 mg/kg) were collected pre-dose and at Week 4. The pre-dose muscle biopsy was only to be done when the subject had passed all screening assessments and eligibility had been reconfirmed. This meant the pre-dose biopsy could be done at any appropriate time before Day 1. Blood samples at pre-dose and at Week 4 were collected regardless of whether or not a muscle biopsy was to be taken. The number and schedule of FU visits after Week 12 for each subject was to vary depending on plasma concentrations of ozanezumab reaching a low enough level to allow a final blood sample to have been assayed for immunogenicity. (DOCX) [file pone.0097803.s001.docx]

## Table S1. Assessment schedules for Part 1.

| Procedure | Assessments^*^ |
| --- | --- |
| ALSFRS-R | Screening; Weeks 6 and 12 after dosing. |
| Manual muscle strength test | Screening; Weeks 6 and 12 after dosing. |
| Electrophysiology (MUNE) | Screening; Weeks 6 and 12 after dosing. |
| Slow inspiratory vital capacity | Screening; Weeks 6 and 12 after dosing. |
| Adverse events^†^ | Screening; pre-dose; continuously on Day 1; Weeks 2, 4, 6, 8, and 12 after dosing. |
| Clinical laboratory tests | Screening; pre-dose; 24 h after dosing; Weeks 2, 4, 6, 8, and 12 after dosing. |
| Vital signs (supine and standing) taken immediately prior to infusion | Screening; pre-dose; 1, 2, 3, 4, 6, 8, 12, and 24 hours after dosing; Weeks 2, 4, 6, 8, and 12 after dosing. |
| 12-Lead ECG (QT and other intervals) | Screening; pre-dose; 2, 4, 8, 12, and 24 hours after dosing; Weeks 2, 4, 6, 8, and 12 after dosing. |
| Continuous ECG monitoring (telemetry or bedside ECG monitor)^‡^ | Pre-dose until the end of Day 1. |
| Deltoid muscle biopsy^§^ | Pre-dose; and Week 4 after dosing. |
| Blood sample for biomarkers^§^ | Pre-dose; and Week 4 after dosing. |
| Blood sample for immunogenicity**^¶^** | Pre-dose; Weeks 2, 4, 8, and 12 after dosing; FU visit. |
| Blood sample for pharmacokinetics | Pre-dose; 1, 10, and 24 hours after dosing; Weeks 2, 4, 6, 8, and 12 after dosing; FU visit. |

AE, adverse event; ALSFRS-R, amyotrophic lateral sclerosis functional rating scale-revised; ECG, electrocardiogram; FU, follow-up; MUNE, motor unit number estimation; PK, pharmacokinetic; SAE, serious adverse event.

^*^The precise timing of safety, functional assessments, and PK blood sampling may have been altered during the course of the study based on emerging data. If the profile indicated that more sampling or assessments were needed, additional time points were to be added. Study assessments to follow PK sampling at end of the infusion. (The 1-hour PK sample was collected directly at the end of the infusion, Cohorts 2–8).

^†^Only SAEs related to study participation were collected prior to the start of the investigational product. Once the investigational product infusion began, all AEs and SAEs were collected until the last FU visit.

^‡^Continuous Lead II ECG commenced approximately 1 hour pre-dose on Day 1 until 24 hours post-dose.

^§^Muscle biopsies, which were voluntary collections in Cohort 3 (1 mg/kg) and required collections in Cohort 5 (15 mg/kg) were collected pre-dose and at Week 4. The pre-dose muscle biopsy was only to be done when the subject had passed all screening assessments and eligibility had been reconfirmed. This meant the pre-dose biopsy could be done at any appropriate time before Day 1. Blood samples at pre-dose and at Week 4 were collected regardless of whether or not a muscle biopsy was to be taken.

**^¶^**The number and schedule of FU visits after Week 12 for each subject was to vary depending on plasma concentrations of ozanezumab reaching a low enough level to allow a final blood sample to have been assayed for immunogenicity.
