# Supplementary material for: Safety, Pharmacokinetic, and Functional Effects of the Nogo-A Monoclonal Antibody in Amyotrophic Lateral Sclerosis: A Randomized, First-In-Human Clinical Trial
Source: PLoS One. 2014 May 19;9(5):e97803. doi: 10.1371/journal.pone.0097803 (PMC4026380; doi:10.1371/journal.pone.0097803)
Supplement: Table S2 — Assessment schedules for Part 2. AE, adverse event; ALSFRS-R, amyotrophic lateral sclerosis functional rating scale-revised; ECG, electrocardiogram; FU, follow-up; MUNE, motor unit number estimation; PK, pharmacokinetic; SAE, serious adverse event. *The precise timing of safety, functional assessments and PK blood sampling may have been altered during the course of the study based on emerging data. If the profile indicated that more sampling or assessments were needed, additional time points were to be added. Study assessments to follow PK sampling at end of infusion. (The 1-hour PK sample was collected directly at the end of the infusion, Cohorts 2–8). †Only SAEs related to study participation were collected prior to the start of the investigational product. Once the investigational product infusion began, all AEs and SAEs were collected until the last FU visit. ‡Continuous Lead II ECG commenced approximately 1 hour pre-dose until 24 hours post-Dose 1 and for 6 hours post-Dose 2. §In cohorts 6 and 7 the pre-dose muscle biopsy and blood sample were only done when the subject had passed all screening assessments and eligibility had been reconfirmed. This meant the pre-dose biopsy and blood sample could be done at any appropriate time before Day 1. The post-dose muscle biopsy and blood sample were scheduled for collection at Week 8 (unless emerging data suggested the post-dose muscle biopsy and blood sample should have been collected at an alternative week). In cohort 8, muscle biopsies and blood sample were collected from subjects at pre-dose and at one time point after the first dose. Subjects were assigned for a post-dose muscle biopsy and blood sample collection at either Day 1 (+24 hours), Day 8 or Week 4 (Day 22–24) based on subject preference determined at screening (see Section 7.3). If the subject had the Day 1 (+24 hours) collection then the pre-dose muscle biopsy and blood sample were collected at least 8 days before Day 1. #The number and schedule of FU vi [file pone.0097803.s002.docx]

## Table S2. Assessment schedules for Part 2.

| Procedure | Assessments (Dose 2 received at Week 5 [Day 29])^*^ |
| --- | --- |
| ALSFRS-R | Screening; Weeks 4, 8, and 16 after initial dosing |
| Manual muscle strength test | Screening; Weeks 4, 8, and 16 after initial dosing |
| Electrophysiology (MUNE) | Screening; Weeks 4, 8, and 16 after initial dosing |
| Slow inspiratory vital capacity | Screening; Weeks 4, 8, and 16 after initial dosing |
| Adverse events^†^ | Screening; pre-dose; continuously on Day 1; Weeks 2, 4, and 5; continuously on Day 29 after second dose; Weeks 6, 8, 10, 12, and 16 after initial dosing. |
| Clinical laboratory tests | Screening; pre-dose; 24 hours after initial dosing; Weeks 2 and 5 after initial dosing; 6 hours after second dose; Weeks 6, 10, and 16 after initial dosing. |
| Vital signs (supine and standing)^‡^ | Screening; pre-dose; 1, 2, 3, 4, 6, 8, 12, and 24 hours after dosing; Weeks 2, 4, and 5 after initial dosing; 1, 2, 3, 4, 5, and 6 hours after second dose; Weeks 6, 8, 10, 12, and 16 after initial dosing. |
| 12-Lead ECG (QT and other intervals) | Screening; pre-dose; 2, 4, 8, 12, and 24 hours after dosing; Weeks 2, 4, and 5 after dosing; 2, 4, and 6 hours after second dose; Weeks 6, 10, and 16 after initial dosing. |
| Continuous ECG monitoring (telemetry or bedside ECG monitor)^‡^ | Pre-dose until the end of Day 1; and pre-dose until the end of Day 29. |
| Deltoid muscle biopsy^§^ (Cohorts 6 and 7) | Pre-dose; Week 4 after 2^nd^ dosing; i.e Week 8 after initial dosing. |
| Deltoid muscle biopsy**^¶^** (Cohort 8 only) | Pre-dose; 24 hours after dosing; and Weeks 2 and 4 after initial dosing. |
| Blood sample for biomarkers | Pre-dose; 24 hours after dosing; and Weeks 2, 4, and 8 after dosing. |
| Blood sample for immunogenicity^#^ | Pre-dose; Weeks 2, 5, 8, 10, 12, and 16 after dosing; FU visit. |
| Blood sample for pharmacokinetics | Pre-dose; 1, 10, and 24 hours after dosing; Weeks 2, 4, and 5; 1 and 6 hours after second dose; Weeks 8, 10, 12, and 16 after dosing; FU visit. |

AE, adverse event; ALSFRS-R, amyotrophic lateral sclerosis functional rating scale-revised; ECG, electrocardiogram; FU, follow-up; MUNE, motor unit number estimation; PK, pharmacokinetic; SAE, serious adverse event.

.

^*^The precise timing of safety, functional assessments and PK blood sampling may have been altered during the course of the study based on emerging data. If the profile indicated that more sampling or assessments were needed, additional time points were to be added. Study assessments to follow PK sampling at end of infusion. (The 1-hour PK sample was collected directly at the end of the infusion, Cohorts 2–8).

^†^Only SAEs related to study participation were collected prior to the start of the investigational product. Once the investigational product infusion began, all AEs and SAEs were collected until the last FU visit.

^‡^Continuous Lead II ECG commenced approximately 1 hour pre-dose until 24 hours post-Dose 1 and for 6 hours post-Dose 2.

^§^In cohorts 6 and 7 the pre-dose muscle biopsy and blood sample were only done when the subject had passed all screening assessments and eligibility had been reconfirmed. This meant the pre-dose biopsy and blood sample could be done at any appropriate time before Day 1. The post-dose muscle biopsy and blood sample were scheduled for collection at Week 8 (unless emerging data suggested the post-dose muscle biopsy and blood sample should have been collected at an alternative week).

**^¶^**In cohort 8, muscle biopsies and blood sample were collected from subjects at pre-dose and at one time point after the first dose. Subjects were assigned for a post-dose muscle biopsy and blood sample collection at either Day 1 (+24 hours), Day 8 or Week 4 (Day 22–24) based on subject preference determined at screening (see Section 7.3). If the subject had the Day 1 (+24 hours) collection then the pre-dose muscle biopsy and blood sample were collected at least 8 days before Day 1.

^#^The number and schedule of FU visits after Week 16 for each subject varied depending on plasma concentrations of ozanezumab reaching a low enough level to allow a final blood sample to have been taken for immunogenicity assays.
