# Supplementary material for: Safety, Pharmacokinetic, and Functional Effects of the Nogo-A Monoclonal Antibody in Amyotrophic Lateral Sclerosis: A Randomized, First-In-Human Clinical Trial
Source: PLoS One. 2014 May 19;9(5):e97803. doi: 10.1371/journal.pone.0097803 (PMC4026380; doi:10.1371/journal.pone.0097803)
Supplement: Table S3 — Primer and probe sets used in biomarker analyses. (DOCX) [file pone.0097803.s003.docx]

## Table S3. Primer and probe sets used in biomarker analyses.

| Gene | Forward Primer | Reverse Primer | Probe |
| --- | --- | --- | --- |
| CHRNA1 | TCGGAACCCTAGCCGTGTTT | GGTAGGTTCCAGGGCAGAGC | CAGCAAGGATGAGCAGAAAATGAGCTGAGC |
| CHRNG | CTCGCTCTTCATCTGTGGCA | GTGGATCTCCAGGGAATGGC | AGCTGGCATCTTCCTCATGGCCCACTA |
| MUSK | CAACATCCTCTCCTGCCCTG | GCCCTCTCACACATGCGTTC | ATGTTGGAGCAAGCTGCCTGCAGACAG |
| NOGOA | CGGGCTCAGTGGATGAGAC | GCTCCTTCAAGTCCATATTTTCTG | TTGCTCTTCCTGCTGCATCTGAGCCTG |
| NOGOB | CGGGCTCAGTGGTTGTTGAC | TCACAGAGAGCAGGGCCAAG | TGAAAGCAGCAGGAATAGGCTGGCACC |
| NOGOC | TCCACACCCAGAAGACGTCAG | ACCCGTTTCCTCAACCGAAG | CAGTCCTGCTGCAGTTGTGCAGCAGAG |
| NOGOD | CGCTCCTCTGCAGTTGTTGAC | CAGGGCCAAGGCAATGTAGG | TGAAAGCAGCAGGAATAGGCTGGCACC |
| NOGOE | ACATTGACCCAACCGCAGTG | GGGTCTCATCAGAACTCTCTCC | CTCCAGTGGTGTGGTGCGTGGAGAAAT |
| RPL22 | GGATCAAAGTGAACGGAAAAGC | CGGATGTCACGGTGATCTTG | TTGCTCCTTTCGATGGTCACCACCC |
| GAPDH | CAAGGTCATCCATGACAACTTTG | GGGCCATCCACAGTCTTCTG | ACCACAGTCCATGCCATCACTGCCA |
| ACTB | GAGCTACGAGCTGCCTGACG | GTAGTTTCGTGGATGCCACAGGACT | CATCACCATTGGCAATGAGCGGTTCC |
| VPS26A | CAAACTTTCACCAGCGATTTGA | TTCTCCTGTTCAGTTCACATTTCAG | TCCAGAATCACAGGCATCTGCCGAA |
| PP1A | CATCTGCACTGCCAAGACTGA | CCACAATATTCATGCCTTCTTTCA | CCAAACACCACATGCTTGCCATCCA |
