# Supplementary material for: Safety, Pharmacokinetic, and Functional Effects of the Nogo-A Monoclonal Antibody in Amyotrophic Lateral Sclerosis: A Randomized, First-In-Human Clinical Trial
Source: PLoS One. 2014 May 19;9(5):e97803. doi: 10.1371/journal.pone.0097803 (PMC4026380; doi:10.1371/journal.pone.0097803)
Supplement: Table S4 — Summary of QTc values of potential clinical importance at any visit post-baseline. n, number of subjects; SD, single dose; RD, repeated dose. *Two doses, received 4 weeks apart. (DOCX) [file pone.0097803.s004.docx]

## Table S4. Summary of QTc values of potential clinical importance at any visit post-baseline.

|  |  | Ozanezumab SD | | | | |  |  | Ozanezumab RD^*^ | | |
| --- | --- | --- | --- | --- | --- | --- | --- | --- | --- | --- | --- |
|  | Placebo  n=10 | 0.01 mg/kg  n=6 | 0.1 mg/kg  n=6 | 1 mg/kg  n=6 | 5 mg/kg  n=6 | 15 mg/kg  n=6 |  | Placebo  n=9 | 0.5 mg/kg  n=9 | 2.5 mg/kg  n=9 | 15 mg/kg  n=9 |
| QTcB absolute value | | | | | | | | | | | |
| >450 to 480 msec | 0 | 0 | 0 | 0 | 1 (17) | 1 (17) |  | 1 (11) | 1 (11) | 3 (33) | 4 (44) |
| >480 to 500 msec | 0 | 0 | 0 | 0 | 0 | 0 |  | 0 | 0 | 0 | 0 |
| >500 msec | 0 | 0 | 0 | 0 | 0 | 0 |  | 0 | 0 | 0 | 0 |
| QTcB change from baseline | | | | | | | | | | | |
| >30 to 60 msec | 2 (20) | 2 (33) | 1 (17) | 1 (17) | 3 (50) | 0 |  | 4 (44) | 1 (11) | 1 (11) | 6 (67) |
| >60 msec | 0 | 0 | 0 | 0 | 0 | 0 |  | 0 | 0 | 0 | 0 |
| QTcF absolute value | | | | | | | | | | | |
| >450 to 480 msec | 0 | 0 | 0 | 0 | 0 | 0 |  | 0 | 0 | 0 | 2 (22) |
| >480 to 500 msec | 0 | 0 | 0 | 0 | 0 | 0 |  | 0 | 0 | 0 | 0 |
| >500 msec | 0 | 0 | 0 | 0 | 0 | 0 |  | 0 | 0 | 0 | 0 |
| QTcF change from baseline | | | | | | | | | | | |
| >30 to 60 msec | 1 (10) | 0 | 0 | 0 | 2 (33) | 0 |  | 1 (11) | 1 (11) | 0 | 1 (11) |
| >60 msec | 0 | 0 | 0 | 0 | 0 | 0 |  | 0 | 0 | 0 | 0 |

^*^Two doses, received 4 weeks apart.

n, number of subjects; SD, single dose; RD, repeated dose.
