# Supplementary material for: Safety, Pharmacokinetic, and Functional Effects of the Nogo-A Monoclonal Antibody in Amyotrophic Lateral Sclerosis: A Randomized, First-In-Human Clinical Trial
Source: PLoS One. 2014 May 19;9(5):e97803. doi: 10.1371/journal.pone.0097803 (PMC4026380; doi:10.1371/journal.pone.0097803)
Supplement: Table S5 — Summary of ALSFRS-R and MMT analyses. n, number of evaluable subjects; RD, repeat dose; SD, single dose; SE, standard error. ALSFRS-R, ALS functional rating scale-revised; CI, confidence interval; MMT, manual muscle strength test; Measured at Week 12 for SD study, Week 16 for RD study. *Two doses, received 4 weeks apart. (DOCX) [file pone.0097803.s005.docx]

## Table S5. Summary of ALSFRS-R and MMT analyses.

| Ozanezumab dose | Adjusted mean monthly slope | | | | Treatment  difference (SE) | 95% CI |
| --- | --- | --- | --- | --- | --- | --- |
|  | n | Treatment | n | Placebo |  |  |
| ALSFRS-R | | | | | | |
| SD 0.01 mg/kg | 6 | -0.55 | 10 | -0.92 | 0.38 (0.76) | -1.13, 1.89 |
| SD 0.1 mg/kg | 6 | -0.66 | 10 | -0.92 | 0.26 (0.76) | -1.25, 1.77 |
| SD 1 mg/kg | 6 | -0.57 | 10 | -0.92 | 0.36 (0.76) | -1.15, 1.87 |
| SD 5 mg/kg | 6 | -1.00 | 10 | -0.92 | -0.07 (0.76) | -1.59, 1.44 |
| SD 15 mg/kg | 6 | -0.92 | 10 | -0.92 | 0 (0.76) | -1.51, 1.51 |
| RD 0.5 mg/kg^*^ | 9 | -1.72 | 9 | -1.48 | -0.24 (0.69) | -1.62, 1.14 |
| RD 2.5 mg/kg^*^ | 7 | -1.62 | 9 | -1.48 | -0.14 (0.77) | -1.67, 1.39 |
| RD 15 mg/kg^*^ | 9 | -0.77 | 9 | -1.48 | 0.71 (0.69) | -0.67, 2.09 |
| MMT | | | | | | |
| SD 0.01 mg/kg | 6 | -2.95 | 10 | -3.43 | 0.48 (1.48) | -2.48, 3.44 |
| SD 0.1 mg/kg | 6 | -2.48 | 10 | -3.43 | 0.95 (1.47) | -2.00, 3.90 |
| SD 1 mg/kg | 6 | -0.26 | 10 | -3.43 | 3.17 (1.47) | 0.24, 6.10 |
| SD 5 mg/kg | 6 | -2.29 | 10 | -3.43 | 1.14 (1.47) | -1.79, 4.07 |
| SD 15 mg/kg | 6 | -1.49 | 10 | -3.43 | 1.95 (1.49) | -1.04, 4.93 |
| RD 0.5 mg/kg^*^ | 9 | -2.62 | 9 | -2.91 | 0.29 (1.28) | -2.29, 2.86 |
| RD 2.5 mg/kg^*^ | 7 | -1.33 | 9 | -2.91 | 1.58 (1.39) | -1.20, 4.36 |
| RD 15 mg/kg^*^ | 9 | -1.55 | 9 | -2.91 | 1.35 (1.27) | -1.22, 3.92 |

ALSFRS-R, ALS functional rating scale-revised; CI, confidence interval; MMT, manual muscle strength test;
n, number of evaluable subjects; RD, repeat dose; SD, single dose; SE, standard error.

Measured at Week 12 for SD study, Week 16 for RD study.

^*^Two doses, received 4 weeks apart.
