# Supplementary material for: Safety, Pharmacokinetic, and Functional Effects of the Nogo-A Monoclonal Antibody in Amyotrophic Lateral Sclerosis: A Randomized, First-In-Human Clinical Trial
Source: PLoS One. 2014 May 19;9(5):e97803. doi: 10.1371/journal.pone.0097803 (PMC4026380; doi:10.1371/journal.pone.0097803)
Supplement: Table S6 — Summary of SVC and MUNE analyses. CI, confidence interval; MUNE, motor unit number estimation; n, number of evaluable subjects; RD, repeat dose; SD, single dose; SE, standard error; SMUP, single motor unit potential; SVC, slow inspiratory vital capacity. Measured at Week 12 for SD study, Week 16 for RD study. *Two doses, received 4 weeks apart. (DOCX) [file pone.0097803.s006.docx]

## Table S6. Summary of SVC and MUNE analyses.

| Ozanezumab dose | Adjusted mean %  change from screening | | | | Treatment  difference (SE) | 95% CI |
| --- | --- | --- | --- | --- | --- | --- |
|  | n | Treatment | n | Placebo |  |  |
| % predicted SVC | | | | | | |
| SD 0.01 mg/kg | 6 | -7.35 | 10 | -4.31 | -3.04 (9.92) | -22.77, 16.68 |
| SD 0.1 mg/kg | 6 | -4.75 | 10 | -4.31 | -0.44 (9.89) | -20.11, 19.22 |
| SD 1 mg/kg | 6 | -1.30 | 10 | -4.31 | 3.01 (10.07) | -17.01, 23.04 |
| SD 5 mg/kg | 5 | -5.49 | 10 | -4.31 | -1.18 (10.45) | -21.93, 19.56 |
| SD 15 mg/kg | 6 | 7.69 | 10 | -4.31 | 12.00 (11.17) | -10.20, 34.20 |
| RD 0.5 mg/kg^*^ | 8 | -12.24 | 8 | -12.71 | 0.47 (9.28) | -17.96, 18.89 |
| RD 2.5 mg/kg^*^ | 6 | -22.99 | 8 | -12.71 | -10.28 (9.80) | -29.71, 9.16 |
| RD 15 mg/kg^*^ | 9 | -3.13 | 8 | -12.71 | 9.58 (9.13) | -8.56, 27.72 |
| MUNE estimated number of motor units | | | | | | |
| SD 0.01 mg/kg | 6 | -21.89 | 10 | -28.22 | 6.33 (20.86) | -35.12, 47.78 |
| SD 0.1 mg/kg | 6 | -27.77 | 10 | -28.22 | 0.44 (21.110 | -41.51, 42.40 |
| SD 1 mg/kg | 6 | -45.07 | 10 | -28.22 | -16.85 (22.72) | -62.01, 28.30 |
| SD 5 mg/kg | 5 | -54.19 | 10 | -28.22 | -25.97 (21.80) | -69.25, 17.31 |
| SD 15 mg/kg | 6 | -29.99 | 10 | -28.22 | -1.77 (21.17) | -43.83, 40.29 |
| RD 0.5 mg/kg^*^ | 8 | -26.09 | 8 | -43.57 | 17.48 (19.58) | -21.38, 56.34 |
| RD 2.5 mg/kg^*^ | 6 | -34.48 | 8 | -43.57 | 9.09 (20.52) | -31.60, 49.79 |
| RD 15 mg/kg^*^ | 9 | -37.36 | 8 | -43.57 | 6.21 (19.20) | -31.92, 44.34 |
| MUNE mean SMUP amplitude | | | | | | |
| SD 0.01 mg/kg | 6 | 67.68 | 10 | 18.95 | 48.73 (28.25) | -7.24, 104.71 |
| SD 0.1 mg/kg | 6 | 25.87 | 10 | 18.95 | 6.92 (28.50) | -49.55, 63.40 |
| SD 1 mg/kg | 6 | 16.98 | 10 | 18.95 | -1.97 (29.40) | -60.24, 56.30 |
| SD 5 mg/kg | 5 | 95.88 | 10 | 18.95 | 76.93 (30.59) | 16.36, 137.51 |
| SD 15 mg/kg | 6 | 25.79 | 10 | 18.95 | 6.84 (28.88) | -50.39, 64.07 |
| RD 0.5 mg/kg^*^ | 8 | 20.34 | 8 | 19.12 | 1.22 (27.75) | -53.73, 56.17 |
| RD 2.5 mg/kg^*^ | 6 | 20.69 | 8 | 19.12 | 1.57 (28.84) | -55.50, 58.64 |
| RD 15 mg/kg^*^ | 9 | 12.94 | 8 | 19.12 | -6.18 (26.40) | -58.47, 46.11 |

CI, confidence interval; MUNE, motor unit number estimation; n, number of evaluable subjects; RD, repeat dose; SD, single dose; SE, standard error; SMUP, single motor unit potential; SVC, slow inspiratory vital capacity.

Measured at Week 12 for SD study, Week 16 for RD study.

^*^Two doses, received 4 weeks apart.
