# Supplementary material for: Safety, Pharmacokinetic, and Functional Effects of the Nogo-A Monoclonal Antibody in Amyotrophic Lateral Sclerosis: A Randomized, First-In-Human Clinical Trial
Source: PLoS One. 2014 May 19;9(5):e97803. doi: 10.1371/journal.pone.0097803 (PMC4026380; doi:10.1371/journal.pone.0097803)
Supplement: Methods S1 — (DOC) [file pone.0097803.s007.doc]

# METHODS S1

The inclusion criterion regarding onset of muscle weakness was originally within 36 months. This was changed to within 60 months, by protocol amendment. The maximum dose in Part 2 of the protocol was also increased by amendment from 7.5 (twice) to 15 mg/kg (twice).

## Detailed methodology for pharmacokinetics

Plasma samples were analyzed for ozanezumab concentrations using a specific and validated chemiluminescent immunoassay. Plasma (10 µL) was diluted 10-fold with assay buffer. The lower limit of quantification (LLQ) for ozanezumab was 100 ng/mL and a higher limit of quantification (HLQ) of 1500 ng/mL.

Human muscle sample pharmacokinetic (PK) analyses were carried out by Alliance Pharma, Inc., using a validated analytical method based on an enzyme-linked immunosorbent assay method applicable to quantification within a nominal range of 25 ng/mL to 1500 ng/mL.

## Detailed methodology for immunogenicity analyses

The presence of anti-ozanezumab antibodies in human serum samples was determined using an analytically validated immuno-electrochemiluminescent (ECL) assay. Briefly, the ECL assay procedure involves mixing biotinylated ozanezumab, ruthenylated ozanezumab, and test sample. This mixture was incubated overnight at room temperature, before being transferred to a 96-well, streptavidin-coated plate (Meso Scale Discovery [MSD]) and incubated for 1 hour at room temperature. The plate was washed and read buffer was added. Finally, the plate was read on the Sector Imager 6000 (MSD). The resulting ECL signal is proportional to the amount of anti-ozanezumab antibodies in the sample.

Samples were tested for anti-ozanezumab antibodies in a multi-tier format, using the ECL assay procedure. First, all serum samples were assessed in an anti-ozanezumab binding antibody screening assay to identify potential positive samples. If a test sample was identified as a potential positive, the sample was tested in a confirmation assay to determine the specificity of the response to ozanezumab. Any samples that were confirmed positive were further tested in a titration assay to quasi-quantitate the level of anti‑ozanezumab antibodies. The incidence and titer values of confirmed positive samples were reported.

## Detailed methodology for biomarker analyses

### Ribonucleic acid (RNA) expression research

RNA expression studies were conducted on muscle biopsy samples using both quantitative reverse transcriptase polymerase chain reaction, and whole genome microarray analysis. Muscle samples were sent to GeneLogic, Gaithersburg, Maryland, USA, for microarray and quantitative reverse transcriptase polymerase chain reaction (PCR) analysis under Good Laboratory Practice conditions.

### Muscle RNA isolation

Human open muscle biopsy samples were collected in tissue protect tubes (Qiagen), before shipping to GeneLogic. Tissue biopsies were homogenized in 1 mL of Trizol reagent (Invitrogen) or at 50 mg/mL if the biopsy was greater than 50 mg, as per manufacturer’s instructions. One-fifth volume of chloroform was added and the lysate mixed and centrifuged, before adding an equal volume of 70% ethanol to aqueous phase and purifying the RNA using a Qiagen RNeasy Mini-column with on-column deoxyribonuclease I treatment. RNA was eluted using ribonuclease-free water, RNA quality assessed using an Agilent Bioanalyser and yield quantified by spectrophotometry.

### Muscle RNA microarray analysis

All RNA specimens were processed according to Gene Logic standard operating procedures (SOPs). The RNA was prepared into labeled target using standard NuGEN Ovation v2 processing with whole blood reagent. A quality control evaluation of target preparation yield and sample purity was performed according to Gene Logic SOPs. Fragmentation of labeled target was performed according to SOP, followed by chip hybridization and data generation on Affymetrix HG_U133 Plus 2.0 GeneChip microarrays. Quality control review of the microarray data was performed according to Gene Logic SOP.

### Muscle RNA TaqMan analysis

Thirteen real-time PCR assays were run, eight candidate biomarkers (cholinergic receptor, nicotinic, alpha 1 [CHRNA1], cholinergic receptor, nicotinic, gamma [CHRNG], muscle-specific kinase [MUSK], NOGO-A, NOGO-B, NOGO-C, NOGO-D, NOGO-E) and five housekeeper genes (actin, beta [ACTB], glyceraldehyde-3-phosphate dehydrogenase [GAPDH], peptidylprolyl isomerase A [PPIA], RPL22 and VPS26A). Primer and probe sequence are listed in Table S3.

Two hundred and sixty-four nanograms of RNA was converted to complementary deoxyribonucleic acid (cDNA) in triplicate using the ABI High Capacity cDNA Reverse Transcription Kit. RNA was arrayed in three replicate 96-well plates in a randomized order and cDNA converted following manufacturer’s instructions. Following cDNA conversion, each cDNA was diluted to 5 ng/μL. Two microlitres (10 ng) of cDNA were transferred from 96-well format to 384-well format for TaqMan analysis. Ten microliters of reaction were produced containing 5 μL of 2 x Applied Biosystems Universal Mastermix, 1 μL of GlaxoSmithKline-supplied primer mix (1 μM of probe, 9 μM of forward primer and 9 μM of reverse primer), 2 μL of template and 2 μL of water. Plates were cycled on an ABI7900 TaqMan machine using standard TaqMan thermal cycling conditions: 50°C for 2 minutes, 95°C for 10 minutes, followed by 40 cycles of 95°C for 15 seconds and 60°C for 1 minute.

PCR primers and probes were designed, tested in duplicate 10 μL reactions with a 10-fold dilution series of human genomic DNA (1^e5^ to 1^e1^ copies) and a no template control. All primer/probe sets passed quality assessment and were suitable for use in the study (Table S3).

Data were collected and analyzed using Sequence Detection System v2.4. Data were analyzed using a threshold of 0.1 and automatic baseline. Text files were exported with cycle thresholds and abundances (calculated from genomic standards).

## Protein research

### Muscle Nogo-A expression analysis

Human open muscle biopsy samples were collected and immediately frozen at -80°C, before shipping to Alliance Pharma, Inc. Malvern, Pennsylvania, PA, USA. Briefly, samples were homogenized in lysis buffer (55 mM Tris-HCl, 165 mM NaCl, 10% Triton, 1 mM EDTA, Halt protease and phosphatase inhibitor cocktail) and protein concentrations estimated using bicinchoninic acid (BCA) analysis. Lysates were shipped to GlaxoSmithKline and analysis of Nogo-A levels in muscle cell lysates was performed using MSD Technology. Briefly, MSD standard bind plates were spot-coated using 1 μL of 200 μg/mL AB62024 (Abcam) in spot coating buffer (25 mM hydroxyethyl-piperazine-ethane-sulphonate, 0.015% Triton-x 100). Plates were allowed to air dry for 48 hours on the bench, before covering with a plate sealer and storing at 4°C until the day of use. Lysates were diluted to 1.1 μg/mL total protein in lysis buffer, 25 μL (27 μg) was added to plates and incubated for 2 hours at room temperature on a plate shaker at approximately 750 rpm. Plates were washed three times with 175 μL of wash buffer (PBS, 0.05% Tween 20), and 25 μL of 1 μg/mL Sulfo-tagged AF3515 (R&D system-tag labeled by GlaxoSmithKline) in assay buffer (PBS, 0.5% bovine serum albumin, 0.1% Tween-20) was added to the plates. Plates were incubated for 2 hours on the plate shaker at approximately 750 rpm at room temperature. Plates were washed three times with 175 μL of wash buffer, 150 μL of 2X Read Buffer T (MSD) added and plates read immediately on the Sector 6000 MSD plate reader. Results were back-calculated using a seven-point standard curve generated from recombinant Nogo-A extracellular domain (GRITS 32973) in lysis buffer (0.048–200 ng/mL).

### Plasma protein expression analysis

Plasma samples were collected pre- and post-dose to estimate the level of expression of candidate soluble fragments of NOGO-A. Analysis of NOGO-A levels in plasma were performed using two independent immunoassays using MSD Technology. The assay used for analysis of NOGO-A levels in the lysate was also used to assess NOGO-A fragment levels in plasma. In addition, a second assay was developed using internally discovered anti-NOGO-A antibody H as capture, with a second internally discovered antibody C for detection. Assays were run using 25 μL of neat plasma according to the same basic methodology used for the lysate assay, but standard curves were generated using recombinant NOGO-A extracellular domain in plasma samples immunologically depleted for NOGO-A.

**Immunohistochemistry and laser scanning cytometry**

Frozen sections of muscle biopsies were examined by immunohistochemistry (IHC) and laser scanning cytometry (LSC) for expression of Nogo-A and ozanezumab. These analyses comprised: Nogo-A levels (percentage of total tissue [membrane and non-membrane] containing Nogo-A); ozanezumab levels (percentage of membrane containing drug); and % co-localization levels (percentage of membrane Nogo-A co-localized with drug). For Cohorts 7 and 8 (but not 5), sections were analyzed in triplicate for each biopsy. Sections were immunofluorescently labeled with antibodies against Nogo-A, non-neutralizing anti-idiotype against ozanezumab, and gamma sarcoglycan using the Intellipath Automated IHC instrument (Biocare Medical). Briefly, sections were fixed in 10% neutral buffered formalin for 10 minutes then washed in deionized water. Non-specific staining was reduced using Background Sniper (Biocare Medical) for 10 minutes. Next, the sections were incubated for 60 minutes in a primary antibody cocktail containing sheep anti-Nogo-A at 2 µg/mL (AF3515, R&D Systems), mouse anti-idiotype 9G12 (generated by GlaxoSmithKline) at 74 µg/mL and rabbit anti-gamma sarcoglycan at 0.75 µg/mL (HPA007476, Sigma). Primary antibodies were detected by incubating the sections for 30 minutes in a secondary antibody cocktail containing donkey anti-sheep Alexa 647 at 10 µg/mL (A21448, Invitrogen) and biotinylated horse anti-mouse ready-to-use (HM606, Biocare Medical) and donkey anti-rabbit 488 at 10 µg/mL (A21206, Invitrogen). Lastly, sections were incubated for 30 minutes in streptavidin Alexa 555 at 10 µg/mL (S32355, Invitrogen).

An iCyte LSC cytometer (CompuCyte Corp.) was used to measure gamma sarcoglycan stained membrane, anti-idiotype ozanezumab, and Nogo-A positive areas of the muscle biopsy sections. A mosaic scan (10x magnification with 20 µm step size) was performed to identify sections; then four 1x1 mm regions were manually placed over each section. High-resolution field scans (40x magnification with 0.5 µm step size) were examined from each region. Area, integral, and intensity measurements were collected for each high-resolution scan field using random segmentation elements (Phantom Contours). Gamma sarcoglycan staining was used to separate Nogo-A and ozanezumab associated with the membrane from Nogo-A and ozanezumab that was expressed in the muscle fibers using a process called “gating”. Gating is performed by creating scatter plots that are used to display the distribution of the phantom counted elements for each biomarker. For Cohorts 5 and 6, each plot was created independently and maxpixel was plotted against intensity for Nogo-A, gamma sarcoglycan, and ozanezumab. A gate was applied to each scatter plot by creating a region on the plots that captured events that were above the average amount of background noise in the placebo groups. The amount of ozanezumab that was co-localized with Nogo-A on the membrane was determined by plotting the intensities of ozanezumab that was co-localized with gamma sarcoglycan with Nogo-A that was also co-localized with gamma sarcoglycan. This method was applied on one section from each biopsy. For Cohorts 7 and 8, this method was further refined to increase the accuracy of the data and to evaluate the amount of variation of each marker within a biopsy. The refinements to the scatter plots included the plotting of intensities of each marker against each other rather than intensity versus maxpixel. This method places each marker on the same scale and helps to avoid over- or under-estimating the levels of each marker. In addition, the method used to create the gates on each scatter plot was adjusted. Instead of creating a new plot for each gate, a sub-plot was created from the origin or parent plot. This was determined to be a more accurate method and ensured that data were not inadvertently lost due to the creation of new regions on a new plot. This method was used to measure the expression of each marker in three sections from each biopsy rather than a single section. This was useful to estimate the distribution of the markers within a biopsy.

## Detailed methodology for manual muscle testing and motor unit number estimation

Manual muscle testing strength (MMT) was performed using the Medical Research Council (MRC) scale. The following muscles/actions were tested: thumb adduction, terminal phalange flexion, hand flexion, forearm flexion, arm abduction, plantar flexion of toes, dorsiflexion of toes, leg flexion, thigh flexion, thigh abduction, neck flexion, and neck extension.

The MRC scale used to score/grade the MMT assessments was as follows: 0. No movement; 1. Palpable contraction, no visible movement; 2. Movement but only with gravity; 3. Movement against gravity; 4. Movement against resistance but weaker than normal; 5. Normal power.

The total score was calculated as the sum of the individual muscle group scores for each of the 22 muscles, i.e., total score range from 0 to 110.

A modification of the automated multiple point stimulation technique was used in this study. At each visit, the motor unit number estimation (MUNE) procedure was performed on either the left or right arm, at either the median or ulnar nerve. The compound motor unit action potential (CMAP) was measured at the wrist. Then at each stimulus location, up to three increments were measured. These data were used to calculate one estimate for the number of motor units per visit.

The amplitude of the third response at each site was summed, then divided by 9 to yield the average single motor unit potential (SMUP) amplitude. This amplitude was divided into the maximum CMAP amplitude to yield the MUNE.

There were two MUNE endpoints: i) number of motor units estimated and ii) size of motor units, specifically calculated as mean SMUP amplitude. The number of motor units was estimated at each visit as follows: CMAP x 1,000/mean SMUP amplitude, where: CMAP was measured in millivolts (mV), and SMUP amplitude was measured in microvolts (μV). Mean SMUP amplitude was calculated as follows: Σ maximum increment at each stimulus location/total number of increments across all stimulus locations.

## Details of statistical analysis of functional endpoints

For amyotrophic lateral sclerosis functional rating scale-revised and MMT, slopes (monthly rate of decline) were calculated at each post-baseline visit as: change from screening at post-baseline visit/[(no. of days between screening and post-baseline visit/30.5)]. For both endpoints, an exploratory analysis using a random coefficients regression model was performed to determine the monthly rate of decline and to make planned contrasts of the slopes between each dose and placebo. Scores were fitted as the response variable with fixed effects of treatment, age, gender, time and treatment by time; subject was fitted as a random effect. In each cohort, the effect of treatment group was examined using the treatment by time interaction.

An exploratory analysis was also performed to determine the percentage change from screening in % predicted slow inspiratory vital capacity and both MUNE endpoints. Each response variable was analyzed using a mixed effects repeated measures analysis of variance (ANOVA) model, with treatment, age, gender, visit, treatment by visit interaction and screening value by visit interaction as fixed effects, with subject as a random effect. In each cohort, the effect of treatment was examined using the treatment by time interaction. Planned contrasts were made within the ANOVA model between each dose and placebo.
