# Supplementary material for: Safety, Pharmacokinetic, and Functional Effects of the Nogo-A Monoclonal Antibody in Amyotrophic Lateral Sclerosis: A Randomized, First-In-Human Clinical Trial
Source: PLoS One. 2014 May 19;9(5):e97803. doi: 10.1371/journal.pone.0097803 (PMC4026380; doi:10.1371/journal.pone.0097803)
Supplement: Results S1 — (DOCX) [file pone.0097803.s008.docx]

# RESULTS S1

## Additional details of clinical and laboratory findings

Of the subjects with elevated glucose, one (single dose [SD] 5 mg/kg ozanezumab) was recorded as having diabetes and had a urinalysis glucose value of 3+ at the same time point. Another subject (repeat dose [RD] 15 mg/kg ozanezumab) had blood glucose values above the upper limit of normal at screening and at several other time points, while the value of potential clinical importance (PCI) occurred only at Week 16. Two subjects (SD 1 mg/kg ozanezumab and RD 0.5 mg/kg ozanezumab) had blood glucose values of PCI at 24 hours after the first dose; these were isolated occurrences and all other glucose values recorded for these two subjects were within the normal range.

Treatment-emergent hematology PCI values were reported in four subjects: three subjects in the SD cohorts (two subjects receiving 0.1 mg/kg, and one receiving 5 mg/kg ozanezumab) and one subject in the RD 15 mg/kg ozanezumab group. The PCI abnormalities were decreased lymphocytes (4 subjects) and decreased white blood cell count (1 subject). The lymphocyte decreases of PCI first occurred at least 3 weeks after dosing in three of the subjects. One of these subjects (SD 0.1 mg/kg ozanezumab) had lymphocyte values below normal prior to dosing and at other time points, while the PCI values occurred only at Weeks 4 and 6. The fourth subject was in the SD 5 mg/kg ozanezumab group and had a PCI lymphocyte value at 24 hours post-dose; this was an isolated occurrence and except for a slightly decreased value (not of PCI) at Week 12, all other lymphocyte values in this subject were within the normal range.
