# Supplementary material for: Safety, Pharmacokinetic, and Functional Effects of the Nogo-A Monoclonal Antibody in Amyotrophic Lateral Sclerosis: A Randomized, First-In-Human Clinical Trial
Source: PLoS One. 2014 May 19;9(5):e97803. doi: 10.1371/journal.pone.0097803 (PMC4026380; doi:10.1371/journal.pone.0097803)
Supplement: Protocol S1 — Redacted protocol. (PDF) [file pone.0097803.s010.pdf]

Division: Worldwide Development
Retention Category: GRS019
Information Type: Protocol Amendment

**Title:**	A Single and Repeat Dose Escalation Study of the Safety, Pharmacokinetics and Pharmacodynamics of GSK1223249 in Patients with ALS.

Compound Number: GSK1223249

Effective Date: 10-DEC-2010

Protocol Amendment Number: 04

Description:

This is the first-time-in-human (FTIH) phase I/IIa study of GSK1223249, a humanised monoclonal antibody against Nogo-A, a neurite outgrowth inhibitor hypothesised to be involved in the pathophysiology of amyotrophic lateral sclerosis (ALS) and some other neurodegenerative disorders. This study will be a randomized, placebo-controlled, double-blind, sequential dose escalation, 2-part fusion protocol. Approximately 76 patients with ALS will be enrolled. In Part 1, single escalating intravenous (i.v.) doses of GSK1223249 are planned to be evaluated in 5 sequential patient cohorts (2 placebo and 6 active in each cohort) to determine single dose safety and pharmacokinetics (PK). Part 2 will also be of a sequential dose escalating design, but patients in each of the planned 3 cohorts (3 placebo, 9 active in each cohort) will receive 2 repeat i.v. doses approximately 4 weeks apart, where safety and PK will also be evaluated. In two cohorts in Part 1 and all cohorts in Part 2, blood samples and skeletal muscle biopsies will be taken from patients before and after at specific times post dosing to demonstrate whether or not GSK1223249 binds to its target and produces any measurable pharmacodynamic effect. Patients in both parts will receive their first dose in a hospital-based unit where they will be monitored for at least 24 hours post-dose before being discharged to be followed on an out-patient basis. In each cohort in part 1, the first four subjects will be dosed in a staggered manner such that only one will receive the dose in any 24 hours. Dosing of the first four subjects in the first cohort of part 2 will also be staggered in a similar manner.

Subject: FTIH Study, GSK1223249, ALS, Single and Repeat Dose Escalation, Safety, Pharmacokinetics, Pharmacodynamics, Fusion protocol, Muscle Biopsies

Author: [redacted]

Copyright 2010 the GlaxoSmithKline group of companies. All rights reserved. Unauthorised copying or use of this information is prohibited.

Revision Chronology:

[redacted]	2008-DEC-01	Original
[redacted]	2009-APR-08	Amendment No.: 01<br><br>This amendment is required for the following reasons:-<br><br>To introduce text explaining the procedure to be followed in order to inform all Investigators in the event that a serious adverse event is reported to GSK.<br><br>To combine 2 bullet points in Appendix 3 which have related meaning.<br><br>To add urine alcohol test to Section 6.2.7 to enable sites to have choice of test other than just breath test.<br><br>To change Medical Monitor details.<br><br>Other minor changes to clarify wording and timing of pre-dose muscle biopsy.

[redacted]	2009-NOV-17	Amendment 02.:<br><br>This amendment is required for the following reasons:-<br><br>To include the IND number.<br><br>To allow the muscle biopsies in cohort 3 to be voluntary rather than required.<br><br>To add clarity by amending the text around the cumulative data required for dose escalation.<br><br>To change timing of the interim analysis between part 1 and part 2 to after the end of cohort 4 with the intention of starting cohort 5 and cohort 6 at the same time.<br><br>To allow the post dose muscle biopsy in part 2 to be taken at an alternative time point to week 8, if required depending on emerging biomarker data.<br><br>To make patient visits from week 4 more flexible for the subjects by increasing the range either side of the visit date.<br><br>To reduce the exclusion period from vaccination to entry into the study.<br><br>To modify exclusion criterion 14 to be clear about which subjects the criteria applies to.<br><br>To remove a sentence relating to full disclosures of ECG, which is not relevant for this study.<br><br>To add additional text to the Liver Chemistry Testing Procedure in the light of new GSK standards.<br><br>Additional author added.

[redacted]	2010-JAN-25	Amendment No.: 3
		This amendment is required for the following reasons:-
		To extend the period on onset of muscle weakness to study entry in the inclusion criteria.
		Modify SVC inclusion criteria to allow investigator discretion with regard to study entry.
		Remove BMI inclusion criteria and modify to allow investigator discretion for entry into study.
		To collect and database subject's handedness.
		Add a sentence to confirm that the data from part 1 of the study will be reviewed before cohort 7 begins.
		Clarification of the data consolidation for the interim analysis.

[redacted]	2010-DEC-10	Amendment No.: 04 This amendment is required for the following reasons:-<br><br>To increase the dose in part 2, (cohort 8) from 7.5mg/kg to 15mg/kg based on emerging safety and tolerability data to thoroughly explore safety and tolerability of GSK1223249 at dose levels thought feasible to be taken forward into future studies.<br><br>For cohort 8: To change the post-dose muscle biopsy collection such that post-dose muscle biopsies are obtained at different intervals following the first dose (each subject still to have one post-dose muscle biopsy at either Day 1(+24hr), Day 8 or Week 4 (Day 22-28) to better characterize target-organ (muscle) pharmacokinetics.<br><br>To add clarity to Table 6 showing cohort 5 and 6 starting in parallel and cohort 8 post dose muscle biopsy scheduled before dose 2.<br><br>Section 1.2.2 Pre-Clinical Pharmacology, Section 1.2.3 Pre-Clinical Safety, Section 1.2.4 Pre-Clinical Pharmacokinetics and Section 1.3.2 Dose Rationale updated with new data.<br><br>To add clarity to the 1hr post dose PK sample collection time to ensure the sample is collected at the end of the infusion.<br><br>Clarification for inclusion criteria 4 and in Section 8.1 that patients should be on stable medications within 28 days prior to dosing.<br><br>Additional authors added

SPONSOR SIGNATORY:

[redacted]

10 Dec 2010

Date

Head, Translational Pharmacology & Discovery Medicine GlaxoSmithKline

SPONSOR/MEDICAL MONITOR INFORMATION PAGE

Medical Monitor and Sponsor Contact Information:

Role	Name	Day Time Phone Number	After-hours Phone/Cell/ Pager Number	Fax Number	GSK Address
Primary Medical Monitor	[redacted]	[redacted]	[redacted]	[redacted]	GlaxoSmithKline R&D Ltd [redacted]
Secondary Medical Monitor	[redacted]	[redacted]	[redacted]	[redacted]	GlaxoSmithKline R&D Ltd [redacted]
Tertiary Medical Monitor	[redacted]	[redacted]	[redacted]	[redacted]	GlaxoSmithKline [redacted]
SAE fax number[1]				[redacted]	

1. The primary mechanism for prompt (within 24 hours) reporting of SAEs to GSK will be the electronic data collection tool (InForm). If the electronic system is unavailable for longer than 24 hours, the site will use the paper SAE data collection tool and fax it to the GSK Medical Monitor at [redacted] Then the site will enter the SAE data into the electronic system as soon as it becomes available. Please see Section 11.8 for details.

Sponsor Registered Address:

GlaxoSmithKline Research & Development Limited
[redacted]

In some countries, the clinical trial sponsor may be the local GlaxoSmithKline affiliate company (or designee). If applicable, the details of the alternative Sponsor and contact person in the territory will be provided to the relevant regulatory authority as part of the clinical trial application.

Regulatory Agency Identifying Number(s): Eudract No. 2008-005040-16

IND No. 102832

INVESTIGATOR PROTOCOL AGREEMENT PAGE

I confirm agreement to conduct the study in compliance with the protocol, as amended by this protocol amendment.

Investigator Name:	
Investigator Address:	
Investigator Phone Number:	

Investigator Signature	Date

TABLE OF CONTENTS

PAGE

ABBREVIATIONS 12

1. INTRODUCTION 14
   1.1. Background 14
      1.1.1. Amyotrophic Lateral Sclerosis 14
      1.1.2. Nogo-A 14
      1.1.3. Nogo-A and ALS 16
   1.2. Non-Clinical Summary 16
      1.2.1. Summary of the Compound: GSK1223249 16
      1.2.2. Pre-Clinical Pharmacology 17
      1.2.3. Pre-Clinical Safety 21
      1.2.4. Pre-Clinical Pharmacokinetics and Product Metabolism 26
   1.3. Rationale 27
      1.3.1. Study Rationale 27
      1.3.2. Dose Rationale 28
   1.4. Summary of Risk Management 32

2. OBJECTIVE(S) AND ENDPOINTS 34

3. INVESTIGATIONAL PLAN 35
   3.1. Study Design/Schematic 35
   3.2. Discussion of Design 35
   3.3. Dose Adjustment/Stopping Criteria (Parts 1 and 2) 38
      3.3.1. Planned Doses and Data Required for Dose Escalation 38
      3.3.2. Stopping Criteria for Individual Subjects in Part 2 39
      3.3.3. Criteria for Suspension/Dose Adjustment/Stopping Study 40
      3.3.4. Criteria for Progression from Part 1 to Part 2 40
   3.4. Replacement of subjects 41
   3.5. Treatment Assignment 41
   3.6. Description of Investigational Product 42

4. STUDY POPULATION 50
   4.1. Number of Subjects 50
   4.2. Eligibility Criteria 50
      4.2.1. Inclusion Criteria for Parts 1 and 2 50
      4.2.2. Exclusion Criteria for Parts 1 and 2 51
      4.2.3. Other Eligibility Criteria Considerations 52

5. DATA ANALYSIS AND STATISTICAL CONSIDERATIONS 52
   5.1. Hypotheses and Treatment Comparisons 52
   5.2. Sample Size Considerations 53
      5.2.1. Sample Size Assumptions 53
      5.2.2. Sample Size Sensitivity 53
      5.2.3. Sample Size Re-estimation 53
   5.3. Data Analysis Considerations 53
      5.3.1. Interim Analysis 53
      5.3.2. Final Analyses 53

6. STUDY ASSESSMENTS AND PROCEDURES 56

6.1. Demographic/Medical History Assessments 56
6.2. Safety 56
6.2.1. Physical Examination 57
6.2.2. Neurological Examination 57
6.2.3. Vital Signs 57
6.2.4. Electrocardiogram (ECG) 57
6.2.5. Clinical Laboratory Assessments 58
6.2.6. Urine collection for the Detection of Undeclared Drugs 59
6.2.7. Alcohol Test 59
6.2.8. Pregnancy Test 60
6.3. Pregnancy 60
6.3.1. Time period for collecting pregnancy information 60
6.3.2. Action to be taken if pregnancy occurs 60
6.3.3. Action to be taken if pregnancy occurs in a female partner of a male study subject 60
6.4. Pharmacokinetics 61
6.4.1. Blood Sample Collection 61
6.4.2. Sample Analysis 61
6.5. Functional and Electrophysiological Assessments 61
6.5.1. Functional Assessments 61
6.5.2. Electrophysiological Assessment (MUNE) 62
6.6. Biomarker(s)/Pharmacodynamic Markers 62
6.6.1. Skeletal Muscle Biopsies and Blood Samples 62
6.6.2. Exploratory Biomarkers 63
6.7. Immunogenicity (Anti-Drug Antibodies) 64
6.8. Pharmacogenetics 64

7. LIFESTYLE AND/OR DIETARY RESTRICTIONS 64
7.1. Contraception Requirements 64
7.1.1. Female Subjects 64
7.1.2. Male Subjects 65
7.2. Alcohol and Tobacco 65
7.3. Activity 66

8. CONCOMITANT MEDICATIONS AND NON-DRUG THERAPIES 66
8.1. Permitted Medications 66
8.2. Prohibited Medications 66

9. COMPLETION OR EARLY WITHDRAWAL OF SUBJECTS 66
9.1. Subject Completion 66
9.2. Subject Withdrawal Criteria 66
9.3. Subject Withdrawal Procedures 67
9.3.1. Subject Withdrawal from Study 67
9.3.2. Subject Withdrawal from Investigational Product 67
9.4. Treatment After the End of the Study 67
9.5. Screening Failures 68

10. INVESTIGATIONAL PRODUCT(S) 68
10.1. Blinding 68
10.2. Blinding and Dose Escalation Committee (DEC) 68
10.3. Packaging and Labeling 69
10.4. Preparation/Handling/Storage/Accountability 69

10.5. Assessment of Compliance 70
10.6. Treatment of Investigational Product Overdose 70

11. ADVERSE EVENTS (AE) AND SERIOUS ADVERSE EVENTS (SAE) 70
11.1. Definition of Adverse Events 71
11.2. Definition of Serious Adverse Events 72
11.2.1. Clinical Laboratory Abnormalities and Other Abnormal Assessments as AEs and SAEs 73
11.3. Events Common to ALS 73
11.4. Method of Detecting AEs and SAEs 73
11.5. Recording of AEs and SAEs 74
11.6. Evaluating AEs and SAEs 74
11.6.1. Assessment of Intensity 74
11.6.2. Assessment of Causality 74
11.7. Follow-up of AEs and SAEs 75
11.8. Prompt Reporting of SAEs to GSK 75
11.9. Regulatory Reporting Requirements For SAEs 76

12. LIVER CHEMISTRY TESTING PROCEDURES 76

13. STUDY CONDUCT CONSIDERATIONS 78
13.1. Regulatory and Ethical Considerations, Including the Informed Consent Process 78
13.2. Quality Control (Study Monitoring) 79
13.3. Quality Assurance 80
13.4. Study and Site Closure 80
13.5. Records Retention 80
13.6. Provision of Study Results and Information to Investigators 81
13.7. Data Management 82

14. REFERENCES 83

APPENDICES 86
Appendix 1: Pharmacogenetic research 86
Appendix 2: Country Specific Requirements 90
Appendix 3: Events common to ALS 91
Appendix 4: Protocol Amendment Changes 92

ABBREVIATIONS

ADA	Anti Drug Antibodies
ADCC	Antibody Dependent Cellular Cytotoxicity
AE	Adverse Event
ALSFRS-R	Amyotrophic Lateral Sclerosis Functional Rating Scale-Revised
ALS	Amyotrophic Lateral Sclerosis
ANOVA	Analysis of Variance
AUC	Area under concentration-time curve
BIOL	Biology
CCSE	Centre for Study Science Excellence
CIB	Clinical Investigator's Brochure
CL	Systemic clearance of parent drug
CL/F	Apparent clearance following oral dosing
Cmax	Maximum observed concentration
CDC	Complement Dependent Cytotoxicity
CPSR	Clinical Pharmacology Study Report
CP-RAP	Clinical Pharmacology Reporting and Analysis Plan
CPMS	Clinical Pharmacology Modelling and Simulation
CRF	Case Report Form
CRO	Contract Research Organization
CV	Coefficient of variance
DB	Discovery Biometrics
DM	Discovery Medicine
DEC	Dose Escalation Committee
ECG	Electrocardiogram
EDC	Electronic data capture
EISR	Expedited Investigator Safety Report
FDA	Food and Drug Administration
FTIH	First time in humans
GCP	Good Clinical Practice
GSB	Global Safety Board
GSK	GlaxoSmithKline
HR	Heart rate
IB	Investigator's Brochure
ICH	International Conference on Harmonization of Technical Requirements for Registration of Pharmaceuticals for Human Use
IDMC	Independent Data Monitoring Committee
IDSL	Integrated Data Standards Library
IEC	Independent Ethics Committee
IND	Investigational New Drug
IP	Investigational Product
IRB	Institutional Review Board
IU	International Unit
IV or i.v.	Intravenous
Kg	Kilogram
LFTs	Liver function tests
LOQ	Limit of quantification

LLQ	Lower limit of quantification
MDC	Medicine Development Centre
MedDRA	Medical Dictionary for Regulatory Activities
Mg	Milligrams
mL	Milliliter
MMT	Manual Muscle Testing
MRC	Medical Research Council
MUNE	Motor Unit Number Estimation
MSDS	Material Safety Data Sheet
msec	Milliseconds
NQ	Non-quantifiable concentration measured as below LLQ
PD	Pharmacodynamic
PGx	Pharmacogenetics
PK	Pharmacokinetic
QC/QC'ed	Quality Control/Quality Controlled
RAP	Reporting and Analysis Plan
SA	Safety Assessment
SAE	Serious adverse event(s)
SAS	Statistical Analysis Software
SD	Standard deviation
SOP	Standard Operating Procedure
SPM	Study Procedures Manual
SUSAR	Suspected, Unexpected, Serious Adverse drug Reaction
SVC	Slow Inspiratory Vital Capacity
Tmax	Time of occurrence of Cmax
ULN	Upper limit of normal

Trademark Information

Trademarks of the GlaxoSmithKline group of companies
NONE

Trademarks not owned by the GlaxoSmithKline group of companies
Biacore
Chiron RIBA
WinNonlin

1. INTRODUCTION

1.1. Background

1.1.1. Amyotrophic Lateral Sclerosis

Amyotrophic Lateral Sclerosis (ALS) is a progressive neurodegenerative disease associated with loss of upper and lower motor neurons. It is the most common motor neuron disease in adults affecting approximately 33,300 people in the seven major markets (US, Japan, UK, Germany, France, Spain and Italy) with a prevalence rate of about 4.5 per 100,000. The typical age of onset is between 50 and 70 years, although ALS can sometimes occur at a younger age.

The primary pathophysiology of ALS is the selective dysfunction and death of neurons involved in vital motor pathways. This leads to spasticity, hyper-reflexia (upper motor neurons), weakness and muscle atrophy (lower motor neurons) [Mulder, 1986]. Diagnosis is made by clinical evaluation supported by laboratory methods. The clinical course of the disease is highly variable suggesting multiple factors contributing to the pathogenesis. The majority of patients (up to 90%) die in the first 5 years following onset [Bruijn, 2004] mainly from complications of respiratory failure. In addition to the motoric features other findings, such as depression and frontotemporal dementia are present in a proportion of ALS patients [Enayat, 1995].

Palliative treatment for ALS, including non-invasive ventilation, prolongs survival and improves or maintains quality of life [Andersen, 2005; Enayat, 1995]. At present, Riluzole is the only approved drug therapy for ALS. Riluzole is thought to reduce the glutamate drive on motor neurons and has demonstrated a marginal effect on functional decline with modest prolongation of median survival by about 2 to 3 months [Bensimon, 1994; Lacomblez, 1996; Miller, 2007]. Thus, there remains a great unmet medical need for a treatment that will be effective in slowing or reversing the decline of function and significantly prolonging survival in ALS.

1.1.2. Nogo-A

Nogo-A is a high molecular weight transmembrane protein that was initially identified as a potent myelin-associated inhibitor of axonal growth expressed mostly by oligidendrocytes. Nogo-A, myelin associated glycoprotein (MAG) and oligodendrocyte myelin glycoprotein (OMgp) bind to and signal through a multi-subunit receptor complex consisting of the Nogo66 receptor NgR1, the adaptor molecule Lingo-1 and effector components p75/Troy (see Figure 1). Stimulation of this complex at the surface of neurones leads to the intracellular activation of of the small GTPase RhoA that mediates actin depolymerisation and thereby the collapse or retraction of neurites [Walmsley, 2007; Pernet, 2008].

Figure 1 **Schematic representation of neuronal growth inhibitory proteins released upon injury and their known receptors**

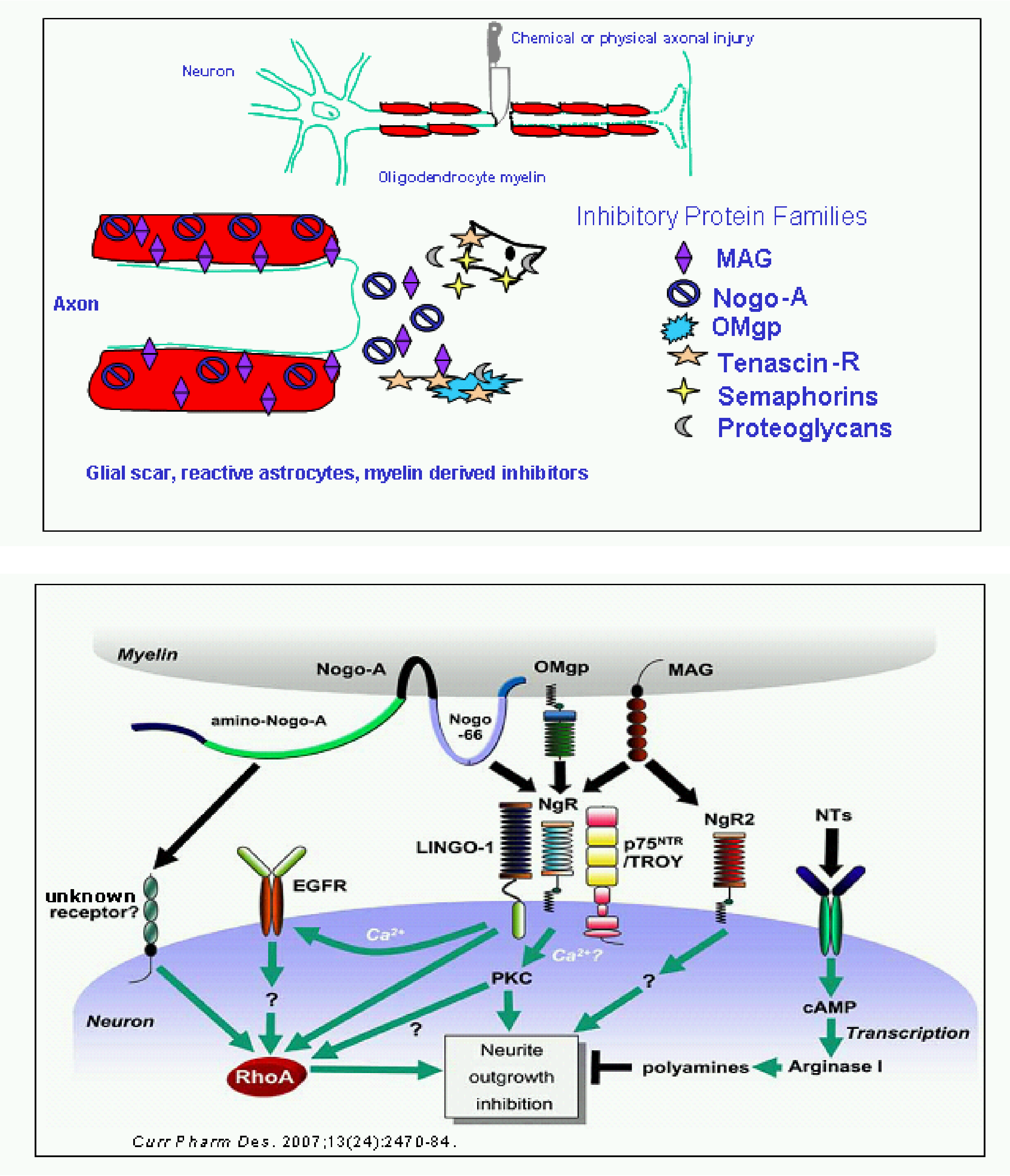

There are three isoforms of Nogo generated from a single gene. Nogo-A is mostly expressed by oligodendrocytes and to a lesser extent by neurones. Nogo-B is ubiquitously expressed and Nogo-C is highly expressed in the muscle and brain. The Nogo-A isoform has been found to be the most potent neurite outgrowth inhibitor.

1.1.3. Nogo-A and ALS

While Nogo-A is not appreciably expressed in healthy skeletal muscle, there is increasing evidence that abnormal expression of Nogo-A in skeletal muscle may be involved in the pathophysiology of ALS. In the SOD1 mouse model of ALS, Nogo-A is upregulated in skeletal muscle and causes neuromuscular junction repulsion [Jokic, 2006]. Nogo-A was detectable in skeletal muscle biopsies from ALS patients, but not in those from healthy controls or patients with sensorimotor peripheral neuropathy or myopathy [Dupuis, 2002]. Using semi-quantitative methods in a small group of patients, Jokic showed that expression of Nogo-A in skeletal muscle appears to correlate with the severity of ALS [Jokic, 2005]. In a group of 33 patients with spinal lower motor neuron syndrome (LMNS), Nogo-A expression was detected in 17 patients but not in another 16 patients. When these patients were followed up for 12 months, the presence of Nogo-A in skeletal muscle biopsy samples correctly identified those patients who further progressed to ALS with 91% accuracy, 94% sensitivity, and 88% specificity [Pradat, 2007]. Therefore the authors advocate adoption of Nogo-A expression in muscle as a diagnostic biomarker of ALS. Furthermore, in a mouse model of ALS, exogenous expression of Nogo-A in skeletal muscle promotes denervation and instability of the neuromuscular junction [Jokic, 2006]. These and other data are highly suggestive of a role of Nogo-A in the pathophysiology of ALS, although it is not clear to what extent Nogo-A may be involved in directly triggering the pathology.

GSK has developed a monoclonal antibody (mAb), GSK1223249, against Nogo-A which is to be developed as a drug therapy for neurodegenerative disorders where Nogo-A may be involved. Studies in the SOD1 mutant transgenic mouse model showed that this mAb significantly delayed the time to onset of symptoms and prolonged survival. It is therefore proposed to test the fully humanised version of this mAb in patients with ALS.

Currently available data from non-clinical studies with GSK1223249 is summarised in subsequent parts of this section. For further details about these data, please refer to the Investigators Brochure.

1.2. Non-Clinical Summary

1.2.1. Summary of the Compound: GSK1223249

GSK has identified a high affinity anti-Nogo-A monoclonal antibody (mAb) clone GSK577548 which has been successfully humanised to generate GSK1223249. The humanised mAb GSK1223249 displays the following key *in vitro* properties:

- Binds human Nogo-A with high affinity (0.64nM) to a defined epitope (VLPDIVMEAPLN) which has been mapped within the key inhibitory domain
- Binds native Nogo-A in human neuroblastoma cells and tissue sections
- Retains the species cross-reactivity profile of the parent antibody GSK577548 and recognises rat and cynomolgus Nogo-A with comparable affinity to human Nogo-A
- Has been engineered to disable Fc immune effector functionality

- Has a predicted low risk of clinical immunogenicity as determined using the Algonomics Epibase technology
- Is stable in PBS and human serum.

1.2.2. Pre-Clinical Pharmacology

Inhibition of Fc functionality

To improve the safety profile of the candidate, residues L235 and G237 within the CH2 domain of the heavy chain constant region [Kabat, 1991] were mutated to alanine residues [Brett, 1997] thus reducing the likelihood of triggering antibody-mediated immunological effector functions such an antibody dependent cellular cytotoxicity (ADCC) and complement dependent cytotoxicity (CDC). Figure 2 shows that GSK1223249 has significantly reduced C1q binding activity, compared to Campath-IgG1 (wild-type) and comparable to a Campath IgG1 Fc-mutated antibody ('Fc'-) and Campath IgG4. These data suggest that the CH2-domain mutations present in GSK1223249 will significantly reduce the likelihood of triggering ADCC and CDC.

Figure 2 GSK shows reduced C1q binding compared to Campath-IgG1

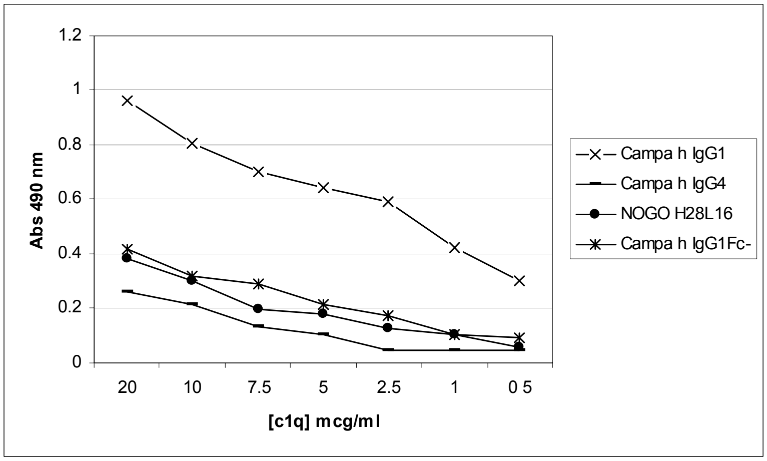

GSK1223249 shows reduced C1q binding. ELISA plates were coated with a fixed concentration of the purified humanised and control antibodies (1mcg/ml). Human C1q (Sigma, C0660) was incubated with the antibodies and bound C1q quantified using an anti-human c1q-HRP conjugate (The Binding Site, PP020X). The control antibodies are Campath IgG1, Campath IgG4 and Campath IgG1 Fc-.

Species Orthologue binding

The kinetics of binding of GSK1223249 to the various species orthologues of Nogo-A was determined using the Biacore. Table 1 shows the kinetics of binding where the recombinant Nogo-A was coupled directly to the CM5 chip. The binding kinetics for rat, cynomolgus monkey, squirrel monkey, marmoset and rabbit are very similar to that for human (range = 0.33-0.67nM).

Table 1 Binding kinetics of GSK1223249 to the recombinant orthologues of human Nogo-A

	**GSK1223249**		
Orthologue	*Ka*	*Kd*	KD (nM)
Cynomolgus (2 runs)	4.65e6 (7.47e5)	3.07e-3 (2.37e-4)	0.67 (0.06)
Rat (2 runs)	4.64e6 (2.34e5)	1.54e-3 (3.06e-5)	0.33 (0.01)
Marmoset (1 run)	4.2e6 (2.47e4)	3.02e-3 (5.09e-5)	0.626 (0.000)
Squirrel Monkey (1 run)	4.46e6 (6.08e4)	2.73e-3 (4.95e-6)	0.61 (0.000)
Rabbit	4.202e6 (NA)	2.271e-3 (NA)	0.541 (NA)
[1]Human	6.97e6 (6.62e5)	4.43e-3 (1.18e-3)	0.64 (0.15)

As determined using the Biacore T100. Approximately 140-180RUs of the various Nogo-A orthologues were captured to the CM5 chip by primary amine coupling. The antibody was flowed over at various concentrations (0.125-8nM). The values show the mean and standard deviation (in brackets) of 1-2 independent runs carried out in duplicate with each data set independently analysed prior to calculation of the mean and standard deviation.

1. For reference, the kinetics of binding to human GST-Nogo-A 5+6 is shown in the final row.

Neurite outgrowth inhibition

GSK1223249 was tested for its ability to neutralise the neurite-outgrowth (NO) inhibitory activity of Nogo-A in an assay that is based on quantifying NO. In order to test the neutralising activity of GSK1223249, wells coated with human recombinant Glutathione S-Transferase (GST)-Nogo-A5+6 (a region we have previously shown to recapitulate the inhibitory activity of full-length Nogo-A) were treated with varying concentrations of antibodies at 37ºC for 1h prior to the addition of rat post-natal day 8 primary cerebellar granule neurons. Control wells were coated with GST only. Average neurite length per neurite (NL/N) was measured for each well. Figure 3 shows the results for GSK1223249 and control IgG (control IgG, purified mouse IgG).

Figure 3 **GSK1223249 and control IgG in the neurite outgrowth assay**

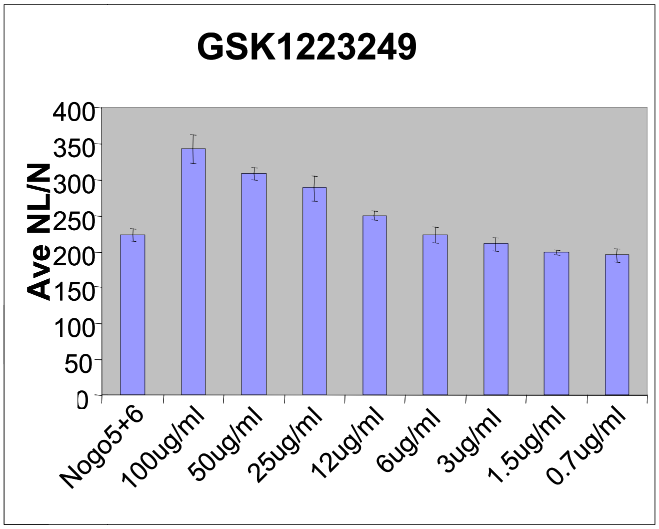

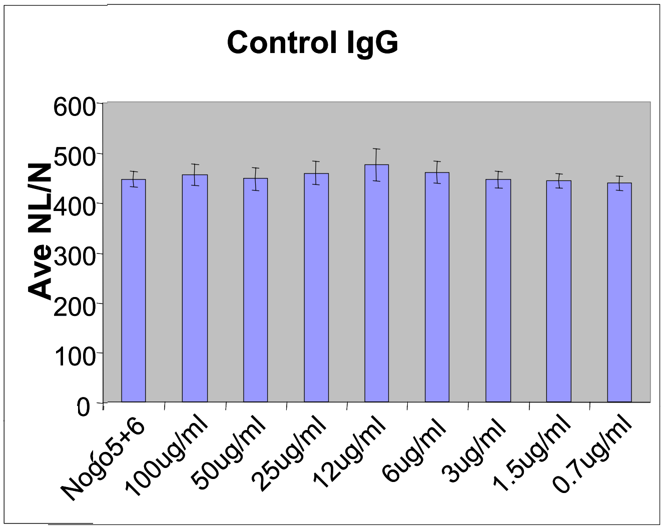

In these experiments a batch of control GST protein was used that failed to support robust outgrowth we had seen in previous experiments. In subsequent tests anti-Nogo-A antibodies failed to alter growth on this control substrate suggesting this was due to non-specific contaminants. These results have therefore been excluded for clarity. Increased neurite-outgrowth was only seen with GSK1223249. The effects were dose-dependent and statistically significant.

SOD1 mutant transgenic mouse efficacy studies

The cause or trigger of ALS is unknown at present. The majority of cases are 'sporadic' with 5-10% being familial. Approximately 20% of the familial ALS are caused by dominantly inherited mutations in the protein Cu/Zn superoxide dismutase (SOD1) [Rosen, 1993; Andersen, 2004]. Furthermore partially misfolded species of the SOD1 protein may be a common feature of both inherited and sporadic ALS [Gruzman, 2007]. Historically, the discovery of SOD1 mutations led to the generation of the first animal models of ALS. Several mouse lines were generated that overexpress ubiquitously mutant SOD1 (mSOD1) at levels sufficient to induce a motor neuron disease closely resembling human ALS [Gurney, 1994]. For these reasons, mSOD1 mice (particularly SOD1-G93A)

are the most widely studied animal models of ALS. A large consensus exists now on the fact that mutations in SOD1 confer a gain of function to the mutant enzyme. This notion was early established when it was reported that mice knocked out for wild-type SOD1 do not display motor neuron disease [Reaume, 1996].

The first data linking Nogo with ALS was obtained in a SOD1 mouse differential expression study designed to identify early markers of disease pathology which were confirmed with subsequent molecular analyses at the mRNA and protein levels [Dupuis, 2002]. This study went on to confirm that the same changes could be seen in biopsy samples from clinically confirmed ALS patients but not those from other muscle diseases. Subsequent larger studies have since confirmed that the level of Nogo-A up-regulation appears to correlate well with disease severity as measured using the ALS-FRS scale [Jokic, 2005] and may even potentially be a prognostic biomarker [Pradat, 2007].

GSK has extended these studies and shown that pharmacological blockade of Nogo-A using the GSK1223249 mouse parental antibody (GSK577548) can attenuate signs of disease in SOD1 mutant transgenic mice.

Figure 4 and Figure 5 respectively show the cumulative proportion surviving and cumulative proportion symptom free following treatment with GSK577548 (3mg/kg and 30mg/kg) in comparison to PBS and a Control IgG (3.0mg/kg). The results of this study show that 3mg/kg GSK577548 significantly increases age at death by 16.4 days compared to PBS (95% CI 0.3 to 32.6 days, P<0.05) and that 3mg/kg GSK577548 significantly increases age at onset by 15.5 days compared to PBS (95% CI 2 to 29 days, P<0.05). This was confirmed using another anti-Nogo-A monoclonal antibody 2C4 which binds to a distinct epitope. (Figure 4 and Figure 5).

Figure 4 Cumulative proportion surviving following treatment with 3mg/kg and 30mg/kg GSK577548 (2A10) (Dosing solutions at 0.3mg/ml and 3mg/ml)

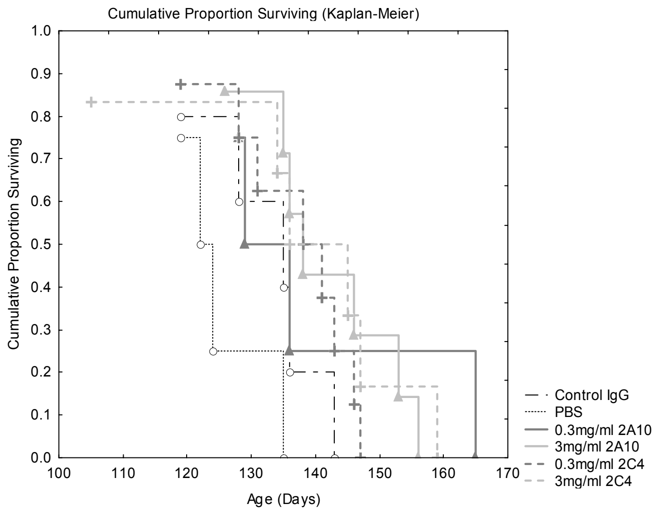

Figure 5 Cumulative proportion symptom free following treatment with 3mg/kg and 30mg/kg GSK577548 (2A10) (Dosing solutions at 0.3mg/ml and 3mg/ml)

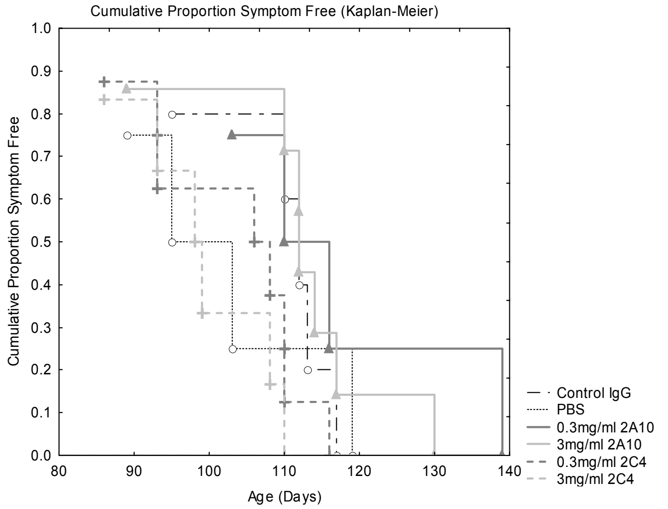

Other Preclinical Pharmacology Studies

GSK1223249 or the mouse parental equivalent mAb have been studied in other in vivo animal models not related to ALS – see Investigators Brochure for further information.

1.2.3. Pre-Clinical Safety

Overview of the Non-Clinical Testing Strategy

GSK1223249 has been engineered to reduce Fc-dependent effector functions, such as antibody-dependent cellular cytotoxicity and complement mediated cell lysis (CML), to minimise the risk of clinical immunogenicity. The rat and monkey were selected for toxicological evaluation of GSK1223249 based on the conservation of the Nogo-A target and because GSK1223249 binds with similar affinity to rat, monkey and human Nogo-A. Anti-Nogo-A mAbs also display pharmacological activity in rodents and primates.

The safety profile of GSK1223249 was evaluated in repeat dose studies in which GSK1223249 was dosed once every 2 weeks in Sprague Dawley rats for up to 12 weeks and cynomolgus monkeys for up to 52 weeks in duration. An enhanced assessment of neurobehavioral function was performed as part of the 12 week repeat dose monkey study. A combined cardiovascular and respiratory safety pharmacology study was also

performed in cynomolgus monkeys using single IV doses of GSK1223249. Female fertility and embryofetal studies have been conducted in Sprague Dawley rats and Dutch Belted rabbits, respectively. An in vitro flow cytometric cross-reactivity study to evaluate binding of GSK1223249 to rat, cynomolgus monkey and human peripheral blood has been conducted. Tissue cross-reactivity studies to evaluate the binding of GSK1223249 to a range of tissues from rat, cynomolgus monkey and human have also been performed.

The binding of GSK1223249 to the central nervous tissue in rabbit has also been assessed.

In vitro Studies

The in vitro flow cytometric cross-reactivity study demonstrated no specific binding of GSK1223249 to cynomolgus monkey, human or rat peripheral blood cells. The in vitro tissue cross-reactivity studies also demonstrated good concordance between human, monkey and rat in terms of the range of tissues in which GSK1223249 specific staining was observed. Importantly, all tissues/histological structures in which specific staining was observed in humans were also positive in the rat and monkey, confirming the relevance of these species for the toxicological evaluation of GSK1223249. Specific, consistent binding to cells with morphology and distribution characteristic of central and peripheral nervous system tissues was observed in all 3 species and in a separate study in rabbit as well. In addition, specific staining was observed in muscular structures within a number of tissues in rat, monkey and human. Some inconsistencies in staining were observed in a few tissues in one or the other animal species and were most likely due to high concentrations of biotinylated GSK1223249 used. This is not considered to be of concern given the lack of adverse findings in the in vivo repeat dose toxicity studies and that there was no unique staining observed in human tissue that was not observed in animals.

In vivo Studies

A comparison of systemic exposure to GSK1223249 achieved in the repeat dose studies in rat and cynomolgus monkey is presented in Table 2.

An assessment of cardiovascular and respiratory safety pharmacology in the cynomolgus monkey showed no effects on ventilatory function, airway resistance, arterial blood pressures, ECG intervals, or any evidence of waveform abnormalities or arrhythmias following single IV (bolus) dosing of GSK1223249 (30 or 300 mg/kg). Mild decreases in mean heart rate (up to 12% from 1 to 3 hours post dose) were observed in the 30 mg/kg treatment group. Decreases in mean heart rate of a similar degree (up to 17%) were also observed in the 300 mg/kg treatment group and persisted for 7 days. The decreases in heart rate are considered mild since there was no difference between the mean heart rate in either treatment group compared with vehicle control, at any time point, and the absolute heart rate values of the treatment groups remained within the range of absolute heart rate values for the vehicle control animals throughout the observation period. Moreover, these effects were not associated with any changes in systolic, diastolic or pulse blood pressures. A dose of 300 mg/kg also produced a mild and transient increase (0.55°C) in body temperature, which was not observed in the repeat dose studies.

In repeat dose toxicity studies, GSK1223249 was administered as an IV (bolus) injection to male and female rats and monkeys at doses of 30, 100 and 300 mg/kg once every 2 weeks for 12 weeks, or 1000 mg/kg once every 2 weeks for 4 weeks (i.e., maximum feasible dose in the rat and limit dose in the monkey) or 20, 100 and 500 mg/kg once every 2 weeks for 52 weeks (monkey only). There was no toxicity or irritancy associated with IV administration of GSK1223249 and the mAb was well tolerated in both species in all studies. One male rat in the low dose group (30 mg/kg) of the 12 week study was

found dead on Day 83. As no test article-related or significant macroscopic or microscopic changes were noted, this death was not considered attributable to test article administration. There were no significant clinical observations or toxicities observed in any repeat dose toxicity study based on assessments of body weight and food consumption, body temperature, ophthalmology, neurobehavioral evaluations (cynomolgus monkey 12 week and 52 week studies only), electrocardiography (cynomolgus monkey only), clinical pathology, organ weights, macroscopic and microscopic observations. There was no evidence of any toxicity on the male and female reproductive organs in either species (including stage-dependent evaluation of spermatogenesis in males). In particular, there were no adverse cardiovascular effects or significant changes in body temperature in the monkey. There was no evidence of immunogenicity present in either species following treatment up to 12 weeks. Development of anti-GSK1223249 antibodies in one animal at 100 mg/kg/dose did not have an impact on study data interpretation because exposure values (AUC) in that animal were within the range of exposure values seen in the other animals, at all sampling times, both prior to and after anti-drug antibodies were detected.

GSK1223249 neutralises and/or antagonises Nogo-A protein which mediates inhibition of neurite outgrowth. Neutralisation of Nogo-A has been shown to promote regeneration in peripheral and central nerves following injury. However, the effect of GSK1223249 on injured or non-injured axons in healthy animals is unknown. Targeted disruption (gene knockout) of Nogo-A, however, in 3 independent mouse models has shown no adverse toxicological effects, and additionally in these knockout models, brain structure and function is normal [Kim, 2003; Simonen, 2003; Zheng, 2003]. Therefore, Nogo-A blockade with anti-Nogo-A antibodies, such as GSK1223249, is unlikely to result in adverse developmental, neurological or behavioural disturbances. Furthermore, in the 12 and 52 week repeat dose monkey toxicity studies, there were no adverse effects in the extensive neurobehavioral functional assessments, reflecting normal peripheral and central nervous system activity. This was supported by the absence of abnormal pathology in wide ranging and in-depth assessments of the peripheral and central nervous systems.

Biopharmaceutical therapeutics, such as mAbs, do not interact directly with DNA or chromosomes. Therefore, genetic toxicology studies were not performed with GSK1223249.

Reproductive toxicology studies were performed with GSK1223249. In a study to evaluate the effects on female fertility and early embryonic development to implantation, female rats were intravenously administered GSK1223249 at 30, 100 or 500 mg/kg/day as a total of 5 consecutive once weekly doses administered on Days 1, 8 and 15 of the pre-mating treatment phase and on Days 6 and 13 post coitum (pc) during the gestation phase. No maternal or developmental toxicity was detected at any dose; therefore, the no observed adverse effect level (NOAEL) was 500 mg/kg/week. The effects of GSK1223249 on pregnancy and embryofetal development were also investigated in female rabbits as Nogo-A is conserved in this species and expressed in the CNS. Following once weekly IV administration at doses of 30, 100 and 500 mg/kg/week (n=22 mated females/group) on Days 7 and 14 pc, systemic exposures (AUC and C_{max}) increased in a dose-proportional manner in the rabbit and were similar between Days 7

and 14 pc. All dose levels were well tolerated and there were no test article-related deaths or clinical signs and there were no effects on maternal body weight gain or food consumption at any dose. There were no test article effects on pregnancy or fetal developmental parameters. Once weekly dosing resulted in sustained systemic exposure to GSK1223249 across the major period of organogenesis. A proportion of rabbits developed anti-GSK1223249 antibodies during treatment (3 of 22 and 2 of 22 in the 30 or 100 mg/kg/week groups, respectively) but were judged not to have compromised the study because the numbers of animals with no detectable anti-GSK1223249 antibodies or altered pharmacokinetics in each group were sufficient to meet all study objectives. The NOAEL for rabbit embryofetal development was 500 mg/kg/week.

Summary

In conclusion, there were no adverse findings seen in 4 week, 12 week (rats and monkeys) or 52 week (monkey only) toxicity studies following administration of 2 doses (given once every 2 weeks) at 1000 mg/kg/dose (i.e., maximum feasible dose in the rat and limit dose in the monkey) and 6 doses (given once every 2 weeks) up to 300 mg/kg/dose, and 26 doses (given once every 2 weeks) up to 500 mg/kg of GSK1223249, respectively. Additionally, there was no evidence of significant neurobehavioral, cardiovascular or respiratory adverse effects following intravenous administration of GSK1223249 in monkeys. In the 4 week toxicity studies, the no observed adverse effect level (NOAEL) of 1000 mg/kg once every 2 weeks represented a mean AUC_{0-t} of 1550 and 1210 mg.h/mL in rats and monkeys, respectively, on Day 1 to Day 15; and 1730 and 1380 mg.h/mL in rats and monkeys, respectively, on Day 15 to Day 29. In the 12 week toxicity studies, the NOAEL of 300 mg/kg once every 2 weeks represented a mean AUC_{0-t} of 592 and 333 mg.h/mL in rats and monkeys, respectively, on Day 1; and 1065 and 405 mg.h/mL in rats and monkeys, respectively, on Day 71. In the 52 week toxicity studies, the NOAEL of 500 mg/kg once every 2 weeks represented a mean AUC_{0-t} of 889.5 and 947 mg.h/mL in monkeys in Weeks 1 and 51, respectively. Therefore, there are no findings from the repeat dose toxicology studies that would preclude single or repeat dosing of GSK1223249 in humans.

Table 2. Mean Plasma Toxicokinetic Parameters for GSK1223249 Following Once Every 2 Week IV (Bolus) Administration in Male and Female Rats, Rabbits and Monkeys

Species (No. of Doses)	Dose (mg/kg)	Sex	C_{max} (mg/mL)		AUC_{0-t} (mg.h/mL)	
			Days 1 to 15	Days 15 to 29	Days 1 to 15	Days 15 to 29
Rat<br>(2 doses)[a]	**1000<br>(NOAEL)**	**M<br>F**	**30.6<br>28.4**	**32.9<br>32.5**	**1570<br>1530**	**1710<br>1750**
Monkey<br>(2 doses)[a]	**1000<br>(NOAEL)**	**M<br>F**	**30.1<br>25.5**	**30.4<br>26.3**	**122<br>1200**	**1480<br>1280**

Species (No. of Doses)	Dose (mg/kg)	Sex	C_{max} (mg/mL)		AUC_{0-t} (mg.h/mL)[f]	
			Day 7	Day 14	Day 7	Day 14
Rabbit<br>(2 doses)[e]	30	F	1.03	1.08	85.2	90.5
	100	F	4.74	5.61	287	283
	**500<br>(NOAEL)**	**F**	**20.3**	**23.0**	**1236**	**1173**

Species (No. of Doses)	Dose (mg/kg)	Sex	C_{max} (mg/mL)			AUC_{0-t} (mg.h/mL)		
			Day 1	Day 43	Day 71	Day 1	Day 43	Day 71[b]
Rat<br>(6 doses)[a]	30[c]	M<br>F	0.932<br>0.885	1.050<br>0.986	1.310<br>1.260	76.4<br>73.5	112<br>105	111<br>108
	100[c]	M<br>F	3.53<br>2.71	3.80<br>3.64	4.46<br>4.26	255<br>206	379<br>344	457<br>416
	**300[d]<br>(NOAEL)**	**M<br>F**	**8.30<br>7.27**	**11.4<br>11.10**	**13.30<br>11.70**	**602<br>582]**	**1050<br>849**	**1120<br>1010**
Monkey<br>(6 doses)[a]	30[c]	M<br>F	0.740<br>0.874	0.952<br>0.869	0.848<br>0.925	26.2<br>29.1	29.0<br>28.1	27.6<br>28.2
	100[c]	M<br>F	2.21<br>2.70	2.49<br>3.21	2.65<br>2.88	108<br>88.9	115<br>101	123<br>97.8
	**300[d]<br>(NOAEL)**	**M<br>F**	**8.45<br>7.29**	**9.01<br>9.61**	**10.1<br>9.55**	**377<br>289**	**433<br>336**	**448<br>363**

Table 2 (Continued). Mean Plasma Toxicokinetic Parameters for GSK1223249 Following Once Every 2 Week IV (Bolus) Administration in Male and Female Rats, Rabbits and Monkeys

Species (No. of Doses)	Dose (mg/kg)	Sex	C_{max} (mg/mL)				AUC_{0-t} (mg.h/mL)			
			Week 1	Week 13	Week 27	Week 51	Week 1	Week 13	Week 27	Week 51
Monkey (26 doses)[a]	20	M<br>F	0.571<br>0.550	0.528<br>0.588	0.631<br>0.648	0.639<br>0.560	25.0<br>18.7	32.8<br>23.6	40.6<br>30.1	42.7<br>26.1
	100	M<br>F	3.11<br>3.30	3.90<br>3.67	2.76<br>2.61	3.01<br>3.28	119<br>109	197<br>194	152<br>124	164<br>165
	**500**	**M**<br>**F**	**19.3**<br>**20.2**	**21.3**<br>**18.1**	**14.2**<br>**15.6**	**14.5**<br>**16.4**	**873**<br>**906**	**1146**<br>**1065**	**923**<br>**858**	**934**<br>**960**

Key: **Bolded** doses are the NOAEL (no observed adverse effect level).
a = Doses were administered once every 2 weeks for 4, 12 or 52 weeks (2, 6 or 26 doses in total).
b = To enable comparison with Day 1 and 43 AUC_{0-t} data, the AUC_{0-t} for Day 71 was calculated using t = 336 hours.
c = n=3/sex.
d = n=6/sex.
e = Once weekly IV administration of GSK1223249 via ear vessel on Days 7 and 14 post coitum
f = AUC_{0-t} was calculated using t = 168 hours.
NOAEL results are reported as mean and [range].

1.2.4. Pre-Clinical Pharmacokinetics and Product Metabolism

Following single intravenous (IV) infusion of GSK1223249 at 1 and 1.5 mg/kg to male Sprague Dawley rats and cynomolgus monkeys, respectively, plasma clearance (CL) and steady state volume of distribution (V_{ss}) were low in both species (CL = <1 mL/min/kg and V_{ss} = ~0.1 L/kg), indicating that GSK1223249 was mainly confined to systemic circulation. The apparent terminal half-life ($t_{½}$) was estimated to be 8 and 4 days (193 and 92.2 hours) in rats and monkeys, respectively. Following single subcutaneous (SC) administration of GSK1223249 at 1 and 1.5 mg/kg to male Sprague Dawley rats and cynomolgus monkeys, respectively, bioavailability was estimated to be 68% (rats) and 100% (monkeys).

Following repeat IV administration of GSK1223249 at doses of 30, 100 and 300 mg/kg (dosed once every other week for 12 weeks for a total of 6 doses) and 1000 mg/kg (dosed once every other week for 4 weeks for a total of 2 doses) in rats and monkeys, systemic exposure to GSK1223249 (C_{max} and AUC_{0-336}) increased generally dose-proportionally. In both rats and monkeys, there were no notable differences in systemic exposure between males and females at any dose or sampling occasion. In rats at 30, 100 and 300 mg/kg, after 6 doses, the systemic exposure was 1.5- and 1.7-fold higher (C_{max} and AUC_{0-336}, respectively) compared to exposure after one dose. In monkeys at 30, 100 and 300 mg/kg, there were no differences in systemic exposure (C_{max} and AUC_{0-336}) after one dose compared to 6 doses. GSK1223249 was detected in the plasma at the end of the 8 week off-dose recovery period in both rats and monkeys. At 1000 mg/kg, although there were no marked changes in systemic exposure (C_{max} and AUC_{0-336}), there was a

trend towards an increase in systemic exposure after the second administration in both rat and monkey.

1.3. Rationale

1.3.1. Study Rationale

FTIH studies are often carried out in healthy volunteers with the primary objective of determining safety and tolerability before going on to studies in the target patient population. The primary purpose of developing GSK1223249 in ALS is to encourage neuronal regeneration/synaptic plasticity at the neuromuscular interface where overexpression of Nogo-A in skeletal muscle appears to be linked to severity of the disease. Overexpression of Nogo-A in ALS skeletal muscle thus offers an opportunity to evaluate the pharmacological effects of GSK1223249 in man – beneficial or otherwise. As with most humanised mAbs with a disabled Fc region, the safety profile of GSK1223249 is most likely to be linked to effects on its target, Nogo-A. Healthy subjects are not an appropriate human model to evaluate the safety of GSK1223249 because the target is not appreciably expressed in their skeletal muscle. Although Nogo-A is expressed in the CNS, it is not significantly exposed in the absence of CNS disease or injury. Thus, potential CNS effects may not even be detectable in healthy subjects. Therefore, evaluation of GSK1223249 in healthy volunteers is unlikely to discharge potential peripheral or central nervous system risks. Hence, the population that will be studied in this FTIM protocol is ALS patients.

In terms of potential benefit for individual patients, those participating in Part 1 will receive a single dose and are highly unlikely to derive any significant benefit from the treatment. Those patients participating in Part 2 of the study will receive 2 doses which, in view of its long half life, may have a long duration of action to 3-4 months. It is thus possible, that some patients in the active groups in Part 2 may experience some beneficial effect. However, in view of the short period of assessment and the small numbers of patients, no real improvements in the ALSFRS-R scores are expected in this first clinical trial, which is essentially a safety study.

The design of this study is a two-part, fusion protocol design. Part 1 of the protocol will consist of evaluating single dose safety and pharmacokinetics, and Part 2 will determine repeat dose safety and pharmacokinetics. Both parts will investigate pharmacodynamic biomarkers. The rationale behind this design is to expedite clinical development in this condition of very high unmet medical need. Each patient will only be allowed to participate in one part of the study, i.e. patients who participate in Part 1 will not be allowed to participate in Part 2. The repeat doses in Part 2 will be administered approximately 4 weeks apart. As the half-life of most IgG1-type mAbs is about 3 weeks, an interval of 4 weeks between the two repeat doses in Part 2 represents a reasonable dosing interval to test for any potential immunogenicity.

In two cohorts in Part 1 and all cohorts in Part 2, blood samples and skeletal muscle biopsies will be taken from patients before and at the end of treatment to demonstrate whether or not GSK1223249 binds to its target and produces any measurable pharmacodynamic effects (See Section 2). This is an important objective of this study

which is expected to provide the rationale for continued development of GSK1223249 in ALS, if the safety and PK profiles support the same.

1.3.2. Dose Rationale

Prediction of Human Pharmacokinetics

Human pharmacokinetic studies show that IgG antibodies following IV administration, typically have a biphasic disposition, with a total volume of distribution of 3-8 L and a clearance range of 9-265 mL/hr, leading to a half-life range of 0.3 –30 days [Jusko, 2005], with the majority in the range of 21-28 days [Lobo, 2005].

Clearance of antibodies is driven *via* catabolism by proteases, located in cells such as vascular endothelial cells or muscle cells. Furthermore, intact antibody is unlikely to be excreted *via* the kidneys. As such, the incorporation of physiological variables other than body weight, such as liver blood flow, is generally considered not to improve the prediction of human pharmacokinetics. However, the degree of humanization can affect the disposition (both clearance and volume of distribution) of these compounds, which may vary between species even after body weight correction. For humanized IgG antibodies, the species thought to be most predictive of human disposition is the cynomolgous monkey. Human pharmacokinetics of GSK1223249 was therefore predicted by allometric scaling using data from a cynomolgus monkey single dose (1.5 mg/kg) pharmacokinetic study [GlaxoSmithKline Document Number GSK07DMF020].

The clearance of GSK1223249 in humans was predicted with the following equation:

$$CL_{humans}(mL/hr/kg) = CL_{monkeys}(mL/hr/kg) \times \left(\frac{\text{Body weight}_{humans}}{\text{Body weight}_{monkeys}}\right)^{-0.25}$$

Given that the mean clearance in monkeys was estimated to be 0.946 mL/hr/kg, the predicted human clearance for GSK1223249 was 0.451 mL/hr/kg, using a 70 kg human being and a 3.6 kg cynomolgus monkey. This predicted human clearance is about 2 fold higher than the conventional estimate of 0.208 mL/hr/kg intrinsic clearance, characteristic of the FcRn receptor cycling pathway [Tabrizi, 2006] and observed with many mAbs in clinical use.

The volume of distribution of the central compartment (Vc) for GSK1223249 in cynomolgus monkeys was 88.8 mL/kg. It has been shown that the volume of distribution values expressed in mL/kg for cynomolgus monkeys and humans are similar [Mordenti, 1991].

The terminal half life (T1/2) of GSK1223249 in humans was predicted with the following equation:

$$T1/2_{humans}(hr) \cong T1/2_{monkeys}(hr) \times \left(\frac{\text{Body weight}_{Monkeys}}{\text{Body weight}_{Humans}}\right)^{-0.25}$$

Given that the mean GSK1223249 T1/2 in monkeys was estimated to be 92.2 hr (3.84 days), the predicted human T1/2 for GSK1223249 was 193 hr (8.0 days) ranging [4.4 – 12.8 days] for a 70 kg human being and a 3.6 kg cynomolgus monkey. This predicted human T1/2 for GSK1223249 is about 3 fold lower than the conventional estimate of 21 days, characteristic of the FcRn receptor cycling pathway [Tabrizi, 2006] and observed with many mAbs in clinical use.

Selection of starting dose based on MABEL

The starting dose for this clinical trial was selected to generate a predicted maximum plasma concentration (Cmax) that is below the Minimum Anticipated Biological Effect Level (MABEL). The MABEL was determined from the neutralizing effect of GSK1223249 on Nogo-A *in vitro* using human recombinant GST-Nogo-A5+6 (containing the inhibitory activity of full-length Nogo-A) and rat post-natal (day 8) primary cerebellar granule neurons. In this assay, the MABEL value was estimated to be 4.0 µg/mL and the IC_{50} value for the neutralizing effects of GSK1223249 was 75 nM (11.3 µg/mL). A MABEL based on the effect of GSK1223249 *in vivo* has not been determined. Hence a conservative safety factor (x40) has been applied to the *in vitro* MABEL in selecting the starting dose, using the following equation:

$$\text{Starting dose} = \frac{\text{MABEL}}{\text{Safety factor}} \times Vc_{\text{humans}} = 0.01\,\text{mg/kg}$$

where, MABEL = 4 µg/mL, Safety factor (selected) = 40, Vc_{humans} = 88.8 mL/kg

The following assumptions for the calculation of the starting dose were made:

- The neurite outgrowth experiment is a good surrogate for the in vivo effects of GSK1223249
- The GSK1223249 concentrations required to elicit a response in vivo and in vitro are identical
- The starting dose will produce a Cmax in plasma = MABEL/40

In addition to the MABEL, another consideration in selecting the starting dose was the predicted percentage binding to the target Nogo-A as shown below.

Prediction of target binding at Tmax

The predicted fraction of Nogo-A bound to GSK1223249 in peripheral nerve was calculated with the following equation:

$$\%\frac{B}{B\max} = 100 \times \frac{C_{PN}/KD}{1 + C_{PN}/KD}$$

where B is the fraction of Nogo-A bound to GSK1223249, Bmax is the maximum number of Nogo-A binding sites, KD is GSK1223249 affinity for the recombinant orthologues of human Nogo-A (kD = 0.096 µg/mL), C_{PN} is the concentration of

GSK1223249 in peripheral nerve at Tmax in plasma (calculated with a plasma/peripheral nerve concentration ratio value of 3, as determined with an IgG1mAb with a similar structure). It was also assumed that GSK1223249 behaves as an antagonist/inhibitor.

Figure 6 represents the predicted fraction of Nogo-A bound to GSK1223249 at Tmax (plasma) following administration of various doses.

The predicted fraction of Nogo-A bound to GSK1223249 in peripheral nerve at Tmax of the starting dose of 0.01 mg/kg is 28.1% , compared to 97.7% for the MABEL (4 µg/mL) and 99.2% for the IC_{50} (11.3 µg/mL) of the *in vitro* neurite outgrowth experiment.

Figure 6 Predicted fraction of Nogo-A bound to GSK1223249 at Tmax (plasma)

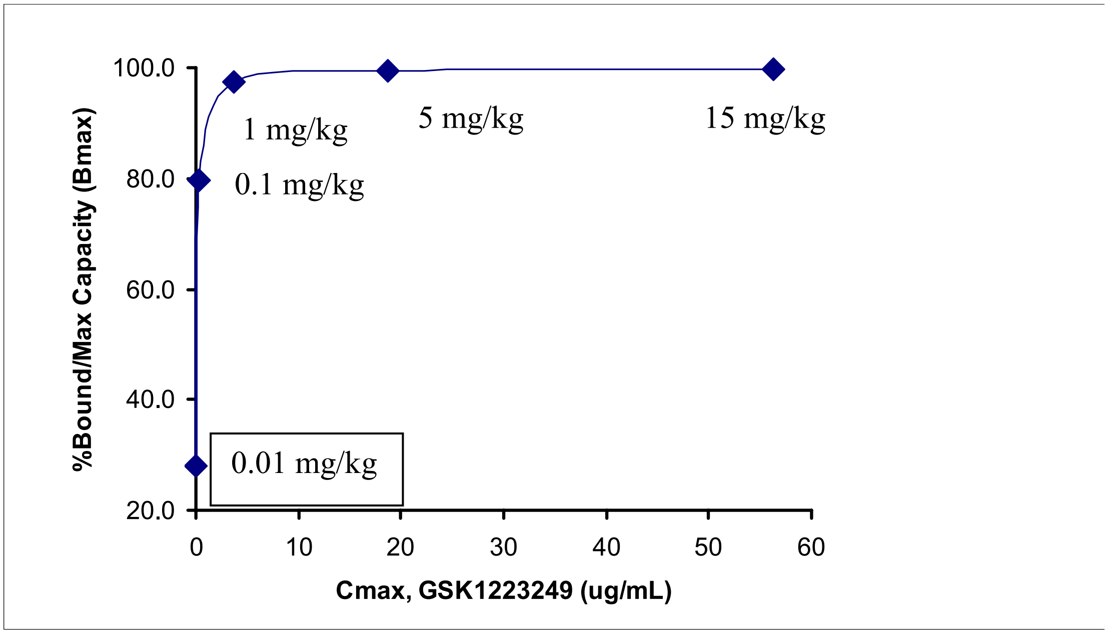

Based on the above, the proposed starting dose of 0.01 mg/kg is predicted to give approximately 28 % binding to Nogo-A which is very low for an antagonist-type pharmacological action.

Emerging clinical data

Emerging clinical data from Part 1 of this protocol, in which a total of 40 subjects were dosed at increasing single dose levels including 15mg/kg, suggests that this dose is tolerated well in patients with ALS. Thus, an increase in the top repeat dose in Part 2 to two doses of 15 mg/kg given 28 days apart is deemed to be in line with a safe and cautious approach to fully characterize GSK1223249 and its effects in the ALS patient population in this early phase of clinical development.

The emerging pharmacokinetics data in ALS subjects from Part 1 indicated that GSK1223249 plasma concentrations in ALS subjects were within 2 to 3 fold higher than the predictions based on allometric scaling of monkey data. The PK parameters

calculated from these PK observations are as follows: a central compartment Vc of ~ 40 mL/kg, a human clearance of ~ 0.14 mL/h/kg and a half life of ~20 days.

Based on these updated PK data from Part 1, the Cmax and AUC values predicted in humans were refined (see Table 3 and Table 4)

Selection of top dose based on safety cover

GSK1223249 was well tolerated in the cynomolgus monkey following fortnightly (one dose every two weeks) IV bolus administration for 52 weeks, at doses of 20, 100 and 500 mg/kg. The no observed adverse effect level (NOAEL) was 500 mg/kg (the highest dose examined). The systemic exposure (as defined by $AUC_{0\text{-}2\ weeks}$ and C_{max}) was similar between male and female monkeys. The mean systemic exposure (males and females combined) of GSK1223249 at 500 mg/kg for the first dose was $AUC_{0\text{-}2\ weeks}$ of 890 mg.h/mL and C_{max} of 19.8 mg/mL; and for the last dose was $AUC_{0\text{-}\tau}$ of 947 mg.h/mL and C_{max} of 15.4 mg/mL.

The NOAEL $AUC_{0\text{-}2\ weeks}$ of 890 mg.h/mL (**= 890000 µg.h/mL**) and C_{max} of 15.4 mg/mL **(= 15400 µg/mL**) have been conservatively selected for the calculation of safety covers for planned doses of this study, as shown in Table 3 and Table 4. Because of differences in frequency in dosing the monkeys, $AUC_{0\text{-}2\ weeks}$ represents a conservative estimate over the human cover represented by **by $AUC_{0\text{-}inf}$ after a single dose (Table 3) and** $AUC_{0\text{-}4\ weeks}$ after 2 GSK1223249 doses, 4 weeks apart (Table 4).

Two doses of 15 mg/kg separated by 28 days is the planned maximum dosing regimen that will be explored in this study. The corresponding safety margins are 33-fold based on dose, 10-fold based on $AUC_{0\text{-}\tau}$ and 35-fold based on C_{max}

Table 3 Single Dose Part 1: Estimated safety cover of planned single doses, in humans (part 1), using a NOAEL in monkeys of 500 mg/kg once every 2 weeks for 52 weeks.

Proposed clinical dose of GSK1223249	Estimated human exposure based on last updated clinical data from NOG111330		Predicted exposure cover in humans		
**Dose (mg/kg)**	AUC_{0-inf} (μgxh/mL)	Cmax (μg/mL)	**Based on dose ratio[a]**	**Based on AUC ratio[b]**	**Based C_{max} ratio[c]**
0.01	71.4	0.3	50000 x	12465 x	51333 x
0.1	714.3	2.6	5000 x	1246 x	5133 x
1	7142.8	26.3	500 x	124.6 x	513 x
5	35714.3	131.5	100 x	25 x	117 x
15	107149.0	394.4	33 x	8 x	39 x

a. Monkey NOAEL / human dose (mg/kg)
b. Monkey NOAEL $AUC_{0-2weeks}$ / human AUC_{0-inf}
c. Monkey NOAEL Cmax/ human Cmax

Table 4 2nd Dose of the Two Doses Separated by 28 days, Part 2: Predicted safety cover of planned 2nd dose in humans (Part 2), using a NOAEL in monkeys of 500 mg/kg once every 2 weeks for 52 weeks.

Proposed clinical 2nd dose of GSK1223249	Estimated human exposure based on last updated clinical data from NOG111330		Predicted exposure cover in humans		
**Dose (mg/kg)**	AUC_{0-T} (μgxh/mL)	Cmax (μg/mL)	**Based on dose ratio[a]**	**Based on AUC ratio[b]**	**Based C_{max} ratio[c]**
0.5	3070	14.7	1000 x	290 x	1048 x
2.5	16915	73.3	200 x	53 x	210 x
15	92096	440.1	33 x	10 x	35 x

AUC_{0-T} represent the AUC from 0 to 4 weeks
a. Monkey NOAEL / human 2nd dose (mg/kg)
b. Monkey NOAEL $AUC_{0-2weeks}$ / human $AUC_{0-4weeks}$
c. Monkey NOAEL Cmax/ human Cmax

1.4. Summary of Risk Management

Apart from general risks associated with clinical trials in ALS, this section highlights any special risks that may be foreseen in this trial and with administration of GSK1223249.

As mentioned in Section 1.2.2, GSK1223249 is an IgG1-type humanised monoclonal antibody with a ‘disabled’ Fc region which substantially reduces or abolishes complement C1q binding. This implies little or no likelihood of toxicity mediated by Fc-effector function e.g. complement dependent cytotoxicity and antibody mediated cellular

toxicity reactions. Additionally, the binding of GSK1223249 to Nogo-A is highly specific and as with most mAbs that antagonise or neutralise target proteins, any safety problems are more likely to be target-specific (i.e., exaggerated pharmacology). As the target (Nogo-A) is not on immune cells, it is unlikely that GSK1223249 will have an adverse effect on the immune system.

One consideration of theoretical concern is whether or not neutralisation of Nogo-A in patients with ALS may have an unforeseen adverse effect. As noted in the preclinical safety assessment, no significant adverse effects (target or non-target related) were observed in the 3 month toxicology studies in two species even at the highest tested doses of 300 mg/kg/day. Furthermore, in SOD1 mutant transgenic mice, there was no evidence of a deleterious effect of GSK1223249 compared with control. A clinical trial of an anti-Nogo-A antibody (ATI355) in patients with spinal cord injury is currently ongoing (see [ID No.NCT00406016] at www.clinicaltrials.gov) and no safety issues have been reported to date. **Therefore there is precedented human exposure to an anti-Nogo-A agent**. Nevertheless, comprehensive safety monitoring is built into the protocol, including full medical and neurological assessments, electrophysiological assessment (MUNE), clinical safety labs and electrocardiograms (ECGs). Should there be any safety signal detected, additional diagnostic tests (e.g. MRI) may be ordered by the investigator as clinically needed. In accordance with current guidelines for FTIH studies, all doses will be administered whilst the patient is in a hospital-based clinical unit appropriately equipped and staffed to handle medical emergencies. Patients will remain in the unit for observation for at least 24 hours after the first dose (and 6 hours after the second dose) before being discharged for out-patient follow-up. In accordance with current FTIH study guidelines, staggering of initial doses has been implemented in the study design.

Both parts of the study include a muscle biopsy at two timepoints. In many centers, patients with ALS undergo an open skeletal muscle biopsy as part of their usual diagnostic work-up. There is a slight risk of bleeding and infection from such procedures, thus, patients in both parts will be clearly informed about these risks before giving consent participate in the study. Additionally entry criteria will exclude patients who are likely to experience prolonged bleeding after the muscle biopsy procedure.

ALS is a serious, life-threatening disease and since no known significant adverse events have been observed in the pre-clinical studies with GSK1223249, it seems reasonable to embark on a clinical trial program of this highly specific, humanised, monoclonal antibody in patients with ALS.

Overdosage: There is no specific antidote for overdose with GSK1223249. In the event of a suspected overdose, it is recommended that the appropriate supportive clinical care should be instituted (including plasmapheresis), as dictated by the subjects' clinical status.

2. OBJECTIVE(S) AND ENDPOINTS

Table 5 Objectives and Endpoints of Study

	PRIMARY OBJECTIVE	ENDPOINTS	NOTES
1	To evaluate the safety and tolerability of single and repeat i.v. doses of GSK1223249 in patients with ALS	Adverse events, vital signs, standard physical examination, ECG and standard clinical laboratory tests (haematology and biochemistry).	Evaluation of safety signals will include any adverse effects on Immunogenicity, ALSFRS-R, SVC, Muscle Strength and MUNE.
	**SECONDARY OBJECTIVES**	**ENDPOINTS**	**NOTES**
2.1	To evaluate the plasma pharmacokinetics (PK) of single and repeat i.v. doses of GSK1223249 in patients with ALS.	GSK1223249: Single dose PK (Part 1): AUC(0-∞), AUC(0-t), AUC(0-Week 4), %AUCex, Cmax, tlast, CL, Vss, MRT, λz and t½.Repeat dose PK (Part 2): GSK1223249 AUC(0-Week 4), Cmax and after the second dose, AUC(0-t), AUC(0-∞),λz and t½.	
2.2	To evaluate immunogenicity of single and repeat i.v. doses of GSK1223249	Presence of antibodies to GSK1223249 will be assessed in serum samples using immuno-electrochemiluminescent assay.	
2.3	To evaluate the effect of GSK1223249 on clinical endpoints associated with ALS	ALSFRS-R scores, Manual Muscle Strength test and Slow Inspirational Vital Capacity (SVC)	
2.4	To evaluate the effect of GSK1223249 on electrophysiological endpoints associated with ALS	Motor Unit Number Estimation (MUNE)	
2.5	To evaluate the effects of GSK1223249 on a variety of pharmacodynamic biomarkers	Muscle biopsies for protein analysis, Immunohistochemistry (IHC), Transcriptomics. Blood for protein analysis and other biomarkers.	
2.6	To evaluate PK-PD relationships	Relationships between PK of GSK1223249 and relevant pharmacological endpoints	As data permit.

3. INVESTIGATIONAL PLAN

3.1. Study Design/Schematic

Table 6 Schematic of FTIH Design

2-Part Study: Randomised Placebo-Controlled Double-Blind Sequential Repeat Dose Escalation Design																
**Part 1: Single Dose Escalation (N ~ 40). Doses in mg/kg i.v. A = Active, P = Placebo. Open muscle biopsy (◆) before and after treatment in Cohort 3 (1mg/kg), are voluntary collections, but in Cohort 5 (15mg/kg) they are required collections ('low and high dose muscle biopsy cohorts').**																
Cohort 1 (2P, 6A)	0.01															
Cohort 2 (2P, 6A)		0.1														
Cohort 3 (2P, 6A)			◆	1	◆											
Cohort 4 (2P, 6A)						5										
Cohort 5 (2P, 6A)							◆	15	◆							
**Part 2: Repeat Dose Escalation – open muscle biopsy (◆) before and after treatment (n~ 36)**																
Cohort 6 (3P, 9A)							◆	0.5	0.5	◆						
Cohort 7 (3P, 9A)										◆	2.5	2.5	◆			
Cohort 8 (3P, 9A)													◆	15	◆	15

3.2. Discussion of Design

This study will be a randomized, placebo-controlled, double-blind, sequential dose escalation, 2-part fusion protocol. A sufficient number of subjects will be enrolled into the study such that approximately 76 subjects complete dosing and critical assessments. Each subject will only be allowed to participate in one part of the study, i.e. subjects who participate in Part 1 will not be allowed to participate in Part 2. This study will be conducted in multiple sites.

In both Part 1 and 2, a dose escalation committee (DEC), consisting of GSK staff plus an external ALS neurologist, will review key safety data before recommending escalation to the next dosing cohort. Should the DEC require unblinding, the core GSK study team staff will remain blinded and remove themselves from the review. The composition of the DEC and description of those who will remain blinded is described more fully in Section 10.1

In Part 1, single escalating intravenous (i.v.) doses of GSK1223249 will be evaluated in sequential cohorts consisting of 2 placebo and 6 active treated subjects per cohort to determine single dose safety and pharmacokinetics (PK). In addition, open skeletal muscle biopsies, which are voluntary collections in Cohort 3 (1mg/kg) and required

collections in Cohort 5 (15mg/kg) are to be collected from patients at pre-dose and at week 4 to demonstrate whether or not GSK1223249 binds to its target and produces any measurable pharmacodynamic (PD) effect. Blood samples at pre-dose and at week 4 will be collected regardless of whether or not a muscle biopsy will be taken.

The planned cohorts are shown in Table 6 as Cohorts 1 – 5. After written informed consent and successful screening within 28 days of Day 1, subjects will be admitted into a hospital-based clinical unit for dosing and observation. It is recommended that subjects be admitted into the hospital-based unit the day before dosing for some pre-dosing procedures. However, where necessary, subjects may be admitted on the dosing day and pre-dosing procedures completed on same day before dosing. Following completion of pre-dosing procedures, each subject will receive a short infusion and remain under observation for at least 24 hours post-dose before being discharged at the investigator's discretion and be followed up on an out-patient basis. Each subject will be followed-up for at least 12 weeks as shown in the Time &Events (T&E) Table (Table 8). The number and schedule of the final follow-up visit after Week 12 for each subject will vary depending on plasma concentrations of GSK1223249 reaching a low enough level to allow a final blood sample to be taken for immunogenicity assays. Subject will then be fully discharged from the study.

Dose staggering in Part 1: In each cohort in Part 1, the first four subjects will be dosed in a staggered manner such that only one will receive the dose in any 24 hours. This dose-staggering will allow investigators to ensure that there are no acute safety issues before the next subject is dosed. This process will be coordinated via the randomization process and by the sponsor's study monitors. Dosing of other subjects in Part 1 will **not** be staggered unless emerging safety signals suggest otherwise.

Part 2 will also be of a sequential dose escalating design, with subjects in each of the planned 3 cohorts (3 placebo, 9 active in each cohort) receiving 2 repeat i.v. doses separated by approximately 4 weeks. The planned repeat doses in Part 2 are dependent on demonstration of the safety of equivalent or higher doses in Part 1 (see Section 3.3.1 for data required for dose escalation decisions and Section 3.3.4 for criteria for progression from Part 1 to Part 2). In Part 2, safety and PK will be evaluated.

- In **cohorts 6 and 7** open skeletal muscle biopsies and biomarker blood samples will be collected. Muscle biopsies and blood sample will be collected from patients at pre-dose and at post dose Week 8.
- In **cohort 8** open skeletal muscle biopsies and biomarker blood samples will be collected. Muscle biopsies and blood sample will be collected from patients at the pre-dose visit and at one timepoint after the first dose, either Day 1(+24hr), Day 8 or Week 4 (Day 22-28): subjects will assigned to a timepoint based on subject preference determined at screening (see Section 6.6.1). If the subject has the Day (1+24hr) collection then the pre-dose muscle biopsy and blood sample must be collected at least 8 days before Day 1.

(All post dose muscle biopsies collections should be as above unless emerging data suggests these should be collected at an alternative week).

The purpose of collecting muscle biopsies and biomarker blood samples is to demonstrate whether or not GSK1223249 binds to its target and produces any measurable pharmacodynamic (PD) effect.

In Part 2, after written informed consent and successful screening within 28 days of Day 1, subjects will be admitted into a hospital-based clinical unit for dosing and observation. It is recommended that subjects be admitted into the hospital-based unit the day before dosing for some pre-dosing procedures. However, where necessary, subjects may be admitted on the dosing day and pre-dosing procedures completed on the same day before dosing. Following completion of pre-dosing procedures, subjects will receive their first i.v. infusion and remain under observation for at least 24 hours post-dose before being discharged at the investigator's discretion to be followed up on an out-patient basis as shown in the T&E Table (Table 9). After four weeks, provided there are no contra-indications to continuing treatment, subjects will return to the clinical unit for their second dose. Again, it is recommended that subjects be admitted into the hospital-based unit the day before dosing for some pre-dosing procedures. However, where necessary, subjects may be admitted on the dosing day and pre-dosing procedures completed on the same day before dosing. Following completion of pre-dosing procedures, subjects will receive their second i.v. infusion. Following the second i.v. infusion, subjects will remain in the clinical unit and be observed for at least 6 hours post-dose before being discharged at the investigator's discretion to be followed up on an out-patient basis as shown in the T&E Table (Table 9). After their second dose, each subject will be followed-up for at least 16 weeks as shown in the T&E Table (Table 9). The number and schedule of the final follow-up visit after week 16 for each subject will vary depending on plasma concentrations of GSK1223249 reaching a low enough level to allow a final blood sample to be taken for immunogenicity assays. Subject will then be fully discharged from the study.

Open muscle biopsies in Parts 1 and 2: The muscle biopsies will be taken from the weaker deltoid muscle. The strength of the muscle from which the biopsy is taken should be Grade 3 or 4 in the Medical Research Council (MRC) scale. Patients with wasted deltoids (MRC ≤ 2) will be excluded from the study. Both biopsies should be taken from the same deltoid muscle if at all possible. If not possible, for medical reasons, then the second biopsy can be taken from the opposite deltoid muscle provided the muscle strength is Grade 3 or 4 on the MRC. If the opposite deltoid muscle does not fulfill the MRC criteria, then consideration may be given to taking the second biopsy from another suitable skeletal muscle.

Dose staggering in Part 2: In the first cohort in Part 2, the first four subjects will be dosed in a staggered manner such that only one will receive the dose in any 24 hours. This applies to both Dose 1 and Dose 2. This dose-staggering will allow investigators to ensure that there are no acute safety issues before the next subject is dosed. This process will be coordinated via the randomization process and by the sponsor's study monitors. Dosing of other subjects in Part 2 will **not** be staggered unless emerging safety signals suggest otherwise.

In both Parts 1 and 2, the timing of safety and PD assessments and PK blood sampling may be altered during the course of the study based on emerging data to better investigate safety, PD or PK signals. If the profile indicates that more sampling or assessments are needed, additional visits may be scheduled. If for reasons of safety, dose escalation to an unplanned lower dose is recommended, it will imply that additional cohort(s) may be enrolled to allow for evaluation of the additional dose level(s).

3.3. Dose Adjustment/Stopping Criteria (Parts 1 and 2)

3.3.1. Planned Doses and Data Required for Dose Escalation

Table 6 shows the planned doses and escalation steps. Once the last patient in each dosing cohort has been enrolled, enrolment will be suspended until the DEC has met to review the key safety data from the present cohort and recommend escalation to the next dosing cohort.

Based on careful evaluation of emerging cumulative data (safety, PK and other data), escalation may be to a lower dose than planned, but escalation will not be to a higher dose than planned. The decision to escalate to the next dose will be made by the DEC based on evaluation of cumulative data (focusing on safety and PK) from the current dose (and preceding doses, where applicable). **In Part 1**, cumulative data (focusing on safety and PK) collected from all subjects up to and including the Day 8 Visit will be evaluated blinded and non-Quality Controlled (QC'ed) for the dose-escalation decision. Considering the time it will take to collect this data and have a thorough evaluation by the DEC, it is estimated that there will be approximately 4 weeks between dosing of the last patient in the current cohort and dosing of the first patient in the next cohort. **In Part 2**, cumulative data (focusing on safety and PK) collected from at least six subjects up to and including the Day 36-42 Visit will be evaluated (blinded and non-QC'ed) for the dose-escalation decision.

In both Parts 1 and 2, the main data for the dose escalation decision will be cumulative safety and tolerability data from preceding cohorts. Safety consideration will include any obvious trends in deterioration of Slow inspirational Vital Capacity (SVC), Manual Muscle Testing (MMT), ALSFRS-R and MUNE, where applicable. Escalation will not be stopped based solely on PK grounds alone, unless the predicted AUC for the next dose may exceed the safety cover associated with the NOAEL in primates (see Table 3 and Table 4). PK considerations may thus influence a decision to escalate to a lower dose than planned.

3.3.2. Stopping Criteria for Individual Subjects in Part 2

General Stopping Criteria

In Part 2, if a subject has received the first dose (Dose 1), there is no provision for him/her to receive a different dose size (e.g. a reduced dose) as second dose (Dose 2). Based on medical evaluation of the emerging safety data from each subject, the Investigator will decide whether or not the subject can receive the second dose. Specific liver chemistry and QTc criteria for not proceeding to the second dose in an individual subject are described below. Additionally, the decision not to proceed to the second dose will be based on the Investigator's medical judgment or upon the subject's request. In particular, Investigators must pay careful attention to any clinically significant deterioration in the patient's condition as judged by physical examination, SVC, MMT, ALSFRS-R and MUNE endpoints prior to Dose 2. In case the investigator has any doubt about further dosing with investigational product they should consult with the GSK medical monitor before making a final decision. Even if a subject does not receive Dose 2 for any reason, every effort should be made to follow the subject up for 16 weeks, if at all possible.

Liver Chemistry Stopping Criteria

Liver chemistry threshold stopping criteria have been designed to assure subject safety and to evaluate liver event etiology during administration of investigational product and the follow-up period. Initial administration of investigational product (i.e. Dose 1) or further dosing with the investigational product (i.e. Dose 2) will be stopped if any of the following liver chemistry stopping criteria is met:

- ALT ≥ 3xULN and bilirubin ≥ 2xULN (>35% direct): this is considered an SAE and must be handled as described in Section 11.
- ALT ≥ 5xULN.
- ALT ≥ 3xULN if associated with appearance or worsening of signs or symptoms of hepatitis or hypersensitivity (fatigue, nausea, vomiting, right upper quadrant abdominal pain or tenderness, fever, rash, or eosinophilia).

Subjects with ALT ≥ 3xULN **and** < 5xULN **and** bilirubin < 2xULN, who do not exhibit hepatitis symptoms or rash, can continue investigational product as long as they can be monitored weekly for up to 4 weeks. See Section 12 (Liver Chemistry Testing Procedures), for details on weekly follow-up procedures.

QTc Withdrawal Criteria

A subject that meets either of the criteria below will have the investigational product stopped.

- QTcB > 500 msec or uncorrected QT >600msec (machine or manual over-read)
- If subject has bundle branch block then criteria is QTcB > 530 msec

These criteria are based on an average QTc value of triplicate ECGs. If an ECG demonstrates a prolonged QT interval, obtain 2 more ECGs over a brief period, and then use the averaged QTc values of the 3 ECGs to determine whether the subject should be discontinued from the study.

3.3.3. Criteria for Suspension/Dose Adjustment/Stopping Study

Note that these criteria apply to both Parts 1 and 2 of the study. The DEC will invoke suspension of study dosing if clinically significant safety findings related to study drug are observed during ongoing monitoring of safety data or during review of safety data at the end of a cohort. Following such suspension of dosing, all of the cumulative safety data will be reviewed by the DEC **and** the GSK Global Safety Board (GSB) before a decision will be taken to either continue dosing in the study or terminate the study. GSB is an internal GSK independent panel of senior clinicians with drug safety expertise and final accountability for medical governance for GSK clinical studies.

3.3.4. Criteria for Progression from Part 1 to Part 2

The decision for the study to proceed to Part 2 will be taken by the DEC after the interim analysis at the end of cohort 4. Provided there are no safety concerns cohort 5 in part 1 will run in parallel with cohort 6 in part 2. Focusing on safety and PK, cumulative blinded and non-Quality Controlled (QC'ed) data will be analysed from all subjects up to and including the Day 8 Visit, at a minimum, prior to proceeding between cohorts. Considering the time it will take to collect this data and have a thorough evaluation by the DEC, there will be an appropriate period between dosing of the last patient in cohort 4 and dosing of the first patients in cohort 5 and cohort 6. The planned total dose in cohort 6 is dependent on demonstration of the safety of equivalent or higher single doses in Part 1 as shown in Table 6. In addition cohort 7 will not begin until the data collected from all

subjects (as a minimum up to and including the Day 8 Visit) from cohort 5 have also been evaluated. A blinded summary of the safety data from the single doses administered in cohorts 1 to 4 of Part 1 will be sent to the Chair of the GSK Global Safety Board for information. If required, this blinded data summary may also be sent to appropriate Regulatory Authorities, IRBs or IECs, for their information along with the decision to proceed. However, it is proposed that the study will proceed to Part 2 **without the need for further approval** from such bodies.

As described in Section 3.3.3, if clinically significant safety findings related to study drug are observed in Part 1, the study will be suspended and all of the cumulative safety data will be reviewed by the DEC **and** the GSK Global Safety Board before a decision will be taken to either continue dosing in the study or terminate the study. If the decision is to continue, then dosing in Part 1 will need to be completed and the safety data evaluated by both bodies before dosing in Part 2 can be recommended to start.

Dosing in Part 2 will also be suspended as described in Section 3.3.3, if clinically significant safety findings related to study drug are observed.

3.4. Replacement of subjects

If a subject withdraws consent or is withdrawn by the investigator from the study, the subject will not be replaced (i.e., the subject number for the withdrawn subject will not be reused by another subject). However, if necessary, in order to better characterize the safety and tolerability or pharmacokinetics/pharmacodynamics of a given dose, the numbers of subjects per cohort may be increased to 4 additional subjects.

3.5. Treatment Assignment

Subjects in each cohort will be centrally randomized across all sites via an Interactive Voice Response System (IVRS). Randomization constitutes enrollment into the study. The randomization schedule will be computer generated using the Randall system by Discovery Biometrics, GSK. In addition, subjects in cohort 8 will be assigned to a post-dose timepoint for the muscle biopsy sample based on subject preference determined at screening (see Section 6.6.1).

The first 4 subjects in all cohorts of Part 1 and the first cohort of Part 2 will be randomized and **dosed in a staggered manner**, (i.e., only one subject, whether active or placebo, will be randomized and dosed within any 24hr period). This staggered entry into the study will be coordinated via the randomization process and by the sponsor's study monitors. In the case of the first cohort in Part 2, this staggering applies to both Dose 1 and Dose 2.

3.6. Description of Investigational Product

The investigational product that will be used is a clear to opalescent, pale brown to brown liquid solution of GSK1223249 (200mg per mL, in 50mM acetate buffer, pH 5.5 containing 0.02% polysorbate 80 and made isotonic with sodium chloride). One mL filled under nitrogen into type I, clear, glass vials, nominal size 2mL, closed with Helvoet Omniflex Plus stoppers and over- sealed with aluminium collars with tear-out tab. This is detailed in Table 7. Placebo for this study will be sodium chloride and will be sourced by site. Masking of the infusion lines and giving sets will be required to maintain study blind. Details of the dilution, preparation and administration of the infusion can be found in the SPM.

Table 7 Formulation details of GSK1223249 and Placebo

	**Investigational Product**	
**Product Name**	**GSK1223249**	**Placebo**
Formulation description	200mg/ml solution consisting of 200.0 mg GSK1223249, 6.81mg Sodium acetate trihydrate, 0.20mg Polysorbate 80, 6.10mg Sodium chloride, q.s. to pH 5.5 Hydrochloric acid, to 1.0mL[a] water for injections and nitrogen to fill headspace.	0.9% Sodium chloride Ph. Eur/USP
Dosage form	IV infusion	IV infusion
Unit dose strength Dosage levels	0.01 mg/kg, 0.1 mg/kg, 0.5 mg/kg, 1mg/kg, 2.5 mg/kg, 5 mg/kg, 7.5 mg/kg, 15 mg/kg	NA
Route/Administration/ Duration	Slow IV infusion over 60 minutes, except for the 0.01 mg/kg dose which should be infused over 11.2 mins.	Slow IV infusion over 60 minutes, except for the 0.01 mg/kg dose which should be infused over 11.2 mins.
Physical description	Clear to opalescent, pale brown to brown liquid	Clear to colourless saline solution
Device	The infusion will be delivered by a programmable infusion/injection pump	The infusion will be delivered by a programmable infusion/injection pump
Manufacturer or Source of procurement	GSK	Sourced by sites
Dosing instructions	For dosing instructions please refer to the SPM	For dosing instructions please refer to the SPM

a. The solution density is 1.035g/mL (SG).

Table 8 Time and Events Table for Subjects in Part 1

Procedure[11]	Screening	Day-1/or Day1[2]	Week 1 Day 1 (Assessment window for 2h-24h is ±15mins)												
Day/hour relative to dose 1 (Note: Day is the start of each week)	Within 28 days of Day 1	Pre-dose	Dose	1h[6]	2h	3h	4h	5h	6h	8h	10h	12h	14h	16h	24h
Informed Consent	x														
Medical History and ALS History	x														
Concomitant Medication Review	x	x													x
Pregnancy test (females only)[5]	x	x													
Urine Drug Screen[5]	x	x													
Alcohol Test[5]	x	x													
Physical examination	x	x													x
Neurological Examination	x														
ALSFRS-R	x														
Manual Muscle strength Test	x														
Electrophysiology (MUNE)	x														
Slow Inspiratory Vital Capacity (SVC)	x														
Randomization		x													
Dosing-Slow IV Infusion			x												
Adverse Events[4]	x	x	←---→												
Clinical laboratory tests	x	x[2]													x
Vital Signs (supine and standing)[3]	x	x[3]		x	x	x	x		x	x		x			x
12-Lead ECG (QT and other intervals)	x	x[10]			x		x			x		x			x
Continuous ECG Monitoring (Telemetry or Bedside ECG Monitor)[7]		←---→													
Deltoid muscle biopsy[9]	x[9]														
Blood sample for biomarkers[9]		x													
Blood sample for immunogenicity		x													
Blood sample for PK		x		x							x				x
Blood sample for PGx		x													

Continued

Table 8 Time and Events Table for Subjects in Part 1 (Continued)

Procedure[11]	Week 2	Week 4	Week 6	Week 8	Week 12	Early Withdrawal	FU[8]
Day relative to dose 1<br>(Note: Day is the start of each week)	Day<br>8	Day<br>22-28[1]	Day<br>36-42 [1]	Day<br>50-56 [1]	Day<br>78-84 [1]	Before Week 12	
Informed Consent							
Medical History and ALS History							
Concomitant Medication Review	x	x	x	x	x	x	
Pregnancy test (females only)[5]		x			x	x	
Urine Drug Screen[5]							
Alcohol Test[5]							
Physical examination					x	x	
Neurological Examination							
ALSFRS-R			x		x	x	
Manual Muscle strength Test			x		x	x	
Electrophysiology (MUNE)			x		x	x	
Slow Inspiratory Vital Capacity (SVC)			x		x	x	
Randomization							
Dosing-Slow IV Infusion							
Adverse Events[4]	x	x	x	x	x	x	
Clinical laboratory tests	x	x	x	x	x	x	
Vital Signs (supine and standing)[3]	x	x	x	x	x	x	
12-Lead ECG (QT and other intervals)	x	x	x	x	x	x	
Continuous ECG Monitoring<br>(Telemetry or Bedside ECG Monitor)							
Deltoid muscle biopsy[9]		x					
Blood sample for biomarkers[9]		x					
Blood sample for immunogenicity	x	x		x	x	x	x
Blood sample for PK	x	x	x	x	x	x	x
Blood sample for PGx							

Continued

Table 8 Time and Events Table for Subjects in Part 1 (Continued)

1. Visit window is 7 days. All assessments are to be completed within a 48 hour period
2. It is recommended that patients be admitted into the clinical unit the day before dosing day. This will allow enough time for some pre-dosing assessments. However, if necessary, patients may be admitted into the clinical unit in the morning of the dosing day. Pre-dose clinical laboratory tests will be done at **both** the Central Lab (Quest) and the Local Site Lab. Data from the Local Lab will be used to ensure the patient's clinical labs are still within the study entry range before dosing. Data from the Central Lab will be used for the database and any comparison with post dose data.
3. Pre-dose vital signs will be measured in triplicate and the mean taken as the baseline measurement. Vital signs are to be taken immediately prior to start of infusion.
4. Only SAEs related to study participation will be collected prior to start of IP. Once the IP infusion begins, all AEs and SAEs are collected through the last follow up visit.
5. Only the screening pregnancy test will be a blood test done at the central lab. All other pregnancy tests will be urine pregnancy tests done at the local lab. The drug screen and alcohol test will be performed at the local lab.
6. Study assessments to follow PK sampling at end of the infusion. (The 1hr PK sample is to be collected directly **at the end of the infusion**, cohorts 2-8)
7. Continuous Lead II ECG to commence approximately 1hr pre-dose on Day 1 until 24 hrs post dose.
8. The number and schedule of follow-up visits after Week 12 for each subject will vary depending on plasma concentrations of GSK1223249 reaching a low enough level to allow a final blood sample to be assayed for immunogenicity.
9. Muscle biopsies, which are voluntary collections in cohort 3 (1mg/kg) and required collections in cohort 5 (15mg/kg) are to be collected pre-dose and at week 4 The pre-dose muscle biopsy should only be done when the subject has passed all screening assessments and eligibility reconfirmed. This means the pre-dose biopsy can be done at any appropriate time before Day 1. Blood samples at pre-dose and at week 4 will be collected regardless of whether or not a muscle biopsy will be taken.
10. Pre-dose 12 lead ECGs will be measured in triplicate and the mean taken as the baseline measurement.
11. The precise timing of safety, PD assessments and PK blood sampling may be altered during the course of the study based on emerging data. If the profile indicates that more sampling or assessments are needed, additional timepoints will be added.

Table 9 Time and Events Table for Subjects in Part 2

Procedure[12]	Screening	Day-1/or Day 1[2]	Week 1 Day 1 (Dose 1) (Assessment window for 2h-24h is ±15mins)												
**Day/hour relative to Dose 1 (Note: Day is the start of each week)**	**Within 28 days of Day 1**	**Pre-dose**	**Dose**	**1h[6]**	**2h**	**3h**	**4h**	**5h**	**6h**	**8h**	**10h**	**12h**	**14h**	**16h**	**24h**
**Informed Consent**	x														
**Medical History and ALS History**	x														
**Concomitant Medication Review**	x	x													x
**Pregnancy test (females only)[5]**	x	x													
**Urine Drug Screen[5]**	x	x													
**Alcohol Test[5]**	x	x													
**Physical examination**	x	x													x
**Neurological Examination**	x														
**ALSFRS-R**	x														
**Manual Muscle strength Test**	x														
**Electrophysiology (MUNE)**	x														
**Slow Inspiratory Vital Capacity (SVC)**	x														
**Randomization**		x													
**Dosing-Slow IV Infusion**			x												
**Adverse Events[4]**	x	x	←---→												
**Clinical laboratory tests**	x	x[2]													x
**Vital Signs (supine and standing)[3]**	x	x[3]		x	x	x	x		x	x		x			x
**12-Lead ECG (QT and other intervals)**	x	x[10]			x		x			x		x			x
**Continuous ECG Monitoring (Telemetry or Bedside ECG Monitor)[7]**		←---→													
**Deltoid muscle biopsy[9] (Cohorts 6-7)**	x[9]														
**Deltoid muscle biopsy[10] (Cohort 8 only)**	x														x[10]
**Blood sample for biomarkers**		x													x
**Blood sample for immunogenicity**		x													
**Blood sample for PK**		x		x							x				x
**Blood sample for PGx**		x													

Continued

Table 9 Time and Events Table for Subjects in Part 2 (Continued)

Procedure[12]	Wk 2	Week 4	Week 5	Week 5 Day 29 (Dose 2) (Assessment window for 2h-6h is ±15mins)							Week 6	Wk 8	Wk 10	Wk 12	Week 16	Early With-drawal	FU[8]
Day/hour relative to Dose 1 (Note: Day is the start of each week)	Day 8	Day 22-28[1]	Day 28 or Day 29 Pre-dose[2]	Dose	1h[6]	2h	3h	4h	5h	6h	Day 36-42[1]	Day 50-56[1]	Day 64-70[1]	Day 78-84[1]	Day 106-112[1]	Before Week 16	
Informed Consent																	
Medical History and ALS History																	
Concomitant Medication Review	x	x	x								x	x	x	x	x	x	
Pregnancy test (female s only)[5]		x											x		x	x	
Urine Drug Screen[5]			x														
Alcohol Test[5]			x														
Physical examination			x							x					x	x	
Neurological Examination																	
ALSFRS-R		x										x			x	x	
Manual Muscle strength Test		x										x			x	x	
Electrophysiology (MUNE)		x										x			x	x	
Slow Inspiratory Vital Capacity (SVC)		x										x			x	x	
Randomization																	
Dosing-Slow IV Infusion				x													
Adverse Events[4]	x	x	x	←------------------------------→							x	x	x	x	x	x	
Clinical laboratory tests	x		x[2]							x	x		x		x	x	
Vital Signs (supine and standing)[3]	x	x	x[3]		X	x	x	x	x	x	x	x	x	x	x	x	
12-Lead ECG (QT and other intervals)	x	x	x[11]			x		x		x	x		x		x	x	

Continued

Table 9 Time and Events Table for Subjects in Part 2 (Continued)

Procedure[12]	Week 2	Week 4	Week 5	Week 5 Day 29 (Dose 2) (Assessment window for 2h-6h is ±15mins)							Week 6	Wk 8	Wk 10	Wk 12	Week 16	Early With-drawal	FU[8]
Day/hour relative to Dose 1 (Note: Day is the start of each week)	Day 8	Day 22-28[1]	Day 28 or Day 29 Pre-dose[2]	Dose	1h[6]	2h	3h	4h	5h	6h	Day 36-42[1]	Day 50-56[1]	Day 64-70[1]	Day 78-84[1]	Day 106-112[1]	Before Week 16	
Continuous ECG Monitoring (Telemetry or Bedside ECG Monitor)[7]			←---→														
Deltoid muscle biopsy[9] (Cohorts 6 -7)												x[9]					
Deltoid muscle biopsy[10] (Cohort 8 only)	x[10]	x[10]															
Blood sample for biomarkers	x	x										x[9]					
Blood sample for immunogenicity	x		x									x	x	x	x	x	x
Blood sample for PK	x	x	x		x					x		x	x	x	x	x	x
Blood sample for PGx																	

Table 9 Time and Events Table for Subjects in Part 2 (Continued)

1. Visit window is 7 days. All assessments are to be completed within a 48hour period
2. It is recommended that patients be admitted into the clinical unit the day before dosing day. This will allow enough time for some pre-dosing assessments. However, if necessary, patients may be admitted into the clinical unit in the morning of the dosing day. Pre-dose clinical laboratory tests will be done at **both** the Central Lab (Quest) and the Local Site Lab. Data from the Local Lab will be used to ensure the patient's clinical labs are still within the study entry range before dosing. Data from the Central Lab will be used for the database and any comparison with post dose data.
3. Pre-dose vital signs will be measured in triplicate and the mean taken as the baseline measurement.. Vital signs are to be taken immediately prior to start of infusion.
4. Only SAEs related to study participation will be collected prior to start of IP. Once the IP infusion begins, all AEs and SAEs are collected through the last follow up visit.
5. Only the screening pregnancy test will be a blood test done at the central lab. All other pregnancy tests will be urine pregnancy tests done at the local lab. The drug screen and alcohol test will be performed at the local lab.
6. Study assessments to follow PK sampling at end of infusion. (The 1hr PK sample is to be collected directly **at the end of the infusion**, cohorts 2-8)
7. Continuous Lead II ECG to commence approximately 1 hr pre-dose until 24 hrs post Dose 1 and for 6 hrs post dose 2.
8. The number and schedule of follow-up visits after Week 16 for each subject will vary depending on plasma concentrations of GSK1223249 reaching a low enough level to allow a final blood sample to be taken for immunogenicity assays.
9. In Part 2, Cohorts 6 and 7 the pre-dose muscle biopsy and blood sample should only be done when the subject has passed all screening assessments and eligibility reconfirmed. This means the pre-dose biopsy and blood sample can be done at any appropriate time before Day 1**.** The post dose muscle biopsy and blood sample is scheduled for collection at Week 8, (unless emerging data suggests the post dose muscle biopsy and blood sample should be collected at an alternative week).
10. In Part 2 Cohort 8, muscle biopsies and blood sample will be collected from subjects at pre-dose and at one timepoint after the first dose, Subjects will be assigned for a post dose muscle biopsy and blood sample collection at either Day 1(+24hr), Day 8 or Week 4 (Day 22-24) based on subject preference determined at screening (see Section 6.6.1). If the subject has the Day 1(+24hr) collection then the pre-dose muscle biopsy and blood sample must be collected at least 8 days before Day 1.
11. Pre-dose 12 lead ECGs will be measured in triplicate and the mean taken as the baseline measurement.
12. The precise timing of safety, PD assessments and PK blood sampling may be altered during the course of the study based on emerging data. If the profile indicates that more sampling or assessments are needed, additional timepoints will be added

4. STUDY POPULATION

4.1. Number of Subjects

A sufficient number of subjects will be enrolled into the study such that approximately 76 subjects complete dosing and critical assessments.

If a subject withdraws consent or is withdrawn by the investigator from the study, the subject will not be replaced (i.e., the subject number for the withdrawn subject will not be reused by another subject). However, if necessary, in order to better characterize the safety and tolerability or pharmacokinetics/pharmacodynamics of a given dose, up to 4 additional subjects may be recruited per cohort. If dose escalation to an unplanned lower dose is recommended, additional subjects/cohorts may be enrolled to allow for evaluation of additional dose levels but will not be at a higher dose than planned in this protocol.

4.2. Eligibility Criteria

4.2.1. Inclusion Criteria for Parts 1 and 2

A subject will be eligible for inclusion in this study only if all of the following criteria apply:

1. Patients with diagnosis of familial or sporadic ALS, defined as meeting the possible, laboratory-supported probable, probable, or definite criteria for a diagnosis of ALS according to the revised World Federation of Neurology El Escorial criteria [Rix Brooks, 2000].
2. Onset of muscle weakness within 60 months of study entry.
3. Patients who have low Slow Inspiratory Vital Capacity (SVC) below that what is predicted for age and sex can be included into the study at the discretion of the investigator as long as they are NOT respiratory insufficient.
4. If on any medication (including riluzole), these must have been stable within 28 days prior to dosing. (See also ‘Concomitant Medications’ – Section 8).
5. Age 18 – 80 years inclusive.
6. Male or Female of non-childbearing potential (NCBP) defined as follows:

   Pre-menopausal females with a documented tubal ligation or hysterectomy; or postmenopausal defined as 12 months of spontaneous amenorrhea [in questionable cases a blood sample with simultaneous follicle stimulating hormone (FSH) > 40 MlU/ml and oestradiol < 40 pg/ml (<140 pmol/L) is confirmatory]. Females on hormone replacement therapy (HRT) and whose menopausal status is in doubt will be required to use one of the contraception methods in Section 7.1.1, if they wish to continue their HRT during the study. Otherwise, they must discontinue HRT to allow confirmation of post-menopausal status prior to study enrollment. For most forms of HRT, at least 2-4 weeks will elapse between the cessation of therapy and the blood draw; this interval depends on the type and

dosage of HRT. Following confirmation of their post-menopausal status, they can resume use of HRT during the study without use of a contraceptive method.

7. Male subjects must agree to use one of the contraception methods listed in Section 7.1.2. This criterion must be followed from the time of the first dose of study medication until the last follow-up visit.
8. QTcB < 500 msec or uncorrected QT <600msec (machine or manual overread). If subject has bundle branch block then criteria is QTcB < 530 msec.
9. A Body Mass Index that at the discretion of the investigator is acceptable for inclusion into the study.
10. Capable of giving written informed consent, which includes compliance with the requirements and restrictions listed in the consent form.

4.2.2. Exclusion Criteria for Parts 1 and 2

A subject will not be eligible for inclusion in this study if any of the following criteria apply:

1. Patients with other neuromuscular disorders (in addition to their ALS diagnosis), unless the investigator determines that such additional disorder will not affect safety or other measures in this study.
2. Patients with evidence of dementia or psychiatric illness which, in the investigator's opinion, is likely to prevent them from a full understanding of and/or compliance with the study requirements and procedures.
3. Patients with abnormalities detected during the screening evaluations which, in the investigator's medical judgement, are sufficiently significant to exclude them from participation in the study.
4. Patients who have participated in a clinical trial involving receipt of a biopharmaceutical product within 6 months prior to the first dosing day.
5. Exposure to more than four **new investigational products** within 12 months prior to the first dosing day.
6. The subject has a positive drugs of abuse test at the screening or pre dose visit. A minimum list of drugs that will be screened for include amphetamines, barbiturates, cocaine, opiates and benzodiazepines. Subjects who are on any of these drugs by prescription for medical reasons may be considered by the investigator for inclusion if they fulfil other entry criteria.
7. The subject has a positive alcohol test at the pre-dose visit.
8. History of regular excessive alcohol consumption within 6 months of the study defined as:
    - **For European sites**: an average weekly intake of > 28 units for males or >21 units for females. One unit is equivalent to 8g of alcohol: a half-pint (~240mL) of beer, 1 glass (125mL) of wine or 1 (25mL) measure of spirits.

- **For North American sites**: an average weekly intake of >21 drinks for males or >14 drinks for women. One drink is equivalent to 12 g alcohol: 12 ounces (360mL) of beer, 5 ounces (150mL) of wine or 1.5 ounces (45mL) of 80 proof distilled spirits.

9. History of sensitivity to GSK1223249, or components thereof, or a history of drugs or other allergies that, in the opinion of the investigator or GSK Medical Monitor, contraindicates their participation.
10. Where participation in the study would result in donation of blood or blood products in excess of 500 mL within a 56 day period.
11. Females of childbearing potential, pregnant females as determined by positive serum or urine beta hCG test at screening or prior to dosing, or lactating females.
12. Patients who have received any type of vaccination in the last 3 weeks before study drug administration.
13. Unwillingness or inability to follow the procedures outlined in the protocol.
14. Subjects who will undergo muscle biopsies (cohorts 3-optional 5, 6, 7 and 8 will not be eligible for inclusion, if any of the following criteria apply:
    - Patients with wasted deltoids (MRC score ≤ 2) and patients with normal deltoids (MRC score 5).
    - Patients who cannot achieve normal coagulation in the peri-operative period and those who may otherwise be at higher risk of bleeding complications.

4.2.3. Other Eligibility Criteria Considerations

To assess any potential impact on subject eligibility with regard to safety, the investigator must refer to the following document(s) for detailed information regarding warnings, precautions, contraindications, adverse events, and other significant data pertaining to the investigational product(s) being used in this study: Investigators Brochure (IB).

5. DATA ANALYSIS AND STATISTICAL CONSIDERATIONS

Statistical analyses will be performed by, or under the direct auspices of, Discovery Statistics, GlaxoSmithKline.

5.1. Hypotheses and Treatment Comparisons

This study is designed to investigate the safety, tolerability and pharmacokinetics of single and repeat doses of GSK1223249. No formal statistical hypotheses are to be tested. Where appropriate, an estimation approach will be taken, and point estimates and 95% confidence intervals will be constructed.

5.2. Sample Size Considerations

5.2.1. Sample Size Assumptions

There was no formal calculation of power or sample size. The sample size is based on safety and feasibility. Cohorts in Part 1 are small to limit the number of subjects randomized to a treatment they are highly unlikely to derive benefit from with a single dose for this serious and life-threatening disease.

5.2.2. Sample Size Sensitivity

No sample size sensitivity was performed.

5.2.3. Sample Size Re-estimation

No sample size re-estimation will be performed.

5.3. Data Analysis Considerations

5.3.1. Interim Analysis

As stated in Section 3.2, the decision to escalate to a higher dose will be made by the DEC based on review of preliminary blinded pharmacokinetic and safety data from the current dose (and the preceding doses, where applicable).

A blinded interim analysis is planned to consolidate the safety and pharmacokinetic data for cohorts 1 to 4 from Part 1 of the study.

5.3.2. Final Analyses

Safety Analyses

Safety data will be presented in tabular and/or graphical format and summarized descriptively according to GSK's Integrated Data Standards Library (IDSL) standards.

Pharmacokinetic Analyses

Pharmacokinetic analysis will be the responsibility of the Clinical Pharmacokinetics Modeling and Simulation Department, CPMS, GlaxoSmithKline. GSK1223249 Plasma concentration-time data will be analyzed by non-compartmental methods with WinNonlin [version 5.2]. Calculations will be based on the actual sampling times recorded during the study.

Linear and semi-logarithmic individual plasma concentration time-profiles and mean and median profiles by GSK1224349 dose will be plotted for quantifiable concentrations. Plasma concentration will be listed and summarized by dose and nominal times.

From the plasma concentration-time data, the following pharmacokinetic parameters will be determined, as data permit: maximum observed plasma concentration (C_{max}), area under the plasma concentration-time curve $AUC_{(0\text{-Week }4)}$ and $AUC_{(0\text{-}\infty)}$, and apparent

terminal phase half-life (t1/2). $AUC_{(0-\infty)}$, $AUC_{(0\text{-Week-4})}$, Cmax, or $C_{week\ 4}$ following single and repeat doses may be used for assessment of dose proportionality.
A mixed effects model will be fitted to assess dose proportionality of AUC(0-t) Cmax, $AUC_{0\text{-week 4}}$, $C_{week\ 4}$, and CL using a Power Model. Dose proportionality requires that slope ß is close to unity for dose independent parameters. The estimate of slope with respect to log(dose) together with 90% confidence interval (ßL, ßU) will be used to quantify the degree of nonproportionality.

PK parameters will be summarised and listed by each dose regimen. Summary statistics will include: n, arithmetic mean, standard deviation, minimum, median, maximum, and 95% confidence intervals about the arithmetic mean. The geometric mean, a 95% confidence interval about the geometric mean and the between-subject coefficient of variation (CVb) will also be provided for each dose and day for all log transformed parameters. The CVb will be estimated as follows:

CVb (%) = sqrt[exp(sd^2) – 1] x 100

where sd is the standard deviation of the log-transformed data.

All pharmacokinetic data will be stored in the Archives, GlaxoSmithKline Pharmaceuticals, R&D.

Statistical analyses of the pharmacokinetic parameter data will be the responsibility of Discovery Biometrics, GlaxoSmithKline.

Pharmacokinetic/Pharmacodynamic Analyses

For Part 2 of the study, exploratory PK/PD analyses will be performed to examine the potential relationships between GSK1223249 pharmacokinetics and markers of safety or biological activity (*e.g.* change from baseline in ALSFRS-R, and SVC).

Immunogenicity Analyses

The final results of the anti-GSK1223249 antibody tests will be listed and summarised descriptively by dose.

Novel Biomarker(s) Analyses

The results of these biomarker investigations will be reported separately from the main clinical study report. All endpoints of interest from all comparisons will be descriptively and/or graphically summarized as appropriate to the data.

Additional exploratory analyses may be performed to further characterize the novel biomarker.

RNA Expression Analysis

RNA expression profile data will first be normalized to enable direct comparison of all data sets. Uninformative data (RNA species in all samples below detectable limits or levels unchanged across all samples under comparison) will be removed and multivariate statistical analyses may be performed on the remaining data to uncover intrinsic differences and similarities in the levels of RNAs between the different samples, and groups of samples. Statistical tools such as Principal Component Analysis, PLS-Discriminant Analysis, and ANOVA using either standard or customized software will be used for the profile analysis to assist identification of patterns/profiles which may associate with treatment outcome and/or disease.

Pharmacogenetic Analyses

The presence/absence of genetic variations in selected candidate genes in deoxyribonucleic acid (DNA) from blood may be analyzed to determine their relationship with response (safety, tolerability, pharmacokinetics, and efficacy) to treatment.

The results of PGx investigations will be reported as a separate report. All endpoints of interest from all comparisons will be descriptively and/or graphically summarized as appropriate to the data. In all cases, appropriate statistical methods will be used to analyze the genetic markers in the context of other clinical data.

Functional and Electrophysiological Endpoints Analyses

Data from the following endpoints will be presented in graphical and/or tabular form and will be listed and summarized descriptively by dose:

ALSFRS-R, SVC, MMT, MUNE

Additional exploratory analyses may be performed to further characterize these endpoints for Part 2.

6. STUDY ASSESSMENTS AND PROCEDURES

After providing full informed consent, subjects will undergo a medical screen to determine their eligibility for participation based on the criteria outlined in this protocol. Subjects will be screened within 28 days prior to administration of study medication. The study investigator or a sub-investigator will discuss with each subject the nature of the study, its requirements, and its restrictions. Written and witnessed informed consent must be obtained prior to any protocol specific procedures. It is recommended that patients be admitted into the clinical unit the day before dosing day. This will allow enough time for some pre-dosing assessments. However, if necessary, patients may be admitted into the clinical unit in the morning of the dosing day.

The following sections list the parameters of each planned study assessment. The exact timing of each assessment is listed in the Time and Events Table (Table 8 and Table 9). Detailed procedures for obtaining each assessment are provided in the Study Procedures Manual (SPM). Whenever vital signs, 12-lead ECGs and blood draws are scheduled for the same nominal time, the PK sample will be obtained first followed by vital signs, 12 Lead ECG and then clinical laboratory blood draws.

The timing and number of planned study assessments may be altered during the course of the study based on newly available data (e.g. to obtain data closer to the time of peak plasma concentrations) to ensure appropriate monitoring. The change in timing or addition of time points for any planned study assessments must be approved and documented by GSK, but this will not constitute a protocol amendment. The IRB/IEC will be informed of any safety issues that require alteration of the safety monitoring scheme. The total volume of blood that will be collected for each subject in Part 1 is approximately 250mL and for Part 2 approximately 350mL. Further details are listed in the Study Procedures Manual (SPM).

6.1. Demographic/Medical History Assessments

The following demographic parameters will be captured and recorded in the eCRF: date of birth, gender, race and ethnicity. Additionally ALS History will be captured and recorded in the eCRF.

Medical/medication/alcohol history will be assessed as related to the eligibility criteria listed in Section 4.2

6.2. Safety

Planned timepoints for all safety assessments are listed in the Time and Events Table (Table 8 and Table 9). Additional time points for safety tests, PK, PD, immunogenicity sampling and other disease assessments or questionnaires may be added during the course of the study based on newly available data to ensure appropriate safety monitoring. Any abnormalities of clinical significance should be followed by the investigator until resolution, the condition stabilises, the event is otherwise explained or the subject is lost to follow up.

6.2.1. Physical Examination

A complete physical examination will be performed by medically qualified personnel at those time points as described in the Time and Events Table, (Table 8 and Table 9). The examination is to include assessments of the head, eyes, ears, nose, throat, skin, thyroid, neurological, lungs, cardiovascular, abdomen (liver and spleen), lymph nodes and extremities. Height (in centimeters) and weight (in kilograms) will also be measured. All findings are to be documented in the source documents and any new abnormalities or clinically significant worsening found via the physical examination after the start of investigational product are to be recorded on the AE/SAE page of the eCRF.

6.2.2. Neurological Examination

A complete neurological examination will be performed according to local site protocol by medically qualified personnel at those time points as described in the Time and Events Tables, (Table 8 and Table 9). All findings are to be documented in the source documents.

6.2.3. Vital Signs

Heart rate and blood pressure (systolic and diastolic) measurements will be collected in the supine and standing position by qualified personnel at the time points described in the Time and Events Tables, (Table 8 and Table 9) and recorded in the eCRF. The number of assessments may be adjusted depending upon the emerging safety profile in man and additional assessments may be added. Heart rate and blood pressure measurements will be made with automated monitors with the subject in two positions:

- supine - subjects must be lying flat with maximum of one pillow for 10 minutes prior to each reading
- standing - subjects must be standing for 1 minute prior to each reading.

Pre-dose heart rate and blood pressure should be measured in triplicate and the mean taken as the baseline measurement. Measurements that deviate substantially in the opinion of the supervising physician from previous readings will be repeated. The responsible physician, or deputy, will review the data at intervals throughout the day.

6.2.4. Electrocardiogram (ECG)

Single 12-lead ECGs will be obtained at each time point during the study as described in the Time and Events Table, (Table 8 and Table 9). An ECG machine that automatically calculates the heart rate and measures PR, QRS, QT, RR and QTc intervals is required for this study. If the ECG machine does not automatically calculate RR or QTc(B), then manual measurement of the parameter is acceptable. The number of assessments may be adjusted depending upon the emerging safety in man, and additional assessments may be added. **All 12-Lead ECGs must be stored electronically for manual measurement of intervals if necessary.**

Supine 12 lead ECGs will be made after the subject has been resting supine (lying flat with maximum one pillow) for a period of at least 10 minutes. Pre-dose 12 lead ECGs should be measured in triplicate and the mean taken as the baseline measurement.

Continuous Lead II ECG monitoring (using telemetry or a bedside ECG monitor) will also be performed during the study as described in the Time and Events Table, (Table 8 and Table 9). Any abnormal findings on continuous ECG monitoring should be confirmed by 12-lead ECG.

The investigator or other medically qualified study team member will evaluate the screening ECG for any abnormalities that exclude the subject from the study. All subsequent ECGs are to be evaluated for any new abnormalities or clinically significant worsening. The ECG assessment date, presence or absence of abnormalities, clinical significance of abnormalities and type of any clinically significant abnormalities will be recorded in the eCRF. Additionally, PR, QRS, QT and RR intervals will be recorded in the eCRF. A printout of each ECG should be made and retained in the source documents for each subject.

The same make and model machine should be used for the same subject throughout the study.

Refer to Section 3.3.2 for QTc withdrawal criteria and additional QTc readings that may be necessary.

6.2.5. Clinical Laboratory Assessments

The following routine laboratory tests will be performed at those time points as specified in Time and Events Table, (Table 8 and Table 9). All samples will be sent to a central laboratory for analysis except for the urine pregnancy test and urine drug screen (which will be performed at the local study site). Pre-dose clinical laboratory tests will be done at **both** the Central Lab (Quest) and the local lab.

**Haematology**		
Platelet Count	*RBC Indices:*	*Automated WBC Differential:*
RBC Count	MCV	Neutrophils
WBC Count (absolute)	MCH	Lymphocytes
Reticulocyte Count (absolute)	MCHC	Monocytes
Haemoglobin		Eosinophils
		Basophils
**Coagulation**		
PT		
APPT		

Clinical Chemistry			
BUN	Potassium	AST (SGOT)	Total and direct bilirubin
Creatinine	Chloride	ALT (SGPT)	Uric Acid
Glucose, random	Total CO_2	GGT	Albumin
Sodium	Calcium	Alkaline phosphatase	Total Protein
CPK		C-Reactive Protein	
**Endocrinology**			
TSH			

Routine Urinalysis
Specific gravity
pH, glucose, protein, blood and ketones
Microscopic examination (if blood or protein is abnormal)

Other tests (Screening Visit Only)
HIV
Hepatitis B (HBsAg)
Hepatitis C (Hep C antibody -- if second generation Hepatitis C antibody positive, a hepatitis C antibody Chiron RIBA immunoblot assay should be reflexively performed **on the same sample** to confirm the result)
FSH and oestradiol (as needed in women of non-child bearing potential only)

At the Principal Investigator and GSK Medical Monitor's discretion, additional laboratory tests other those outlined above may be included to further assess safety and tolerability. Additionally, repeat or retest samples may be collected at the discretion of the investigator or at the request of the GSK Medical Monitor. All repeat or retest samples will be sent to the central laboratory for testing. See the Central Laboratory Manual and SPM for full details of sample collection, handling and shipment.

Results will be reported back to the investigator and a copy of the results is to be maintained in the subject's source documents. The investigator will be responsible for establishing if any abnormalities are of clinical significance. Any abnormalities of clinical significance should be followed by the investigator until resolution, until the condition stablises, until the event is otherwise explained or until the subject is lost to follow up. The investigator should also refer to the IP stopping criteria and monitoring criteria as outlined in Section 3.3.2 and Section 12.

6.2.6. Urine collection for the Detection of Undeclared Drugs

A urine sample for an undeclared drugs screen will be collected and tested at the local lab at screening and on entry to the unit pre-dose (see SPM for details).

6.2.7. Alcohol Test

An alcohol urine or breath test will be collected and tested at the local lab at screening and on entry to the unit pre-dose (see SPM for details).

6.2.8. Pregnancy Test

A blood sample for a pregnancy test will be collected at screening and will be tested at the central lab. All other pregnancy tests will be urine pregnancy tests and tested at the local lab. (see SPM for details).

6.3. Pregnancy

6.3.1. Time period for collecting pregnancy information

Information on all pregnancies in female subjects and/or female partners of male subjects will be collected after the start of dosing and until 5 terminal half-lives post last dose.

6.3.2. Action to be taken if pregnancy occurs

The investigator will collect pregnancy information on any female subject, who becomes pregnant while participating in this study. The investigator will record pregnancy information on the appropriate form and submit it to GSK within 2 weeks of learning of a subject's pregnancy. The subject will also be followed to determine the outcome of the pregnancy. Information on the status of the mother and child will be forwarded to GSK. Generally, follow-up will be no longer than 6 to 8 weeks following the estimated delivery date. Any premature termination of the pregnancy will be reported.

While pregnancy itself is not considered to be an AE or SAE, any pregnancy complication or elective termination of a pregnancy for medical reasons will be recorded as an AE or SAE (see AE/SAE, Section 11, of the protocol and the SPM for definitions and a description of follow-up).

A spontaneous abortion is always considered to be a SAE and will be reported as such. Furthermore, any SAE occurring as a result of a post-study pregnancy and is considered reasonably related to the investigational product by the investigator, will be reported to GSK as described in section entitled, "Post-study AEs and SAEs" of the SPM. While the investigator is not obligated to actively seek this information in former study participants, he or she may learn of an SAE through spontaneous reporting.

Any female subject who becomes pregnant while participating will discontinue study medication.

6.3.3. Action to be taken if pregnancy occurs in a female partner of a male study subject

The investigator will attempt to collect pregnancy information on any female partner of a male study subject who becomes pregnant while participating in this study. The investigator will record pregnancy information on the appropriate form and submit it to GSK within 2 weeks of learning of the partner’s pregnancy. The partner will also be followed to determine the outcome of the pregnancy. Information on the status of the mother and child will be forwarded to GSK. Generally, follow-up will be no longer than 6 to 8 weeks following the estimated delivery date. Any premature termination of the pregnancy will be reported.

6.4. Pharmacokinetics

6.4.1. Blood Sample Collection

Blood samples for pharmacokinetic analysis of GSK1223249 will be collected at the time points indicated in (Table 8 and Table 9), Time and Events Tables. The actual date and time of each blood sample collection will be recorded in the CRF.

GSK may alter the timing of PK samples and/or request that PK samples be obtained at additional time points to ensure thorough PK monitoring.

Details of PK blood sample collection (including volume to be collected), processing, storage and shipping procedures are provided in the Study Procedures Manual (SPM).

6.4.2. Sample Analysis

Plasma analysis will be performed under the management of Worldwide Bioanalysis, DMPK, GlaxoSmithKline. Concentrations of GSK1223249 will be determined in plasma samples using the currently approved analytical methodology. Raw data will be stored in the GLP Archives, GlaxoSmithKline.

6.5. Functional and Electrophysiological Assessments

6.5.1. Functional Assessments

Medical Research Council Muscle Strength Scale

Subjects will be required (with the assistance of the investigator or designee) to complete the Manual Muscle Testing Scale as described in the Time and Events Table, (Table 8 and Table 9). The subjects muscle strength will be assessed via Manual Muscle Testing (MMT) by trained personnel. The subject's handedness will be recorded. The biopsy sample will be taken from the deltoid muscle whose strength should be grade 3 or 4 on the MRC scale. Patients with wasted deltoids (MRC score ≤2) and normal deltoids MRC score 5 shall be excluded from the study. For details of the MRC Manual Muscle Testing and how to score, see the SPM.

ALS Functional Rating Scale-Revised

Subjects will be required (with the assistance of the investigator or designee) to complete the ALSFRS-R as described in the Time and Events Table, (Table 8 and Table 9) [Fuller, 1999]. For details of the ALSFRS-R and how to administer and score the ALSFRS-R see the SPM.

Slow Inspiratory Vital Capacity

Slow Inspiratory Vital Capacity (SVC) will be measured by trained personnel using a validated spirometer as described in the Time and Events Table, (Table 8 and Table 9). For details of the procedure and how to measure SVC, see the SPM.

6.5.2. Electrophysiological Assessment (MUNE)

Motor Unit Number Estimation (MUNE) will be collected during the study as described in the Time and Events Table, (Table 8 and Table 9). MUNE is a non invasive method that assesses the number of functional motor units in a muscle of interest, as well as estimating the average amplitude of motor units in that muscle. MUNE can be performed using force measurements, but most commonly depends on recording both compound and single unit muscle action potentials from surface recording electrodes placed over the muscle. For either force or electrical recording, the amplitude of a single motor unit is a direct reflection of the number of muscle fibers innervated by the motor axon. Thus, motor unit amplitude is directly relevant to measuring the effect of an agent expected to having an effect on terminal axon sprouting. Motor unit estimation is most applicable to small muscles of the hand or foot, and can be performed using standard EMG machines. Training is required of all personnel performing MUNE; with appropriate training and validation, test-retest variability can be reduced to less than 20%. It takes approximately 20 minutes to perform MUNE on a single muscle. For details of the MUNE and how to administer the technique, see the SPM.

6.6. Biomarker(s)/Pharmacodynamic Markers

6.6.1. Skeletal Muscle Biopsies and Blood Samples

Biomarker blood samples and skeletal muscle biopsy samples will be taken from patients for evaluation of a variety of pharmacodynamic (PD) biomarkers as described in the objectives and endpoint table (Table 5). The muscle biopsies will be taken from the weaker deltoid muscle. The strength of the muscle from which the biopsy is taken should be Grade 3 or 4 in the MRC scale. Patients with wasted deltoids (MRC ≤ 2) will be excluded from the study. Both biopsies should be taken from the same deltoid muscle if at all possible. If not possible, for medical reasons, then the second biopsy can be taken from the opposite deltoid muscle provided the muscle strength is Grade 3 or 4 (MRC scale). Specific details of the procedures for taking and handling the biopsy samples are provided in the Study Procedures Manual (SPM).

Subjects having muscle biopsies (as identified in Table 6) will have two muscle biopsies taken: one at pre-dose and one post the first dose; in addition a biomarker blood sample will be drawn.

- For cohorts 3 and 5: the post-dose timepoint is Week 4 Days 22-28 (see Table 8)
- For cohorts 6 and 7: the post-dose timepoint is Week 4 Days 22-28 (see Table 9)
- For cohort 8 only, the schedule for the post dose muscle biopsies will comprise of 3 subgroups. Each subgroup (or stratum) will be assigned to a specific timepoint and will include 4 subjects; within each stratum, subjects will be randomized to placebo or active in a 1:3 ratio (stratified randomization). Subjects will be assigned to a subgroup based on subject preference determined at screening (see Table 10):

Table 10 Cohort 8 Muscle Biopsies Randomization Schedule

Cohort 8 (n=12)	n=4	Stratum 1	Day 1 (+ 24hr) after 1st dose
	n=4	Stratum 2	Day 8 after 1st dose
	n=4	Stratum 3	Day 22-28 after 1st dose

6.6.2. Exploratory Biomarkers

With the subject's consent, blood and tissue sample(s) will be collected during this study and may be used for the purposes of measuring novel biomarkers to identify factors that may influence ALS, and/or medically related conditions, as well as the biological and clinical responses to GSK1223249. If relevant, this approach will be extended to include the identification of biomarkers associated with adverse events.

Samples will be collected at the timepoints indicated in (Table 8 and Table 9). The timing of the collections may be adjusted on the basis of emerging PK or PD data from this study or other new information in order to ensure optimal evaluation of the PD endpoints. See SPM for details of the processing of these samples.

Novel candidate biomarkers and subsequently discovered biomarkers of the biological response associated with ALS patient blood or tissue and/or the action of GSK1223249 will be identified by application of:

- RNA analysis of muscle samples.
- Protein analysis of plasma and muscle samples.
- Ex vivo immunogenicity analysis of serum samples.
- Immunohistochemical analysis of tissue sections from muscle samples.

All samples will be retained for a maximum of 15 years after the last subject completes the trial.

RNA Expression Research

RNA expression studies may be conducted using quantitative RT-PCR, and/or microarrays and/or alternative equivalent technologies, which can facilitate the simultaneous measurement of the relative abundances of RNA species resulting in a RNA expression profile for muscle samples. This will enable the evaluation of changes in RNA profiles that may correlate with biological response relating to ALS or the action of GSK1223249.

Protein Research

Plasma and muscle protein studies may be performed by Western blotting and/or ELISA, or an alternative equivalent procedure. This will enable the evaluation of changes in

protein profiles that may correlate with biological response relating to ALS. The same samples may also be used to confirm findings by application of alternative technologies.

Immunohistochemistry

Frozen sections of the muscle biopsies will be examined by immunohistochemistry and/or Laser Scanning Cytometry for expression of protein including NOGO-A. This will enable the evaluation of changes in protein profiles that may correlate with biological response to ALS. Following an acceptable pathology QC check of the muscle biopsies from the investigator site, the same tissue sample shall be shipped from Department of Clinical Neuropathology, Kings College Hospital, London to GSK.

6.7. Immunogenicity (Anti-Drug Antibodies)

An important component of the early clinical development of biopharmaceutical compounds is assessment of the development of anti-drug antibodies (ADA), if any. An immunogenicity assay has been developed to detect any ADAs to GSK1223249. Blood samples will be taken from patients in both Parts 1 and 2 for immunogenicity assays at the time points shown in the Table and Events Table (Table 8 and Table 9). The performance of the immunogenicity is best below a threshold concentration of GSK1223249. This threshold concentration will be established in the final validation of the assay. Therefore, the number and schedule of the final follow-up visit for each subject will vary depending on plasma concentrations of GSK1223249 reaching a low enough level to allow a final blood sample to be taken for immunogenicity assays. Subject will then be fully discharged from the study.

6.8. Pharmacogenetics

Information regarding pharmacogenetic (PGx) research is included in Appendix 1. The IRB/IEC and, where required, the applicable regulatory agency must approve the PGx assessments before these can be conducted at the site. In some cases, approval of the PGx assessments can occur after approval is obtained for the rest of the study. If so, then the written approval will clearly indicate approval of the PGx assessments is being deferred and in most cases, the study, except for PGx assessments, can be initiated. When PGx assessments will not be approved, then the approval for the rest of the study will clearly indicate this and therefore, PGx assessments will not be conducted.

7. LIFESTYLE AND/OR DIETARY RESTRICTIONS

7.1. Contraception Requirements

7.1.1. Female Subjects

Females of non child bearing potential on hormone replacement therapy (HRT) and/or whose menopausal status is in doubt will be required to use one of the contraception methods below:-

Abstinence

Sexual inactivity by abstinence must be consistent with the preferred and usual lifestyle of the subject. Periodic abstinence (e.g. calendar, ovulation, symptothermal, post-ovulation methods) and withdrawal are not acceptable methods of contraception

Contraceptive Methods with a Failure Rate of <1%

- Oral contraceptive, either combined or progestogen alone
- Injectable progestogen
- Implants of levonorgestrel
- Estrogenic vaginal ring
- Percutaneous contraceptive patches
- Intrauterine device (IUD) or intrauterine system (IUS) that meets the <1% effectiveness criteria as stated in the product label
- Male partner sterilization (vasectomy with documentation of azoospermia) prior to the female subject's entry into the study, and this male is the sole partner for that subject. For this definition, “documented” refers to the outcome of the investigator's/designee’s medical examination of the subject or review of the subject's medical history for study eligibility, as obtained via a verbal interview with the subject or from the subject’s medical records.
- Double barrier method: condom and occlusive cap (diaphragm or cervical/vault caps) plus spermicidal agent (foam/gel/film/cream/suppository)

These allowed methods of contraception are only effective when used consistently, correctly and in accordance with the product label. The investigator is responsible for ensuring subjects understand how to properly use these methods of contraception.

7.1.2. Male Subjects

To prevent pregnancy in a female partner or exposure of any partner to the investigational product from a male subjects’ semen, male subjects must use one of the following contraceptives methods:

- Abstinence, defined as sexual inactivity consistent with the preferred and usual lifestyle of the subject. Periodic abstinence (e.g. calendar, ovulation, symptothermal, post-ovulation methods) and withdrawal are not acceptable methods of contraception
- Condom (during non-vaginal intercourse with any partner- male or female) OR
- Condom and occlusive cap (diaphragm or cervical/vault caps) plus spermicidal agent (foam/gel/film/cream/suppository) (during sexual intercourse with a female)

7.2. Alcohol and Tobacco

- During each dosing session, subjects will abstain from alcohol for 24 hours prior to the start of dosing and prior to collection of the pharmacokinetic and or pharmacodynamic samples during each visit.

- Subjects who use tobacco products will be instructed that use of nicotine-containing products (including nicotine patches) will not be permitted while they are in the Clinical Unit.

7.3. Activity

Subjects will abstain from strenuous exercise for 48 hours prior to each blood collection for clinical laboratory tests. Subjects may participate in light recreational activities during studies (e.g., watch television, read).

8. CONCOMITANT MEDICATIONS AND NON-DRUG THERAPIES

8.1. Permitted Medications

Subjects may continue to receive all medications which were started before study participation provided they are not one of the prohibited medications listed in Section 8.2. Subjects taking riluzole, statins or other prescribed medication prior to screening are permitted to continue receiving that medication; however, no dose changes should be made within 28 days prior to dosing or during the subject's study participation. **NOTE:** that if a subject is on medication likely to inhibit coagulation and this cannot be safely suspended for the purpose of the biopsies, then they do not fulfil eligibility criterion number 9. Where there is doubt about any concomitant medication, the Investigator should consult the GSK Medical Monitor to discuss the issue before entering the subject. All medications taken since the time of randomization through the last study visit will be recorded in the eCRF – this includes vitamins, herbal supplements, etc.

8.2. Prohibited Medications

The following medications are prohibited:

- Systemic immunosupressants
- New investigational products as noted in the entry criteria

9. COMPLETION OR EARLY WITHDRAWAL OF SUBJECTS

9.1. Subject Completion

A completed subject is one who has died before Week 12 (Part 1) or Week 16 (Part 2), or completed visits through Week 12 (Part 1) or Week 16 (Part 2), and if needed any additional unscheduled follow-up visits that may be required for further immunogenicity monitoring. A subject can withdraw from investigational product but still complete the study by attending all required visits.

9.2. Subject Withdrawal Criteria

Refer to Section 3.3 for dose adjustment/stopping criteria based on safety/PK/PD criteria.

A subject may voluntarily discontinue investigational product or participation in this study at any time. The investigator may also, at his or her discretion, discontinue investigational product or the subject from participating in this study at any time. A subject may withdraw (or be withdrawn) from the study prematurely for the following reasons:

- Subject voluntarily discontinues participation in the study (consent withdrawal)
- Unacceptable adverse events.
- Protocol deviation (including non-compliance)
- Lost to follow-up
- Termination of the study by the sponsor
- Other (must be specified)

A subject may withdraw (or be withdrawn) from investigational product for those reasons as listed above. Additionally, investigational product may be stopped for the safety, PK and PD criteria as listed in Section 3.3

9.3. Subject Withdrawal Procedures

9.3.1. Subject Withdrawal from Study

If a subject is prematurely discontinued from participation in the study for any reason, the investigator must make every effort to perform the evaluations planned for the Early Withdrawal visit. Please see the Time and Events Table in Table 8 and Table 9 for the list of planned assessments at this visit. These data will be recorded, as they comprise an essential evaluation that needs to be done prior to discharging any subject from the study.

In the event that a subject is prematurely discontinued from the study at any time due to an AE (as defined in Section 11.1, "Definition of an AE") or SAE (as defined in Section 11.2, "Definition of a SAE"), the procedures stated in Section 11 ("AEs and SAEs") must be followed.

9.3.2. Subject Withdrawal from Investigational Product

In the event investigational product is prematurely discontinued, all protocol assessments up to the point of investigational product discontinuation must be completed and the subject should be continued in the study through the Week 12 visit, if in Part 1 or the Week 16 visit if in Part 2 and if at all possible, subjects should be encouraged to attend and provide a final blood sample for PK and immunogenicity assays.

9.4. Treatment After the End of the Study

A compassionate use protocol may be developed once this study has started. This will enable patients who participated in this study to receive further treatment, on a compassionate basis, if they so desire and if the investigator agrees that such treatment may be useful for the patient. The timing of availability of the compassionate use protocol will depend on approval from GSK and other necessary authorities.

9.5. Screening Failures

Data for screen failures, such as reason for screen failure will be collected.

10. INVESTIGATIONAL PRODUCT(S)

Investigational product dose and administration details are listed in Table 7.

10.1. Blinding

This will be a double-blind study. All patients, study site staff (excluding staff randomizing subjects and preparing the infusions) and GSK study team staff will remain blinded. The study site staff assigned to randomise subjects must maintain the blind and not share the treatment assignment with other members of the site study team or GSK. By handling and preparing investigational product, the pharmacist will become unblinded to treatment assignment; however, they must maintain the blind and provide investigational product in a blinded fashion to the study personnel administering the infusions (e.g., masked infusion sets, same sized infusion bags and volumes used for GSK1223249 and saline placebo solutions, etc)

In the case of a **medical emergency or in the event of a serious medical condition**, when knowledge of the investigational product is essential for the clinical management or welfare of the subject, **an investigator or other physician managing the subject may decide to unblind that subject's treatment code**. The investigator will make every effort to contact the GSK Medical Monitor or appropriate GSK study personnel before unblinding to discuss options. If the blind is broken for any reason and the investigator is unable to contact GSK prior to unblinding, the investigator must notify GSK **as soon as possible following** the unblinding incident **without revealing the subject's study treatment assignment,** unless the information is important to the safety of subjects remaining in the study. In addition, the investigator will record the date and reason for revealing the blinded treatment assignment for that subject in the appropriate data collection tool (as defined in Section 13.7).

If a serious adverse event (SAE; as defined in Section 11.2, "Definition of an SAE") is reported to GSK, the independent members of the DEC may unblind the treatment assignment for the individual subject. If an expedited regulatory report to one or more regulatory agencies is required, the report will identify the subject's treatment assignment. When applicable, a copy of the regulatory report may be sent to investigators in accordance with relevant regulations, GSK policy, or both.

10.2. Blinding and Dose Escalation Committee (DEC)

A dose escalation committee (DEC), consisting of GSK staff plus an external ALS neurologist, will review key safety data before recommending escalation to the next dosing cohort. Should the DEC require unblinding, the core GSK study team staff will remain blinded and remove themselves from the review. Table 11 shows the composition of the DEC with those who will remain blinded.

Table 11 Dose Escalation Committee

Dose Escalation Committee		
**Role**	**Removed from Blind**	**Remain Blinded**
Medical Monitor		X
Therapeutic Area Representative		X
Clinical Science and Study Operations		X
Global Clinical Safety and Pharmacovigilance (GCSP)		X
Study Statistician		X
Clinical Pharmacology Modeling & Simulation (CPMS)[a]	X	
Independent[b] Statistician	X	
Independent[b] GSK Neurologist/Neuroscience Physician	X	
Independent[b] External ALS Neurologist	X	

a. The CPMS expert is responsible for presenting PK data in an anonymised format such that individual patients cannot be identified. Although the CPMS expert is a member of the GSK study team, he/she will not have direct contact with site personnel or activities.
b. 'Independent' is defined as an expert who is external to the GSK study team, without direct contact to site personnel or activities.

10.3. Packaging and Labeling

The contents of the label will be in accordance with all applicable regulatory requirements.

10.4. Preparation/Handling/Storage/Accountability

A description of the methods and materials required for GSK1223249 are provided in the SPM.

Investigational product must be dispensed or administered according to procedures described herein. Only subjects enrolled in the study may receive investigational product. Only authorized site staff may supply or administer investigational product. All investigational products must be stored in a secure area with access limited to the investigator and authorized site staff. Investigational product is to be stored at 2-8°C. Protect from light and do not freeze. Maintenance of a temperature log (manual or automated) is required.

The investigator, institution, or the head of the medical institution (where applicable) is responsible for investigational product accountability, reconciliation, and record maintenance. The investigator or the head of the medical institution (where applicable), or designated site staff (e.g., storage manager, where applicable) must maintain investigational product accountability records throughout the course of the study. The responsible person(s) will document the amount of investigational product received from and returned to GSK and the amount administered to subjects. The required accountability unit for this study will be one clear, glass vial. Discrepancies are to be

reconciled or resolved. Procedures for final disposition of unused investigational product are listed in the SPM.

Investigational product is not expected to pose significant occupational safety risk to site staff under normal conditions of use and administration. A Material Safety Data Sheet (MSDS)/equivalent document describing occupational hazards and recommended handling precautions either will be provided to the investigator, where this is required by local laws, or is available upon request from GSK.

However, precautions are to be taken to avoid direct skin contact, eye contact, and generating aerosols or mists. In the case of unintentional occupational exposure notify the monitor, medical monitor and/or study manager.

10.5. Assessment of Compliance

When subjects are dosed at the study site, they will receive investigational products directly from the investigator or designee, under medical supervision. The date and time of each dose administered in the clinic will be recorded in the source documents. The dose of investigational product(s) will be confirmed prior to dosing by a member of the study site staff other than the person administering the investigational product.

10.6. Treatment of Investigational Product Overdose

An overdose is defined as any dose greater than that specified in the randomization code for that subject. GSK does not recommend specific treatment for an overdose. The investigator will use clinical judgment to treat any overdose. Any overdose must be reported to the GSK Medical Monitor and documented in the Study File.

11. ADVERSE EVENTS (AE) AND SERIOUS ADVERSE EVENTS (SAE)

The investigator or site staff is responsible for detecting, documenting and reporting events that meet the definition of an AE or SAE.

AEs will be collected from the time of dosing until the last follow-up visit. Medical occurrences that begin prior to the start of investigational product but after obtaining informed consent may be recorded on the Medical History/Current Medical Conditions source documents.

SAEs will be collected over the same time period as stated above for AEs. However, any SAEs assessed as related to study participation (e.g. investigational product, protocol-mandated procedures, invasive tests, or change in existing therapy) or related to a GSK concomitant medication will be recorded from the time a subject consents to participate in the study up to and including any follow-up contact. All SAEs will be recorded and reported to GSK within 24 hours, as indicated in Section 11.8.

Investigators are not obligated to actively seek AEs or SAEs in former study participants. However, if the investigator learns of any SAE, including a death, at any time after a subject has been discharged from the study, and he/she considers the event reasonably

related to the investigational product or study participation, the investigator would promptly notify GSK.

11.1. Definition of Adverse Events

An AE is any untoward medical occurrence in a patient or clinical investigation subject, temporally associated with the use of a medicinal product, whether or not considered related to the medicinal product.

Note: An AE can therefore be any unfavorable and unintended sign (including an abnormal laboratory finding), symptom, or disease (new or exacerbated) temporally associated with the use of a medicinal product.

Events meeting the definition of an AE **include**:

- Any abnormal laboratory test results (haematology, clinical chemistry, or urinalysis) or other safety assessments (e.g., ECGs, radiological scans, vital signs measurements), including those that worsen from baseline, and felt to be clinically significant in the medical and scientific judgement of the investigator.
- Exacerbation of a chronic or intermittent pre-existing condition including either an increase in frequency and/or intensity of the condition.
- New conditions detected or diagnosed after investigational product administration even though it may have been present prior to the start of the study.
- Signs, symptoms, or the clinical sequelae of a suspected interaction.
- Signs, symptoms, or the clinical sequelae of a suspected overdose of either investigational product or a concomitant medication (overdose per se will not be reported as an AE/SAE).

Events that **do not** meet the definition of an AE include:

- Any clinically significant abnormal laboratory findings or other abnormal safety assessments that are associated with the underlying disease, unless judged by the investigator to be more severe than expected for the subject's condition.
- The disease/disorder being studied, or expected progression, signs, or symptoms of the disease/disorder being studied, unless more severe than expected for the subject's condition. (See Section 11.3, Events Common to ALS)
- Medical or surgical procedure (e.g., endoscopy, appendectomy); the condition that leads to the procedure is an AE.
- Situations where an untoward medical occurrence did not occur (social and/or convenience admission to a hospital).
- Anticipated day-to-day fluctuations of pre-existing disease(s) or condition(s) present or detected at the start of the study that do not worsen.

11.2. Definition of Serious Adverse Events

If an event is not an AE per Section 11.1, then it can not be an SAE even if serious conditions are met (e.g., hospitalization for signs/symptoms of the disease under study, death due to progression of disease, etc).

An SAE is any untoward medical occurrence that, at any dose:

a. Results in death

b. Is life-threatening

   NOTE: The term 'life-threatening' in the definition of 'serious' refers to an event in which the subject was at risk of death at the time of the event. It does not refer to an event, which hypothetically might have caused death, if it were more severe.

c. Requires hospitalization or prolongation of existing hospitalization

   NOTE: In general, hospitalization signifies that the subject has been detained (usually involving at least an overnight stay) at the hospital or emergency ward for observation and/or treatment that would not have been appropriate in the physician's office or out-patient setting. Complications that occur during hospitalization are AEs. If a complication prolongs hospitalization or fulfills any other serious criteria, the event is serious. When in doubt as to whether "hospitalization" occurred or was necessary, the AE should be considered serious.

   Hospitalization for elective treatment of a pre-existing condition that did not worsen from baseline is not considered an AE.

d. Results in disability/incapacity, or

   NOTE: The term disability means a substantial disruption of a person's ability to conduct normal life functions. This definition is not intended to include experiences of relatively minor medical significance such as uncomplicated headache, nausea, vomiting, diarrhea, influenza, and accidental trauma (e.g. sprained ankle) which may interfere or prevent everyday life functions but do not constitute a substantial disruption.

e. Is a congenital anomaly/birth defect

Medical or scientific judgment should be exercised in deciding whether reporting is appropriate in other situations, such as important medical events that may not be immediately life-threatening or result in death or hospitalization but may jeopardize the subject or may require medical or surgical intervention to prevent one of the other outcomes listed in the above definition. These should also be considered serious. Examples of such events are invasive or malignant cancers, intensive treatment in an emergency room or at home for allergic bronchospasm, blood dyscrasias or convulsions that do not result in hospitalization, or development of drug dependency or drug abuse.

11.2.1. Clinical Laboratory Abnormalities and Other Abnormal Assessments as AEs and SAEs

Abnormal laboratory findings (e.g., clinical chemistry, haematology, urinalysis) or other abnormal assessments (e.g., ECGs, vital signs) that are judged by the investigator as **clinically significant** will be recorded as AEs or SAEs if they meet the definition of an AE or SAE. Clinically significant abnormal laboratory findings or other abnormal assessments that are detected during the study or are present at baseline and significantly worsen following the start of the study will be reported as AEs or SAEs. However, clinically significant abnormal laboratory findings or other abnormal assessments that are associated with the disease being studied, unless judged by the investigator as more severe than expected for the subject's condition, or that are present or detected at the start of the study and do not worsen, will **not** be reported as AEs or SAEs.

The investigator will exercise his or her medical and scientific judgment in deciding whether an abnormal laboratory finding or other abnormal assessment is clinically significant.

11.3. Events Common to ALS

These events (see Appendix 3) commonly occur in subjects with ALS and are generally associated with the underlying ALS disease or progression of ALS. These events are considered disease-related and do not need to be reported as an AE/SAE unless the event is more severe than would normally be expected for a subject or if the investigator considers there to be a causal relationship between Investigational Product and the event. These predefined events are recorded on the Events Common to ALS eCRF page. Recording of events in this manner standardizes collection of common disease-related events and helps collection of events that might otherwise not be captured.

Death due to any of the events in the predefined list is to be recorded on the Record of Death eCRF page and not as an SAE, unless the event is more severe than would normally be expected for a subject or if the investigator considers there to be a causal relationship between Investigational Product and the event.

11.4. Method of Detecting AEs and SAEs

Care will be taken not to introduce bias when detecting AEs and/or SAEs. Open-ended and non-leading verbal questioning of the subject is the preferred method to inquire about AE occurrence. Appropriate questions include:

- "How are you feeling?"
- "Have you had any (other) medical problems since your last visit/contact?"
- "Have you taken any new medicines, other than those provided in this study, since your last visit/contact?"

11.5. Recording of AEs and SAEs

When an AE/SAE occurs, it is the responsibility of the investigator to review all documentation (e.g., hospital progress notes, laboratory, and diagnostics reports) relative to the event. The investigator will then record all relevant information regarding an AE/SAE in the appropriate data collection tool.

It is not acceptable for the investigator to send photocopies of the subject's medical records to GSK in lieu of completion of the GSK, AE/SAE data collection tool. However, there may be instances when copies of medical records for certain cases are requested by GSK. In this instance, all subject identifiers, with the exception of the subject number, will be blinded on the copies of the medical records prior to submission of to GSK.

The investigator will attempt to establish a diagnosis of the event based on signs, symptoms, and/or other clinical information. In such cases, the diagnosis will be documented as the AE/SAE and not the individual signs/symptoms.

11.6. Evaluating AEs and SAEs

11.6.1. Assessment of Intensity

The investigator will make an assessment of intensity for each AE and SAE reported during the study and will assign it to one of the following categories:

Mild: An event that is easily tolerated by the subject, causing minimal discomfort and not interfering with everyday activities.

Moderate: An event that is sufficiently discomforting to interfere with normal everyday activities.

Severe: An event that prevents normal everyday activities.

An AE that is assessed as severe will not be confused with an SAE. Severity is a category utilized for rating the intensity of an event; and both AEs and SAEs can be assessed as severe. An event is defined as 'serious' when it meets at least one of the pre-defined outcomes as described in the definition of an SAE.

11.6.2. Assessment of Causality

The investigator is obligated to assess the relationship between investigational product and the occurrence of each AE/SAE. A "reasonable possibility" is meant to convey that there are facts/evidence or arguments to suggest a causal relationship, rather than a relationship cannot be ruled out. The investigator will use clinical judgment to determine the relationship. Alternative causes, such as natural history of the underlying diseases, concomitant therapy, other risk factors, and the temporal relationship of the event to the investigational product will be considered and investigated. The investigator will also consult the Investigator Brochure (IB) and/or Product Information, for marketed products, in the determination of his/her assessment.

There may be situations when an SAE has occurred and the investigator has minimal information to include in the initial report to GSK. However, **it is very important that the investigator always make an assessment of causality for every event prior to the initial transmission of the SAE data to GSK.** The investigator may change his/her opinion of causality in light of follow-up information, amending the SAE data collection tool accordingly. The causality assessment is one of the criteria used when determining regulatory reporting requirements.

The investigator will provide the assessment of causality as per the instructions for completion of the AE/SAE data collection tool.

11.7. Follow-up of AEs and SAEs

After the initial AE/SAE report, the investigator is required to proactively follow each subject at subsequent visits/contacts. All AEs and SAEs will be followed until resolution, until the condition stabilizes, until the event is otherwise explained, or until the subject is lost to follow-up.

The investigator is obligated to perform or arrange for the conduct of supplemental measurements and/or evaluations as may be indicated or as requested by GSK to elucidate as fully as possible the nature and/or causality of the AE or SAE. The investigator is obligated to assist. This may include additional laboratory tests or investigations, histopathological examinations or consultation with other health care professionals. If a subject dies during participation in the study or during a recognized follow-up period, the investigator will provide GSK with a copy of any post-mortem findings, including histopathology.

New or updated information will be recorded in the originally completed data collection tool. The investigator will submit any updated SAE data to GSK within the designated reporting time frames.

11.8. Prompt Reporting of SAEs to GSK

Once the investigator determines that an event meets the protocol definition of an SAE, the SAE will be reported to GSK **within 24 hours**. Any follow-up information on a previously reported SAE will also be reported to GSK within 24 hours.

If the investigator does not have all information regarding an SAE, he/she will not wait to receive additional information before notifying GSK of the event and completing the appropriate data collection tool. The investigator will always provide an assessment of causality at the time of the initial report as described in Section 11.6.2, Assessment of Causality.

The primary mechanism for reporting SAEs to GSK will be the electronic data collection tool (e.g., InForm system). If the electronic system is unavailable for greater than 24 hours, the site will use the paper SAE data collection tool and fax it to the GSK Medical Monitors at +44 208 043 1489 (UK). Then the site will enter the serious adverse event data into the electronic system as soon as it becomes available.

The Sponsor's Medical Monitor shall ensure that any SAE report received from one site, will be notified to all participating Investigators of all other active sites immediately and within 24 hours at the latest after being received by GSK. This notification to other Investigators will be made by email and a follow-up telephone call to ensure that they have received and read the email.

After the study is completed at a given site, the electronic data collection tool (e.g., InForm system) will be taken off-line to prevent the entry of new data or changes to existing data. If a site receives a report of a new SAE from a study participant or receives updated data on a previously reported SAE after the electronic data collection tool has been taken off-line, the site can report this information on a paper SAE form or to their GSK protocol contact by telephone.

GSK contacts for SAE receipt can be found at the beginning of this protocol on the Sponsor/Medical Monitor Contact Information page.

11.9. Regulatory Reporting Requirements For SAEs

Prompt notification of SAEs by the investigator to GSK is essential so that legal obligations and ethical responsibilities towards the safety of subjects are met.

GSK has a legal responsibility to notify both the local regulatory authority and other regulatory agencies about the safety of a product under clinical investigation. GSK will comply with applicable local and country specific regulatory requirements relating to safety reporting and ensure that all the relevant information about suspected unexpected serious adverse reactions and SAEs are recorded and reported to the competent regulatory authorities, IRBs/IECs and investigators according to the European Directive and FDA guidelines.

Investigator safety reports are prepared for suspected unexpected serious adverse reactions according to local regulatory requirements and GSK policy and are forwarded to investigators as necessary. An investigator who receives an investigator safety report describing an SAE(s) or other specific safety information (e.g., summary or listing of SAEs) from GSK will file it with the IB and will notify the IRB/IEC, if appropriate according to local requirements.

12. LIVER CHEMISTRY TESTING PROCEDURES

The procedures listed below are to be followed if a subject meets any of the liver chemistry stopping criteria defined in Section 3.3.2:

- Notify the GSK medical monitor within 24 hours of learning of the abnormality to confirm the subject's investigational product cessation and follow-up.
- Complete the "Safety Follow-Up Procedures" listed below.
- Complete the liver event case report forms. If the event also meets the criteria of an SAE (see Section 11.2), the SAE data collection tool will be completed separately with the relevant details.

- Upon completion of the safety follow-up do not rechallenge with investigational product.

Safety Follow-Up Procedures for subjects with ALT ≥3xULN and bilirubin ≥2xULN:

- Make every reasonable attempt to have the subjects return to the clinic (within 24 hours) for repeat liver chemistries, additional testing and to be monitored closely (with specialist or hepatology consultation recommended).
- Monitor subjects twice weekly until liver chemistries (ALT, AST, alkaline phosphatase, bilirubin) resolve, stabilize or return to within baseline values.

Safety Follow-Up Procedures for subjects with ALT ≥ 5xULN or ALT ≥ 3xULN with hepatitis or hypersensitivity:

- Make every reasonable attempt to have the subject return to the clinic within 24-72 hrs for repeat liver chemistries and additional testing.
- Monitor subjects weekly until liver chemistries (ALT, AST, alkaline phosphatase, bilirubin) resolve, stabilize or return to within baseline values.

Additional Follow-Up Procedures for subjects who meet *any* of the stopping criteria:

- Viral hepatitis serology including:
  - Hepatitis A IgM antibody;
  - Hepatitis B surface antigen and Hepatitis B Core Antibody (IgM);
  - Hepatitis C RNA;
  - Cytomegalovirus IgM antibody;
  - Epstein-Barr viral capsid antigen IgM antibody (or if unavailable, obtain heterophile antibody or monospot testing);
  - Hepatitis E IgM antibody (if subject resides outside the USA or Canada, or has traveled outside USA or Canada in past 3 months)
- Blood sample for pharmacokinetic (PK) analysis, obtained within 24 hours of observation and 7-days thereafter. Record the date/time of the PK blood sample draw and the date/time of the last dose of investigational product prior to blood sample draw on the CRF. Instructions for sample handling and shipping are included in the SPM.
- Serum creatine phosphokinase (CPK) and lactate dehydrogenase (LDH).
- Fractionate bilirubin, if total bilirubin ≥ 2xULN.
- Assess eosinophilia
- Record the appearance or worsening of clinical symptoms of hepatitis, or hypersensitivity, fatigue, decreased appetite, nausea, vomiting, abdominal pain, jaundice, fever, or rash as relevant on the AE CRF

- Record use of concomitant medications, acetaminophen, herbal remedies, other over the counter medications, or putative hepatotoxins on the Concomitant Medications CRF.
- Record alcohol use on the Liver Events CRF.

The following are required for subjects with ALT ≥ 3xULN **and** bilirubin ≥ 2xULN but are optional for other abnormal liver chemistries:

- Anti-nuclear antibody, anti-smooth muscle antibody, and Type 1 anti-liver kidney microsomal antibodies.
- Liver imaging (ultrasound, magnetic resonance, or computerized tomography) to evaluate liver disease.
- The Liver Imaging and/or Liver Biopsy CRFs are also to be completed if these tests are performed.

The procedures listed below are to be followed if a subject meets the criterion of ALT ≥3xULN and <5xULN and bilirubin <2xULN, but does not exhibit hepatitis symptoms or rash:

- Notify the GSK medical monitor within 24 hours of learning of abnormality to discuss subject safety
- If the subject can be monitored weekly for 4 weeks, continue investigational product and collect liver chemistries within 1 week. Continue weekly collection of liver chemistries up to 4 weeks. If the ALT remains 3xULN after 4 weeks, continue to monitor the liver chemistries weekly until values resolve, stabilize or return to baseline values. If ALT <3xULN at 4 weeks, continue to monitor liver chemistry twice monthly until values normalize or return to baseline.
- If the subject cannot be monitored weekly for 4 weeks, stop investigational product and collect liver chemistries within 1 week. Attempt to collect subsequent liver chemistries weekly if possible, but at a minimum, collection should be twice per month until values resolve, stabilize or return to within baseline values.

13. STUDY CONDUCT CONSIDERATIONS

13.1. Regulatory and Ethical Considerations, Including the Informed Consent Process

GSK will obtain favorable opinion/approval to conduct the study from the appropriate regulatory agency in accordance with any applicable country-specific regulatory requirements prior to a site initiating the study in that country.

The study will be conducted in accordance with all applicable regulatory requirements,

The study will also be conducted in accordance with "good clinical practice" (GCP), all applicable subject privacy requirements, and, the guiding principles of the Declaration of Helsinki Version 2004 This includes, but is not limited to, the following:

- IRB/IEC review and favorable opinion/approval to conduct the study and of any subsequent relevant amended documents
- Written informed consent (and any amendments) to be obtained for each subject before participation in the study
- Investigator reporting requirements (e.g. reporting of AEs/SAEs/protocol deviations to IRB/IEC)

GSK will provide full details of the above either verbally, in writing or both.

Written informed consent will be obtained for each subject before he or she can participate in the study.

GSK R&D as sponsors of this study will comply with the local applicable regulatory requirements and ensure that all the relevant information about adverse drug reactions and SAEs are recorded and reported to the competent authority according the FDA guidelines and European Directive 2001/20/EC

Urgent Safety Measures

If an event occurs that is related to the conduct of the study or the development of the investigational product, and this new event is likely to affect the safety of subjects, the sponsor and the investigator will take appropriate urgent safety measures to protect subjects against any immediate hazard.

The sponsor will work with the investigator to ensure the IEC/IRB is notified.

13.2. Quality Control (Study Monitoring)

In accordance with applicable regulations including GCP, and GSK procedures, GSK monitors will contact the site prior to the start of the study to review with the site staff the protocol, study requirements, and their responsibilities to satisfy regulatory, ethical, and GSK requirements. When reviewing data collection procedures, the discussion will also include identification, agreement and documentation of data items for which the source records will be identified.

GSK will monitor the study and site activity to verify that the:

- Data are authentic, accurate, and complete.
- Safety and rights of subjects are being protected.
- Study is conducted in accordance with the currently approved protocol and any other study agreements, GCP, and all applicable regulatory requirements.

The investigator and the head of the medical institution (where applicable) agrees to allow the monitor direct access to all relevant documents

13.3. Quality Assurance

To ensure compliance with GCP and all applicable regulatory requirements, GSK may conduct a quality assurance audit. Regulatory agencies may also conduct a regulatory inspection of this study. Such audits/inspections can occur at any time during or after completion of the study. If an audit or inspection occurs, the investigator and institution agree to allow the auditor/inspector direct access to all relevant documents and to allocate his/her time and the time of his/her staff to the auditor/inspector to discuss findings and any relevant issues.

13.4. Study and Site Closure

Upon completion or premature discontinuation of the study, the monitor will conduct site closure activities with the investigator or site staff, as appropriate, in accordance with applicable regulations including GCP, and GSK procedures.

In addition, GSK reserves the right to temporarily suspend or prematurely discontinue this study at any time for reasons including, but not limited to, safety or ethical issues or severe non-compliance. For multicentre studies, this can occur at one or more or at all sites. If GSK determines such action is needed, GSK will discuss this with the investigator or the head of the medical institution (where applicable), including the reasons for taking such action. When feasible, GSK will provide advance notification to the investigator or the head of the medical institution, where applicable, of the impending action prior to it taking effect.

If the study is suspended or prematurely discontinued for safety reasons, GSK will promptly inform investigators or the head of the medical institution (where applicable) and the regulatory authorities of the suspension or premature discontinuation of the study and the reason(s) for the action. If required by applicable regulations, the investigator or the head of the medical institution (where applicable) must inform the IRB/IEC promptly and provide the reason for the suspension or premature discontinuation.

13.5. Records Retention

Following closure of the study, the investigator or the head of the medical institution (where applicable) must maintain all site study records, except for those required by local regulations to be maintained by someone else, in a safe and secure location. The records must be maintained to allow easy and timely retrieval, when needed (e.g., audit or inspection), and, whenever feasible, to allow any subsequent review of data in conjunction with assessment of the facility, supporting systems, and staff. Where permitted by local laws/regulations or institutional policy, some or all of these records can be maintained in a format other than hard copy (e.g., microfiche, scanned, electronic); however, caution needs to be exercised before such action is taken. The investigator must assure that all reproductions are legible and are a true and accurate copy of the original, and meet accessibility and retrieval standards, including re-generating a hard copy, if required. Furthermore, the investigator must ensure there is an acceptable back-up of these reproductions and that an acceptable quality control process exists for making these reproductions.

GSK will inform the investigator of the time period for retaining these records to comply with all applicable regulatory requirements. The minimum retention time will meet the strictest standard applicable to that site for the study, as dictated by any institutional requirements or local laws or regulations, or GSK standards/procedures; otherwise, the retention period will default to 15 years.

The investigator must notify GSK of any changes in the archival arrangements, including, but not limited to, archival at an off-site facility or transfer of ownership of the records in the event the investigator leaves the site.

13.6. Provision of Study Results and Information to Investigators

When required by applicable regulations, the investigator signatory for the clinical study report will be determined at the time the report is written. When the clinical study report is completed, GSK will provide the investigator with a full summary of the study results. The investigator is encouraged to share the summary results with the subjects, as appropriate. In addition, the investigator will be given reasonable access to review the relevant statistical tables, figures, and reports and will be able to review the results for the entire study at a GSK site or other mutually agreeable location.

GSK will provide the investigator with the randomization codes for their site after the statistical analysis for the entire study has been completed.

13.7. Data Management

GSK Data Management will identify and implement the most effective data acquisition and management strategy for each clinical trial protocol and deliver datasets which support the protocol objectives. Subject data will be entered into GSK defined CRFs and combined with data provided from other sources (e.g. diary data, laboratory data) in a validated data system. Clinical data management will be performed in accordance with applicable GSK standards and data cleaning procedures with the objective of removing errors and inconsistencies in the data which would otherwise impact on the analysis and reporting objectives, or the credibility of the Clinical Study Report. Adverse events and concomitant medications terms will be coded using validated dictionaries. Original CRFs will be retained by GSK, while the investigator will retain a copy. In all cases, subject initials will not be collected nor transmitted to GSK.

14. REFERENCES

Andersen PM, Borasio GD, Dengler R, Hardiman O, Kollewe K, Leigh PN, Pradat PF, Silani V, Tomik B. EFNS task force on management of amyotrophic lateral sclerosis: guidelines for diagnosing and clinical care of patients and relatives. *Eur J Neurol.* 2005 ;12(12): 921-38.

Andersen PM. The genetics of amyotrophic lateral sclerosis (ALS). *Suppl Clin Neurophysiol*. 2004; 57: 211-27.

Bensimon G, Lacomblez L, Meininger V, McKee P, Fuller GN, Stevens GL, Macrae KD, Burgerman RS, Rogawski MA, Murphy JR, Rowland LP, Caillard CG, Louvel E, Randle JCR. A Controlled Trial of Riluzole In Amyotrophic Lateral Sclerosis. *N Engl J Med.* 1994 ; 330 :585-91.

Brett SJ, Rowan W, Smith M, Bartholomew M, Tite JP. Differential functional effects of a humanized anti-CD4 antibody on resting and activated human T cells. *Immunology, 1997*;91:346-353.

Bruijn LI, Becher MW, Lee MK, Anderson KL, Jenkins NA, Copeland NG, Sisodia SS, Rothstein JD, Borchelt DR, Price DL, Cleveland DW. ALS- Linked SOD1 Mutant G85R Mediates Damage to Astrocytes and Promotes Rapidly Progressive Disease with SOD1-Containing Inclusions. *Neuron.* 2004; 18: 327-338

ClinicalTrials.gov Identifier: NCT00406016, 2007. A Multi-Center, Open-Label, Cohort Study to Assess Feasibility, Acute Safety, Tolerability and Pharmacokinetics of 4 Dose Regimens of Continuous Intrathecal ATI355 Infusion in Acute Spinal Cord Injury Paraplegic Patients and Tetraplegic Patients

Dupuis L, Gonzalez de Aguilar JLG, Scala F, Rene F, Tapia M, Pradat PF, Lacomblez L, Seihlan D, Prinjha R, Walsh F, Meininger V, Loeffler JP. Nogo Provides a Molecular Marker for Diagnosis of Amyotrophic lateral Sclerosis. *Neurobiology of Disease.* 2002 ; 3 :358-365

Enayat ZE, Orrell RW, Claus A, Ludolph , Bachus R, Brockmuller J, Ray-Chaudhuri K, Radunovic A, Shaw C, Wilkinson J, King A, Swash M, Leigh PN, Belleroche JD, Powell J. Two novel mutations in the gene for copper zinc superoxide dismutase in UK families with amyotrophic lateral sclerosis. *Human Molecular Genetics.* 1995;(4): 1239-1240

Fuller C, Cedarbaum JM, Stambler N, Malta E, Hilt D, Thurmond B, Nakanishi A. The ALSFRS-R: a revised ALS functional rating scale that incorporates assessments of respiratory function. *Journal of the Neurological Sciences*. 1999 ;169 : 13-21

GlaxoSmithKline Document Number GSK07DMF020.

Gruzman A, Wood WL, Alpert E, Prasad MD, Miller RG, Rothstein JD, Bowser R, Hamilton R, Wood TD, Cleveland DW, Lingappa VR, Liu J. (2007) Common molecular signature in SOD1 for both sporadic and familial amyotrophic lateral sclerosis. *Proc Natl Acad Sci U S A.* 104(30):12524-9.

Gurney M. E., Pu H., Chiu A. Y. et al. (1994) Motor neuron degeneration in mice that express a human Cu,Zn superoxide dismutase mutation. *Science.* 264; 1772–1775.

Jokic N, Gonzalez de Aguilar JLG, Pradat PF, Dupuis L, Laguna AE, Muller A, Dubourg O, Seilhean D, Hauw JJ, Loeffler JP, Meininger V. Nogo expression in muscle correlates with amyotrophic lateral sclerosis severity. *Annals of Neurology.* 2005; 57(4): 553-556

Jokic N, Gonzalez de Aguilar JLG, Dimou L, Lin S, Fergani A, Ruegg MA, Schwab ME, Dupuis L, Loeffler JP. The neurite outgrowth inhibitor Nogo-A promotes denervation in an amyotrophic lateral sclerosis model. *EMBO*. 2006; 11:1162-1167

Jusko W. Antibody Pharmacokinetics: Oral presentation AAPS National Biotechnology Conference. 2005. Vol 7, Issue S1.

Kabat EA, Qu TT, Perry HM, Gottesman KS, Foeller C. Sequences of Proteins of Immunological Interest, US Department of Health and Human Services, Public Health Service, National Institutes of Health, 1991, Fifth Ed

Kim JE, Li S, GrandPre T, Qiu D, Strittmatter SM. Axon regeneration in young adult mice lacking Nogo-A/B. Neuron, 2003, 38(2):187-99.

Lacomblez L, Bensimon G, Leigh PN, Guillet P, Meininger V. Dose ranging study of riluzole in amyotrophic lateral sclerosis. *Lancet.* 1996 ; 3 ;348(9023) :336-7.

Lobo et al, Antibody Pharmacokinetics and Pharmacodynamics. *Journal of Pharmaceutical Sciences*. 2005. 93(11)

Miller RG, Voltz R, Borasio GD. Palliative care in amyotrophic lateral sclerosis. *Neurologic Clinics.* 2007;19(4): 829-847

Mordenti, J., Chen, S. A., Moore, J. A., Ferraiolo, B. L. and Green, J. D. Interspecies scaling of clearance and volume of distribution data for five therapeutic proteins. *Pharmaceutical Research*, 1991;8, 1351-1359 et al *Pharmaceutical Research*.

Mulder DW, Kurland LT, Offord KP, Beard CM. Familial adult motor neuron disease: amyotrophic lateral sclerosis. *Neurology*. 1986;36:511-517

Pradat PF, Bruneteau G, Gonzalez de Aguilar JL, Dupuis L, Jokic N, Salachas F, Le Forestier N, Echaniz-Laguna A, Dubourg O, Hauw JJ, Tranchant C, Loeffler JP, Meininger V. Muscle Nogo-A expression is a prognostic marker in lower motor neuron syndromes. *Annals of Neurology.* 2007;62(1): 15-20

Pernet V, Joly S, Christ F, Dimou L, Schwab ME (2008). Nogo-A and Myelin-Associated Glycoprotein Diferentially Regulate Oligodendrocyte Maturation and Myelin Formation. *J. Neuroscience*, 2008, 28 (29), 7435-44.

Reaume A. G., Elliott J. L., Hoffman E. K. et al. (1996) Motor neurons in Cu/Zn superoxide dismutase-deficient mice develop normally but exhibit enhanced cell death after axonal injury. *Nat. Genet.* 13; 43–47

Rix Brooks B, Miller RG, Swash M, Munsat TL. El Escorial revisited: Revised criteria for the diagnosis of amyotrophic lateral sclerosis. Journal of ALS and other motor neuron diseases, 2000, 1 (5), 293-299

Rosen DR, Siddique T, Patterson D, Figlewicz DA, Sapp P, Hentati A, Donaldson D,Goto J, O'Regan JP, Deng HX. Mutations in Cu/Zn superoxide dismutase gene are associated with familial amotrophic lateral sclerosis. *Nature.* 1993; 362:59–62.

Simonen M, Pedersen V, Weinmann O, Schnell L, Buss A, Ledermann B, Christ F, Sansig G, van der Putten H, Schwab ME. Systemic deletion of the myelin-associated outgrowth inhibitor Nogo-A improves regenerative and plastic responses after spinal cord injury. Neuron, 2003,38(2):201-11.

Tabrizi M, Tseng C, Roskos L. Elimination mechanisms of therapeutic monoclonalantibodies. *Drug Discovery Today.* 2006. 11: 81-88.

Walmsley AR, Mir AK. (2007). Targeting the Nogo-A Signalling Pathway to Promote Recovery Following Acute CNS Injury. *Current Pharm Des.*, 2007, 13 (24), 2470-84

Zheng B, Ho C, Li S, Keirstead H, Steward O, Tessier-Lavigne M. Lack of enhanced spinal regeneration in Nogo-deficient mice. Neuron, 2003, 38 (2) 213-224

Appendices

Appendix 1: Pharmacogenetic research

Pharmacogenetics - Background

Pharmacogenetics (PGx) is the study of variability in drug response due to hereditary factors in different populations. There is increasing evidence that an individual's genetic composition (i.e., genotype) may impact the pharmacokinetics (absorption, distribution, metabolism, elimination), pharmacodynamics (relationship between concentrations and pharmacologic effects or the time course of pharmacologic effects) and/or clinical outcome (in terms of efficacy and/or safety and tolerability). Collection of whole blood samples, even when no a prior hypothesis has been identified, may enable PGx analysis to be conducted if at any time it appears that there is a potential unexpected or unexplained variation in handling or response to GSK1223249.

Pharmacogenetic Research Objectives

The objective of the PGx research (if there is a potential unexpected or unexplained variation) is to investigate a possible genetic relationship to handling or response to GSK1223249. If at any time it appears there is potential variability in response in this clinical study or in a series of clinical studies with GSK1223249 that may be attributable to genetic variations of subjects, the following objectives may be investigated:

- Relationship between genetic variants and the pharmacokinetics of investigational product
- Relationship between genetic variants and safety and/or tolerability of investigational product
- Relationship between genetic variants and efficacy of investigational product

Informed Consent

Subjects who do not wish to participate in the PGx research may still participate in the clinical study. PGx informed consent must be obtained prior to any blood being taken for PGx research. Refusal to participate will involve no penalty or loss of benefits to which the subject would otherwise be entitled.

Study Population

Any subject who has given informed consent to participate in the clinical study, has met all the entry criteria for the clinical study, and receives investigational product may take part in the PGx research provided the subject has given consent to the specific collection of a PGx sample. Any subject who has received an allogeneic bone marrow transplant must be excluded from the PGx research.

Subject participation in the PGx research is voluntary and refusal to participate will not indicate withdrawal from the clinical study.

Study Assessments and Procedures

In addition to any blood samples taken for the clinical study, a whole blood sample (~10ml) will be collected for the PGx research using a tube containing EDTA. The PGx sample is labelled (or "coded") with a study specific number that can be traced or linked back to the subject by the investigator or site staff. Coded samples do not carry personal identifiers (such as name or social security number). The blood sample will be taken on a single occasion, unless a duplicate sample is required due to inability to use the original sample. It is recommended that the blood sample be taken at the first opportunity after a subject has been randomized and provided informed consent for PGx research, but the sample may be taken at any time during the subject's participation in the clinical study.

If deoxyribonucleic acid (DNA) is extracted from the blood sample, the DNA may be subjected to sample quality control analysis. This analysis will involve the genotyping of several genetic markers to confirm the integrity of individual samples. If inconsistencies are noted in the analysis, then those samples may be destroyed.

The need to conduct PGx analysis may be identified after a study (or a set of studies) of GSK1223249 has been completed and the study data reviewed. For this reason, samples may be kept for up to 15 years after the last subject completes the study or GSK may destroy the samples sooner. In special cases, the samples may not be studied, e.g., if there are not enough subjects, if the study is stopped for other reasons, or if no questions are raised about how people respond to GSK1223249. GSK or those working with GSK (for example, other researchers) will only work with samples collected from the study for the use stated in this protocol and in the informed consent form. Samples will be stored securely. Subjects can request their sample to be destroyed at any time.

Subject Withdrawal from Study

If a subject who has consented to participate in PGx research withdraws from the clinical study for any reason other than being lost to follow-up, the subject will be given a choice of one of the following options concerning the PGx sample, if already collected:

- PGx research continues per the subject's consent (i.e., the sample is retained); or,
- Any remaining sample is destroyed

If a subject withdraws consent from the PGx research or requests sample destruction, the investigator must request sample destruction by completing the appropriate documentation within the specified timeframe specified, and maintain the documentation in the site study records. In either case, GSK will only keep study information collected/generated up to that point.

Screen and Baseline Failures

If a blood sample for PGx research has been collected and it is then determined that the subject does not meet the entry criteria for participation in the clinical study, then the investigator must request sample destruction by completing the appropriate documentation within the specified timeframe, and maintain the documentation in the site study records.

Pharmacogenetics Analyses

The need to conduct PGx analysis may be identified after a study (or set of studies) has been completed. For this reason, samples may be kept for up to 15 years after the last subject completes the study. GSK may destroy the samples sooner.

Generally, GSK will utilize any of three approaches to explore genetic variation in drug response.

1. Specific genetic markers may be selected from “candidate genes” known to encode the drug target, drug metabolizing enzymes, molecules associated with mechanisms underlying adverse events (for example, molecules important for immune response), and those linked to drug response. Candidate genes that may be investigated in this study are genes from the GSK Absorption, Distribution, Metabolism and Excretion (ADME) panel. ADME genes play a central role in drug pharmacokinetics and pharmacodynamics (PK-PD). The GSK ADME panel contains genetic markers from one hundred and thirty-five enzymes, transporters and other genes involved in drug absorption, distribution, metabolism and excretion. The ADME panel may be used to investigate the relationship between genetic variants on the panel and pharmacokinetics, safety and efficacy of the investigational product.

In addition, continuing research may identify other enzymes, transporters, proteins, or receptors that may be involved in response to GSK1223249. The genes that may code for these proteins may also be studied.

2. Evaluate markers that comprise pre-defined “panels” for association with specified endpoints.

Examples of such panels include the GSK ADME (Absorption, Distribution, Metabolism, and Excretion) Panel and the GSK DILI (Drug Induced Liver Injury) Panel which consist of genetic markers from set of genes that are known to be related to pharmacokinetic, pharmacodynamic, immune, or adverse drug response.

3. Evaluate markers throughout the genome using a whole genome screen (WGS).

By evaluating large numbers of genetic markers (e.g., single nucleotide polymorphisms or SNPs) throughout the genome, sets of markers may be identified that correspond to differential drug response.

In all cases, appropriate statistical methods will be used to analyze the genetic markers in the context of other clinical data. The statistical methods for analysis may include, but are not limited to Hardy-Weinberg Equilibrium (HWE) Analysis, Linkage Disequilibrium Analysis, Evaluation of Genotypic Effects, Evaluation of Treatment by Genotype and Gene-Gene Interaction, Multiple Comparisons and Multiplicity, and/or Power and Sample Size Considerations. Detailed description of all analyses to be conducted will be documented in the Reporting and Analysis Plan.

Provision of Study Results and Confidentiality of Subject's PGx Data

GSK may summarize the cumulative PGx research results in the clinical study report.

In general, GSK will not inform the investigator, subject, or anyone else (e.g., family members, study investigators, primary care physicians, insurers, or employers) of the PGx research results under any circumstances unless required by law. This is because the information generated from PGx studies is preliminary in nature, and the significance and scientific validity of the results are undetermined at such an early stage of research.

References

Cardon LR, Idury RM, Harris TJR, Witte JS, Elston RC. Testing drug response in the presence of genetic information: sampling issues for clinical trials. *Pharmacogenetics*. 2000; 10:503-10.

McCarthy JJ, Hilfiker R. The use of single-nucleotide polymorphism maps in pharmacogenomics. *Nat Biotechnol*. 2000; 18:505-8.

Palmer LJ, Cookson WO. Using single nucleotide polymorphisms as a means to understanding the pathophysiology of asthma. *Respir Res*. 2001; 2:102-12.

Appendix 2: Country Specific Requirements

Additional Inclusion Criteria for Patients in France:

"In France, a subject will be eligible for inclusion in this study only if either affiliated to or a beneficiary of a social security category."

Appendix 3: Events common to ALS

- cognitive impairment/fronto-temporal dementia
- constipation
- contractures/cramping/fasiculations
- deep vein thrombosis
- depression or other mood disorders (e.g., emotional lability, anxiety, etc)
- dysarthria
- dysphagia
- dyspnea
- edema – limb
- fatigue
- frozen shoulder
- mucous/airway secretion/obstruction
- hyperreflexia
- muscle atrophy/wasting
- musculoskeletal pain
- orthopnea (dyspnea/orthopnea)
- peudobulbar syndrome (A syndrome characterized by dysarthria, dysphagia, dysphonia, impairment of voluntary movements of tongue and facial muscles, and emotional lability)
- peripheral cyanosis
- pulmonary embolism
- pneumonia
- sialorrhea
- skin breakdown/skin ulcers
- sleep disorders
- spasticity
- weakness
- weight loss

Appendix 4: Protocol Amendment Changes

AMENDMENT 4

Where the Amendment Applies

This amendment applies to all countries and sites.

Summary of Amendment Changes with Rationale

To increase the dose in part 2, (cohort 8) from 7.5mg/kg to 15mg/kg based on emerging safety and tolerability data to thoroughly explore safety and tolerability of GSK1223249 at dose levels thought feasible to be taken forward into future studies.

For cohort 8: To change the post-dose muscle biopsy collection such that post-dose muscle biopsies are obtained at different intervals following the first dose (each subject still to have one post-dose muscle biopsy at either Day 1(+24hrs), Day 8 or Week 4 (Day 22-28) to better characterize target-organ (muscle) pharmacokinetics.

To add clarity to Table 6 showing cohort 5 and 6 starting in parallel and cohort 8 post dose muscle biopsy scheduled before dose 2.

Section 1.2.2 Pre-Clinical Pharmacology, Section 1.2.3 Pre-Clinical Safety, Section 1.2.4 Pre-Clinical Pharmacokinetics and Section 1.3.2 Dose Rationale updated with new data.

To add clarity to the 1hr post dose PK sample collection time to ensure the sample is collected at the end of the infusion.

Clarification for inclusion criteria 4 and in Section 8.1 that patients should be on stable medications within 28 days prior to dosing.

Additional authors added

Minor typographic corrections.

List of Specific Changes

Title Page

Description

PREVIOUS TEXT

In two cohorts in Part 1 and all cohorts in Part 2, blood samples and skeletal muscle biopsies will be taken from patients before and at the end of treatment to demonstrate whether or not GSK1223249 binds to its target and produces any measurable pharmacodynamic effect.

REVISED TEXT

In two cohorts in Part 1 and all cohorts in Part 2, blood samples and skeletal muscle biopsies will be taken from patients before and **<u>at specific times</u>** ~~the end of treatment~~ **<u>post dosing</u>** to demonstrate whether or not GSK1223249 binds to its target and produces any measurable pharmacodynamic effect.

Title Page

Subject

PREVIOUS TEXT

Subject: FTIH Study, GSK1223249, ALS, Single and Repeat Dose Escalation, Safety, Pharmacokinetics, Pharmacodynamics, Fusion protocol

REVISED TEXT

Subject: FTIH Study, GSK1223249, ALS, Single and Repeat Dose Escalation, Safety, Pharmacokinetics, Pharmacodynamics, Fusion protocol**<u>, Muscle Biopsies.</u>**

Title Page

Author

PREVIOUS TEXT

Author: [redacted]

REVISED TEXT

Author: [redacted]

Section 1.2.2 Pre-Clinical Pharmacology

Species Orthologue binding

PEVIOUS TEXT

The binding kinetics for rat, cynomolgus monkey, squirrel monkey and marmoset are very similar to that for human (range = 0.33-0.67nM).

REVISED TEXT

The binding kinetics for rat, cynomolgus monkey, squirrel monkey ~~and~~, marmoset **and rabbit** are very similar to that for human (range = 0.33-0.67nM).

Section 1.2.2 Pre-Clinical Pharmacology

Species Ortologue binging

Table 1

PREVIOUS TEXT

Table 1 Binding kinetics of GSK1223249 to the recombinant orthologues of human Nogo-A

	GSK1223249		
Orthologue	*Ka*	*Kd*	KD (nM)
Cynomolgus (2 runs)	4.65e6 (7.47e5)	3.07e-3 (2.37e-4)	0.67 (0.06)
Rat (2 runs)	4.64e6 (2.34e5)	1.54e-3 (3.06e-5)	0.33 (0.01)
Marmoset (1 run)	4.2e6 (2.47e4)	3.02e-3 (5.09e-5)	0.626 (0.000)
Squirrel Monkey (1 run)	4.46e6 (6.08e4)	2.73e-3 (4.95e-6)	0.61 (0.000)
[1]Human	6.97e6 (6.62e5)	4.43e-3 (1.18e-3)	0.64 (0.15)

As determined using the Biacore T100. Approximately 140-180RUs of the various Nogo-A orthologues were captured to the CM5 chip by primary amine coupling. The antibody was flowed over at various concentrations (0.125-8nM). The values show the mean and standard deviation (in brackets) of 1-2 independent runs carried out in duplicate with each data set independently analysed prior to calculation of the mean and standard deviation.

1. For reference, the kinetics of binding to human GST-Nogo-A 5+6 is shown in the final row.

REVISED TEXT

Table 1 Binding kinetics of GSK1223249 to the recombinant orthologues of human Nogo-A

	GSK1223249		
Orthologue	*Ka*	*Kd*	KD (nM)
Cynomolgus (2 runs)	4.65e6 (7.47e5)	3.07e-3 (2.37e-4)	0.67 (0.06)
Rat (2 runs)	4.64e6 (2.34e5)	1.54e-3 (3.06e-5)	0.33 (0.01)
Marmoset (1 run)	4.2e6 (2.47e4)	3.02e-3 (5.09e-5)	0.626 (0.000)
Squirrel Monkey (1 run)	4.46e6 (6.08e4)	2.73e-3 (4.95e-6)	0.61 (0.000)
**Rabbit**	**4.202e6 (NA)**	**2.271e-3 (NA)**	**0.541 (NA)**
[1]Human	6.97e6 (6.62e5)	4.43e-3 (1.18e-3)	0.64 (0.15)

As determined using the Biacore T100. Approximately 140-180RUs of the various Nogo-A orthologues were captured to the CM5 chip by primary amine coupling. The antibody was flowed over at various concentrations (0.125-8nM). The values show the mean and standard deviation (in brackets) of 1-2 independent runs carried out in duplicate with each data set independently analysed prior to calculation of the mean and standard deviation.

1. For reference, the kinetics of binding to human GST-Nogo-A 5+6 is shown in the final row.

Section 1.2.3 Pre-Clinical Safety

Overview of the Non-Clinical Testing Strategy

PREVIOUS TEXT

The safety profile of GSK1223249 was evaluated in a range of repeat-dose studies, in Sprague Dawley rats and cynomolgus monkeys, in which GSK1223249 was dosed once every 2 weeks for a period of 12 weeks. An enhanced assessment of neurobehavioral function was performed as part of the 12-week repeat dose monkey study. A combined cardiovascular and respiratory safety pharmacology study was also performed in cynomolgus monkeys using single doses of GSK1223249. An in vitro flow cytometric cross-reactivity study to evaluate binding of GSK1223249 to cynomolgus monkey, human and rat peripheral blood has been completed. In vitro cross reactivity studies to evaluate the binding of GSK1223249 to a range of rat, cynomolgus monkey and human tissues have also been performed. Genetic toxicology studies have not been performed as biopharmaceuticals, such as mAbs, do not interact directly with DNA or chromosomes.

REVISED TEXT

The safety profile of GSK1223249 was evaluated in ~~a range of~~repeat dose studies ~~,in Spague Dawley rats and cynomolgus monkeys,~~ in which GSK1223249 was dosed once

every 2 weeks **in Sprague Dawley rats** for ~~a period up to~~ **up to** 12 weeks **and cynomolgus monkeys for up to 52 weeks in duration.** An enhanced assessment of neurobehavioral function was performed as part of the 12 week repeat dose monkey study. A combined cardiovascular and respiratory safety pharmacology study was also performed in cynomolgus monkeys using single **IV** doses of GSK1223249. **Female fertility and embryofetal studies have been conducted in Sprague Dawley rats and Dutch Belted rabbits, respectively.** An in vitro flow cytometric cross-reactivity study to evaluate binding of GSK1223249 to **rat**, cynomolgus monkey **and** human ~~and rat~~peripheralblood has been ~~completed. In vitro~~**conducted. Tissue** cross-reactivity studies to evaluate the binding ofGSK1223249 to a range of **tissues from** rat, cynomolgus monkey and human ~~tissues~~have also been performed. ~~Genetic toxicology studies have not been performed as biopharmaceuticals, such as mAbs, do not interact directly with DNA or chromosomes~~**The binding of GSK1223249 to the central nervous tissue in rabbit has also been assessed.**

In Vitro Studies

PREVIOUS TEXT

The in vitro flow cytometric cross reactivity study demonstrated no specific binding of GSK1223249 to cynomolgus monkey, human, or rat peripheral blood cells. The in vitro human tissue cross-reactivity study also demonstrated good concordance between the human, monkey and rat in terms of the range of tissues in which GSK1223249 specific staining was observed. Importantly, all tissues/histological structures in which specific staining was observed in humans, were also positive in the rat and monkey, confirming the relevance of these species for the toxicological evaluation of GSK1223249. Specific, consistent binding to cells with morphology and distribution characteristic of central and peripheral nervous system tissues was observed in all three species. In addition, specific staining was observed in muscular structures within a number of tissues. Whether or not this staining reflects tissue innervation or non-specific binding within these tissues remains unclear given the absence of Nogo-A expression outside of the CNS in normal animals. Some inconsistent staining was observed in various other tissues but not always in all three species; this most likely reflects the high concentrations of biotin labelled GSK1223249 that had to be used. Nevertheless, as there was no staining in human tissues that was not observed in animals then this binding is not considered to be of concern given the negative in vivo repeat dose toxicity studies.

REVISED TEXT

The in vitro flow cytometric cross-reactivity study demonstrated no specific binding of GSK1223249 to cynomolgus monkey, human or rat peripheral blood cells. The in vitro~~human~~tissue cross-reactivity ~~study~~**studies** also demonstrated good concordance between ~~the~~ human, monkey and rat in terms of the range of tissues in which GSK1223249 specific staining was observed. Importantly, all tissues/histological structures in which specific staining was observed in humans were also positive in the rat and monkey, confirming the relevance of these species for the toxicological evaluation of GSK1223249. Specific,consistent binding to cells with morphology and distribution characteristic of central and peripheral nervous system tissues was observed in all 3

species **and in a separate study in rabbit as well**. In addition, specific staining was observed in muscular structures within a number of tissues ~~Whether or not this staining reflects tissue innervations or non specific binding within these tissues remains unclear given the absence of Nogo A expression outside the CNS in normal animals.~~ **in rat, monkey and human.** Some ~~inconsistent~~**inconsistencies in** staining ~~was~~**were** observed in ~~various~~ **a few tissues in one or the** other, ~~but not always in all three~~**animal** species~~, this~~ **and were** most likely ~~reflects the~~ **due to** high concentrations of ~~biotin labelled~~ **biotinylated** GSK1223249 ~~that had to be~~ used. ~~Nevertheless, as there was no staining in human tissues that was not observed in anaimals then this binding~~**This** is not considered to be of concern given the ~~negative~~**lack of adverse findings in the** in vivo repeat dose toxicity studies **and that there was no unique staining observed in human tissue that was not observed in animals.**

In Vivo Studies

PREVIOUS TEXT

An assessment of cardiovascular and respiratory safety pharmacology in the cynomolgus monkey showed no acute GSK1223249-related effects on ventilatory function, airway resistance, arterial blood pressures, ECG intervals, or any evidence of waveform abnormalities or arrhythmias following IV (bolus) administration at doses of 30 or 300 mg/kg. Mild, transient, decreases in mean heart rate (up to 12% from 1 to 3 hours post dose) were observed in the low dose treatment group (30 mg/kg) compared with the vehicle control group. Mild, decreases in mean heart rate of a similar degree (up to 17%) were also observed in the high dose treatment group (300 mg/kg) and persisted for 7 days. The decreases in heart rate are considered mild since there was no statistically significant differences ($p>0.05$) between the mean heart rate in either treatment group compared with vehicle control, at any time point, and the absolute heart rate values of the treatment groups remained within the range of absolute heart rate values for the vehicle control animals throughout the observation period. Moreover, these effects were not associated with any changes in systolic, diastolic or pulse blood pressures. A dose of 300 mg/kg also produced a mild and transient increase (0.55°C) in body temperature, which was not observed in the repeat dose study.

GSK1223249 was administered as an IV (bolus) injection to male and female rats and cynomolgus monkeys at doses of 30, 100 and 300 mg/kg once every 2 weeks for 12 weeks. There was no toxicity or irritancy associated with IV administration of GSK1223249 and the mAb was well tolerated in both species. One male rat in the low dose group (30 mg/kg) was found dead on Day 83. As no study drug related or significant macroscopic or microscopic changes were noted, this death was not considered attributable study drug administration. Otherwise, there were no significant clinical observations or toxicities observed based on assessments of body weight and food consumption, body temperature, ophthalmology, neurobehavioral evaluations and electrocardiography (cynomolgus monkey only), clinical pathology, organ weights, macroscopic and microscopic observations. There was no evidence of any toxicity on the male and female reproductive organs in either species (including stage dependent evaluation of spermatogenesis). In particular, there were no adverse cardiovascular effects or significant changes in body temperature in the monkey. There were no anti-

GSK1223249 antibodies present in either species and there was no evidence for delayed toxicities following an 8 week treatment-free period after the final dose of GSK1223249 (high dose groups only).

Mice with varying degrees of targeted gene disruption (gene knockout) of Nogo isoforms have been analysed and published independently by three research groups: Nogo-A/B$^{(-/-)}$ mice [Kim, 2003] Nogo-A$^{(-/-)}$ mice [Simonen, 2003], and Nogo-A/B$^{(-/-)}$ or Nogo-A/B/C$^{(-/-)}$ mice [Pernet, 2008; Zheng, 2003]. None of the Nogo-A knockout mice showed any major developmental or neurological disturbances and all are viable and fertile (with expected Mendelian frequency), grow with normal body weights and display no obvious behavioural alterations (assessed by primary neurological screening) compared with wild type animals. They have no major differences in gross brain anatomy or development of the main fiber tracts, such as the optic nerve. In other words, targeted disruption of Nogo does not appear to cause any major adverse effects. The fact that brain structure and function is normal in Nogo-A knockout mice provides a degree of reassurance that Nogo-A blockade with anti-Nogo-A antibodies, such as GSK1223249, is unlikely to result in adverse developmental, neurological or behavioural disturbances. Furthermore, in the definitive repeat-dose monkey toxicity study there were no adverse effects in the extensive neurobehavioral functional assessments, reflecting normal peripheral and central nervous system activity. This was supported by the absence of abnormal pathology in a wide-ranging and in-depth assessment of peripheral and central nervous systems.

REVISED TEXT

An assessment of cardiovascular and respiratory safety pharmacology in the cynomolgus monkey showed no ~~acute GSK1223249 related~~effects on ventilatory function, airway resistance, arterial blood pressures, ECG intervals, or any evidence of waveform abnormalities or arrhythmias following **single** IV (bolus) ~~administration at doses~~**dosing** of **GSK1223249 (**30 or 300 mg/kg**).** Mild~~, transient,~~ decreases in mean heart rate (up to 12% from 1 to 3 hours post dose) were observed in the ~~low dose~~ 3**0 mg/kg** treatment group ~~(30mg/kg) compared with the vechile control group. Mild, decrease.~~. **Decreases** in mean heart rate of a similar degree (up to 17%) were also observed in the ~~high dose~~ **300 mg/kg** treatment group ~~(300mg/kg)~~and persisted for 7 days. The decreases in heart rate are considered mild since there was no **difference** between the mean heart rate in either treatment group compared with vehicle control, at any timepoint, and the absolute heart rate values of the treatment groups remained within the range of absolute heart rate values for the vehicle control animals throughout the observation period. Moreover, these effects were not associated with any changes in systolic, diastolic or pulse blood pressures. A dose of 300 mg/kg also produced a mild and transient increase (0.55°C) in body temperature, which was not observed in the repeat dose ~~study~~**studies**.

In repeat dose toxicity studies, GSK1223249 was administered as an IV (bolus) injection to male and female rats and ~~cynomolgus~~monkeys at doses of 30, 100 and 300 mg/kg once every2 weeks for 12 weeks**, or 1000 mg/kg once every 2 weeks for 4 weeks (i.e., maximum feasible dose in the rat and limit dose in the monkey) or 20, 100 and 500 mg/kg once every 2 weeks for 52 weeks (monkey only).** There was no toxicity or irritancy associated with IV administration of GSK1223249 and the mAb was well

tolerated ~~in both species,~~ **in all studies**. One male rat in the low dose group (30 mg/kg) **of the 12 week study** was found dead on Day 83. As no ~~study drug~~**test article-**related or significant macroscopic or microscopic changes were noted, this death was not considered attributable ~~study drug~~**to test article** administration. ~~Otherwise, there~~**There** were no significant clinical observations or toxicities observed **in any repeat dose toxicity study** based on assessments of body weight and food consumption, body temperature, ophthalmology, neurobehavioral evaluations ~~and~~**(cynomolgus monkey 12 week and 52 week studies only),** electrocardiography(cynomolgus monkey only), clinical pathology, organ weights, macroscopic andmicroscopic observations. There was no evidence of any toxicity on the male and female reproductive organs in either species (including stage-dependent evaluation of spermatogenesis **in males).** In particular, there were no adverse cardiovascular effects or significant changes in body temperature in the monkey. There ~~were~~**was** no ~~anti GSK1223249 antibodies~~**evidence of immunogenicity** present in either species ~~and there was no evidence for delayed toxicities~~following ~~an 8 week~~treatment ~~free period after the final period after the final dose of GSK1223249 (high dose groups only)~~ **up to 12 weeks.**

~~Mice with varying degrees of targeted gene disruption (gene knockout) of Nogo isoforms~~ **Development of anti-GSK1223249 antibodies in one animal at 100 mg/kg/dose did not** have ~~been analysed and published independently by three research groups: Nogo-A/B$^{(-/-)}$ mice [Kim, 2003] Nogo-A$^{(-/-)}$ mice [Simonen, 2003], and Nogo-A/B$^{(-/-)}$ or Nogo-A/B/C$^{(-/-)}$ mice [Pernet, 2008, Zheng, 2003]. None of the Nogo-A knockout mice showed any major developmental or neurological disturbances and all are viable and fertile (with expected Mendelian frequency), grow with normal body weights and display no obvious behavioural alterations (assessed by primary neurological screening) compared with wild type animals. They have no major differences in gross brain anatomy or development of the main fiber tracts, such~~ an **impact on study data interpretation because exposure values (AUC) in that animal were within the range of exposure values seen in** the ~~optic nerve~~ other ~~words, targeted disruption~~**animals, at all sampling times, both prior to and after anti-drug antibodies were detected.**

GSK1223249 neutralises and/or antagonises Nogo-A protein which mediates inhibition of neurite outgrowth. Neutralisation of Nogo ~~does not appear~~-**A has been shown** to ~~cause any major~~**promote regeneration in peripheral and central nerves following injury. However, the effect of GSK1223249 on injured or non-injured axons in healthy animals is unknown. Targeted disruption (gene knockout) of Nogo-A, however, in 3 independent mouse models has shown no** adverse **toxicological** effects~~. The fact that,~~**, and additionally in these knockout models,** brain structure and function is normal ~~in Nogo-A knockout mice provides a dgree of reassurance that Nogo-A~~**[Kim, 2003; Simonen, 2003; Zheng, 2003]. Therefore, Nogo-A** blockade with anti-Nogo-A antibodies, such as GSK1223249, is unlikely to result in adverse developmental, neurological or behavioural disturbances. Furthermore, in the ~~definitive~~**12 and 52 week** repeat dose monkey toxicity ~~study~~**studies,** there were no adverse effects in the extensive neurobehavioral functional assessments, reflecting normal peripheral and central nervous system activity. This was supported by the absence of abnormal pathology in ~~a~~ wide ranging and in-depth ~~assessments~~**assessments** of **the** peripheral and central nervous systems.

Biopharmaceutical therapeutics, such as mAbs, do not interact directly with DNA or chromosomes. Therefore, genetic toxicology studies were not performed with GSK1223249.

Reproductive toxicology studies were performed with GSK1223249. In a study to evaluate the effects on female fertility and early embryonic development to implantation, female rats were intravenously administered GSK1223249 at 30, 100 or 500 mg/kg/day as a total of 5 consecutive once weekly doses administered on Days 1, 8 and 15 of the pre-mating treatment phase and on Days 6 and 13 post coitum (pc) during the gestation phase. No maternal or developmental toxicity was detected at any dose; therefore, the no observed adverse effect level (NOAEL) was 500 mg/kg/week. The effects of GSK1223249 on pregnancy and embryofetal development were also investigated in female rabbits as Nogo-A is conserved in this species and expressed in the CNS. Following once weekly IV administration at doses of 30, 100 and 500 mg/kg/week (n=22 mated females/group) on Days 7 and 14 pc, systemic exposures (AUC and C_{max}) increased in a dose-proportional manner in the rabbit and were similar between Days 7 and 14 pc (see Section 3.3.1.2). All dose levels were well tolerated and there were no test article-related deaths or clinical signs and there were no effects on maternal body weight gain or food consumption at any dose. There were no test article effects on pregnancy or fetal developmental parameters. Once weekly dosing resulted in sustained systemic exposure to GSK1223249 across the major period of organogenesis. A proportion of rabbits developed anti-GSK1223249 antibodies during treatment (3 of 22 and 2 of 22 in the 30 or 100 mg/kg/week groups, respectively) but were judged not to have compromised the study because the numbers of animals with no detectable anti-GSK1223249 antibodies or altered pharmacokinetics in each group were sufficient to meet all study objectives. The NOAEL for rabbit embryofetal development was 500 mg/kg/week.

Summary

PREVIOUS TEXT

In conclusion, in the 12 week toxicity studies in rats and monkeys, the no observed adverse effect level (NOAEL) was 300 mg/kg/once every 2 weeks. The mean systemic exposure (male and females combined) of GSK1223249 at 300 mg/kg on Day 1 was AUC_{0-t} of 333 (monkey) and 592 (rat) mg.h/mL, respectively; and on Day 71 was AUC_{0-t} of 425 (monkey) and 1065 (rat) mg.h/mL, respectively. There was no evidence of significant neurobehavioral, cardiovascular or respiratory adverse effects following intravenous administration of GSK1223249 in monkeys and therefore, there are no safety pharmacology findings of concern for clinical development.

REVISED TEXT

In conclusion, **there were no adverse findings** seen in ~~the~~ **4 week,** 12 week **(rats and monkeys) or 52 week (monkey only) toxicity studies**~~ in rats and monkeys, the no observed effect level (NOAEL) was 300mg.kg/~~**following administration of 2 doses**

(given once every 2 weeks) at **1000** mg/kg**/dose (i.e., maximum feasible dose in the rat and limit dose in the** monkey) and 6 **doses (given once every 2 weeks) up to 300 mg/kg/dose, and 26 doses (given once every 2 weeks) up to 500 mg/kg of GSK1223249,** respectively. **Additionally, there** was no evidence of significant neurobehavioral, cardiovascular or respiratory adverse effects following intravenous administration of GSK1223249 in monkeys**. In the 4 week toxicity studies, the no observed adverse effect level (NOAEL) of 1000 mg/kg once every 2 weeks represented a mean AUC_{0-t} of 1550 and 1210 mg.h/mL in rats** and ~~therefore~~**monkeys, respectively, on Day 1 to Day 15; and 1730 and 1380 mg.h/mL in rats and monkeys, respectively, on Day 15 to Day 29. In the 12 week toxicity studies, the NOAEL of 300 mg/kg once every 2 weeks represented a mean AUC_{0-t} of 592 and 333 mg.h/mL in rats and monkeys, respectively, on Day 1; and 1065 and 405 mg.h/mL in rats and monkeys, respectively, on Day 71. In the 52 week toxicity studies, the NOAEL of 500 mg/kg once every 2 weeks represented a mean AUC_{0-t} of 889.5 and 947 mg.h/mL in monkeys in Weeks 1 and 51, respectively. Therefore**, there are no ~~safety pharmacology findings of concern for clinical development~~**findings from the repeat dose toxicology studies that would preclude single or repeat dosing of GSK1223249 in humans.**

Table 2

PREVIOUS TEXT

Table 2 Mean Plasma Toxicokinetic Parameters For GSK1223249 Following Once Every 2 Week Intravenous Administration (bolus) of GSK1223249A in the Rat and Cynomolgus Monkey (Male and Female)

Species (No. of Doses)	Dose (mg/kg)	Sex	C_{max} (mg/mL)			AUC_{0-t} (mg.h/mL)		
			Day 1	Day 43	Day 71	Day 1	Day 43	Day 71[b]
Rat (6 doses)[a]	30 [c]	M<br>F	0.932<br>0.885	1.050<br>0.986	1.310<br>1.260	76.4<br>73.5	112<br>105	111<br>108
	100 [c]	M<br>F	3.53<br>2.71	3.80<br>3.64	4.46<br>4.26	255<br>206	379<br>344	457<br>416
	**300 [d] (NOAEL)**	M<br>F	8.30<br>7.27	11.40<br>11.10	13.30<br>11.70	602<br>582	1050<br>849	1120<br>1010
Species (No. of Doses)	Dose (mg/kg)	Sex	C_{max} (mg/mL)			AUC_{0-t} (mg.h/mL)		
			Day 1	Day 43	Day 71	Day 1	Day 43	Day 71
Monkey (6 doses)[a]	30 [c]	M<br>F	0.740<br>0.874	0.952<br>0.869	0.848<br>0.925	26.2<br>29.1	29.0<br>28.1	27.6<br>28.2
	100 [c]	M<br>F	2.21<br>2.70	2.49<br>3.21	2.65<br>2.88	108<br>88.9	115<br>101	123<br>97.8
	**300 [d] (NOAEL)**	M<br>F	8.45<br>7.29	9.01<br>9.61	10.1<br>9.55	377<br>289	433<br>336	488<br>363

a. **Key: Bolded** doses are the NOAEL (no observed adverse effect level).
b. Doses were administered once every 2 weeks for 12 weeks
c. To enable comparison with Day 1 and 43 AUC0-t data, the AUC0-t for Day 71 was calculated using t = 336 hours
d. n=3/sex
e. n=6/sex

REVISED TEXT

Table 2 Mean Plasma Toxicokinetic Parameters for GSK1223249 Following Once Every 2 Week IV (Bolus) Administration in Male and Female Rats, Rabbits and Monkeys

Species (No. of Doses)	Dose (mg/kg)	Sex	C_{max} (mg/mL)		AUC_{0-t} (mg.h/mL)	
			Days 1 to 15	Days 15 to 29	Days 1 to 15	Days 15 to 29
Rat (2 doses)[a]	1000 (NOAEL)	M<br>F	30.6<br>28.4	32.9<br>32.5	1570<br>1530	1710<br>1750
Monkey (2 doses)[a]	1000 (NOAEL)	M<br>F	30.1<br>25.5	30.4<br>26.3	122<br>1200	1480<br>1280

Species (No. of Doses)	Dose (mg/kg)	Sex	C_{max} (mg/mL)		AUC_{0-t} (mg.h/mL)[f]	
			Day 7	Day 14	Day 7	Day 14
Rabbit (2 doses)[e]	30	F	1.03	1.08	85.2	90.5
	100	F	4.74	5.61	287	283
	500 (NOAEL)	F	20.3	23.0	1236	1173

Species (No. of Doses)	Dose (mg/kg)	Sex	C_{max} (mg/mL)			AUC_{0-t} (mg.h/mL)		
			Day 1	Day 43	Day 71	Day 1	Day 43	Day 71[b]
Rat (6 doses)[a]	30[c]	M<br>F	0.932<br>0.885	1.050<br>0.986	1.310<br>1.260	76.4<br>73.5	112<br>105	111<br>108
	100[c]	M<br>F	3.53<br>2.71	3.80<br>3.64	4.46<br>4.26	255<br>206	379<br>344	457<br>416
	300[d] (NOAEL)	M<br>F	8.30<br>7.27	11.40<br>11.10	13.30<br>11.70	602<br>582	1050<br>849	1120<br>1010
Monkey (6 doses)[a]	30[c]	M<br>F	0.740<br>0.874	0.952<br>0.869	0.848<br>0.925	26.2<br>29.1	29.0<br>28.1	27.6<br>28.2
	100[c]	M<br>F	2.21<br>2.70	2.49<br>3.21	2.65<br>2.88	108<br>88.9	115<br>101	123<br>97.8
	300[d] (NOAEL)	M<br>F	8.45<br>7.29	9.01<br>9.61	10.1<br>9.55	377<br>289	433<br>336	448<br>363

Table 2 (Continued). Mean Plasma Toxicokinetic Parameters for GSK1223249 Following Once Every 2 Week IV (Bolus) Administration in Male and Female Rats, Rabbits and Monkeys

Species (No. of Doses)	Dose (mg/kg)	Sex	C_{max} (mg/mL)				AUC_{0-t} (mg.h/mL)			
			Week 1	Week 13	Week 27	Week 51	Week 1	Week 13	Week 27	Week 51
Monkey (26 doses)[a]	20	M<br>F	0.571<br>0.550	0.528<br>0.588	0.631<br>0.648	0.639<br>0.560	25.0<br>18.7	32.8<br>23.6	40.6<br>30.1	42.7<br>26.1
	100	M<br>F	3.11<br>3.30	3.90<br>3.67	2.76<br>2.61	3.01<br>3.28	119<br>109	197<br>194	152<br>124	164<br>165
	500	M<br>F	19.3<br>20.2	21.3<br>18.1	14.2<br>15.6	14.5<br>16.4	873<br>906	1146<br>1065	923<br>858	934<br>960

Key: **Bolded** doses are the NOAEL (no observed adverse effect level).
a = Doses were administered once every 2 weeks for 4, 12 or 52 weeks (2, 6 or 26 doses in total).
b = To enable comparison with Day 1 and 43 AUC_{0-t} data, the AUC_{0-t} for Day 71 was calculated using t = 336 hours.
c = n=3/sex.
d = n=6/sex.
e = Once weekly IV administration of GSK1223249 via ear vessel on Days 7 and 14 post coitum
f = AUC_{0-t} was calculated using t = 168 hours.
NOAEL results are reported as mean and [range].

Section 1.2.4 Pre-Clinical Pharmacokinetics and Product Metabolism

PREVIOUS TEXT

1.2.4 Pre-Clinical Pharamcokinetics

The Pharmacokinetic parameters of GSK1223249 were determined in rat and cynomolgus monkey. Following single and repeat (bi-weekly dosing for up to 3 months) intravenous doses of GSK1223249, systemic exposure (AUC and C_{max}) to the antibody increased approximately proportionally with dose, between 30 and 300 mg/kg in both rats and cynomolgus monkeys. The plasma clearance of GSK1223249 is low in both rat (<1 mL/min/kg) and monkey (0.7 mL/h/kg) and is consistent with its observed terminal half life in rat (~180 hrs) and monkey (ranging from 249 to 388 hrs). The volume of distribution in rat (100 mL/kg) and monkey (84 mL/kg) indicates that GSK1223249 is largely confined to the systemic circulation and likely there was little uptake into the tissues. Accumulation following repeat dose in the rat was consistent with the observed terminal half life of GSK1223249. No notable sex differences in exposure were observed.

REVISED TEXT

1.2.4 Pre-Clinical Pharmacokinetics and Product Metabolism

~~The Pharmacokinetic parameters of GSK1223249 were determined in rat and cynomolgus monkey.~~ Following single ~~single and repeat (bi-weekly dosing for up to 3~~

~~months)~~ intravenous ~~doses of GSK1223249, systemic exposure (AUC and C_{max}) to the antibody increased approximately proportionally with dose, between 30 and 300 mg/kg in both rats and cynomolgus monkeys. The plasma clearance~~ **(IV) infusion** of GSK1223249 ~~is low in both rat (<1 mL/min/kg) and monkey (0.7 mL/h/kg) and is consistent with its observed terminal half life in rat (180 hrs) and monkey (ranging from 249 to 388 hrs). The~~ at 1 and **1.5 mg/kg to male Sprague Dawley rats and cynomolgus monkeys, respectively, plasma clearance (CL) and steady state** volume of distribution **(V_{ss}) were low** in ~~rat (100~~**both species (CL = <1** mL/**min/**kg and ~~monkeys (84 mL~~V_{ss} = ~0.1 L/kg ~~indicates~~), **indicating** that GSK1223249 ~~is largely~~**was mainly** confined to systemic circulation~~. and likely there was little uptake into the tissues. Accumulation following repeat dose in the rate was consistent with the observed terminal half life.~~. **The apparent terminal half-life ($t_{½}$) was estimated to be 8 and 4 days (193 and 92.2 hours) in rats and monkeys, respectively. Following single subcutaneous (SC) administration** of GSK1223249 ~~No~~ **at 1 and 1.5 mg/kg to male Sprague Dawley rats and cynomolgus monkeys, respectively, bioavailability was estimated to be 68% (rats) and 100% (monkeys).**

Following repeat IV administration of GSK1223249 at doses of 30, 100 and 300 mg/kg (dosed once every other week for 12 weeks for a total of 6 doses) and 1000 mg/kg (dosed once every other week for 4 weeks for a total of 2 doses) in rats and monkeys, systemic exposure to GSK1223249 (C_{max} and AUC_{0-336}) increased generally dose-proportionally. In both rats and monkeys, there were no notable ~~sex~~**differences in systemic exposure between males and females at any dose or sampling occasion. In rats at 30, 100 and 300 mg/kg, after 6 doses, the systemic exposure was 1.5- and 1.7-fold higher (C_{max} and AUC_{0-336}, respectively) compared to exposure after one dose. In monkeys at 30, 100 and 300 mg/kg, there were no** differences in ~~exposure were observed~~**systemic exposure (C_{max} and AUC_{0-336}) after one dose compared to 6 doses. GSK1223249 was detected in the plasma at the end of the 8 week off-dose recovery period in both rats and monkeys. At 1000 mg/kg, although there were no marked changes in systemic exposure (C_{max} and AUC_{0-336}), there was a trend towards an increase in systemic exposure after the second administration in both rat and monkey.**

Section 1.3.2 Dose Rationale

NEW TEXT

Emerging clinical data

Emerging clinical data from Part 1 of this protocol, in which a total of 40 subjects were dosed at increasing single dose levels including 15mg/kg, suggests that this dose is tolerated well in patients with ALS. Thus, an increase in the top repeat dose in Part 2 to two doses of 15 mg/kg given 28 days apart is deemed to be in line with a safe and cautious approach to fully characterize GSK1223249 and its effects in the ALS patient population in this early phase of clinical development.

The emerging pharmacokinetics data in ALS subjects from Part 1 indicated that GSK1223249 plasma concentrations in ALS subjects were within 2 to 3 fold higher than the predictions based on allometric scaling of monkey data. The PK parameters calculated from these PK observations are as follows: a central compartment volume Vc of ~ 40 mL/kg, a human clearance of ~ 0.14 mL/h/kg and a half life of ~20 days.

Based on these updated PK data from Part 1, the Cmax and AUC values predicted in humans were refined (see Table 3 and Table 4)

Selection of top dose based on safety cover

PREVIOUS TEXT

A NOAEL of GSK1223249 of 300 mg/kg has been determined in 12-week toxicological studies in two species (rat and monkey). Predicted safety margins have been calculated using the AUC and Cmax associated with this NOAEL in monkeys and the predicted human AUC and Cmax of the planned doses – see Table 3. The proposed maximum dose of 15 mg/kg has been selected based on a conservative 20-fold safety margin on a mg/kg basis, 15 fold on the basis of AUC and 60 fold on the basis of Cmax.

REVISED TEXT

~~A NOAEL of GSK1223249 of 300mg/kg has been determined in 12 week toxicological studies in two species (rat and monkey). Predicted safety margins have been calculated using the AUC and Cmax associated with this NOAEL in monkeys and the predicted human AUC and Cmax of the planned doses see~~**GSK1223249 was well tolerated in the cynomolgus monkey following fortnightly (one dose every two weeks) IV bolus administration for 52 weeks, at doses of 20, 100 and 500 mg/kg. The no observed adverse effect level (NOAEL) was 500 mg/kg (the highest dose examined). The systemic exposure (as defined by $AUC_{0\text{-}2\ weeks}$ and C_{max}) was similar between male and female monkeys. The mean systemic exposure (males and females combined) of GSK1223249 at 500 mg/kg for the first dose was $AUC_{0\text{-}2\ weeks}$ of 890 mg.h/mL and C_{max} of 19.8 mg/mL; and for the last dose was $AUC_{0\text{-}\tau}$ of 947 mg.h/mL and C_{max} of 15.4 mg/mL.**

The NOAEL $AUC_{0\text{-}2\ weeks}$ of 890 mg.h/mL (= 890000 µg.h/mL) and C_{max} of 15.4 mg/mL (= 15400 µg/mL) have been conservatively selected for the calculation of safety covers for planned doses of this study, as shown in Table 3~~The proposed maximum dose of 15 mg/kg has been selected based on~~ **and Table 4. Because of differences in frequency in dosing the monkeys, $AUC_{0\text{-}2\ weeks}$ represents** a conservative ~~20 fold~~**estimate over the human cover represented by $AUC_{0\text{-}inf}$ after a single dose (Table 3) and $AUC_{0\text{-}4\ weeks}$ after 2 doses, 4 weeks apart (Table 4).**

Two doses of 15 mg/kg separated by 28 days is the planned maximum dosing regimen that will be explored in this study. The corresponding safety margin on a mg/kg basis, 15**margins are 33-fold based on dose, 10-**fold based on the basis of AUC**$AUC_{0-\tau}$** and 6035-fold **based** on the basis ofC_{max}

Table 3

PREVIOUS TEXT

Table 3 Predicted safety cover of planned humans doses, using a NOAEL in monkeys of 300 mg/kg given bi-weekly for 12 weeks

Proposed clinical dose of GSK1223249	Predicted human exposure based on allometric scaling of male monkey single dose data		Predicted exposure cover in humans		
Dose (mg/kg)	C_{max} (µg/mL)	AUC_{0-inf} (µgxhr/mL)	Based on dose ratio[a]	Based on AUC ratio[b]	Based C_{max} ratio[c]
0.01	0.113	22.2	30000x	22026x	89646x
0.1	1.13	222	3000x	2203x	8965x
1	11.3	2216	300x	220x	896x
5	56	11078	60x	44x	179x
15	169	33233	20x	15x	60x

a.Monkey NOAEL/human dose (mg/kg)
b.AUC0-tau-day-71,monkey / AUC0-inf-Day-1, human (AUC0-tau-Day71, monkey = 488 mg*hr/mL)
c.Cmax-day-71, monkey / Cmax-Day1, human (Cmax-Day71-monkey = 10.1 mg/mL)

REVISED TEXT

Table 3 PredictedSingle Dose Part 1: Estimated safety cover of planned single doses, in humans doses(part 1), using a NOAEL in monkeys of 300500 mg/kg given bi weekly for 12once every 2 weeks for 52 weeks.

Proposed clinical dose of GSK1223249	PredictedEstimated human exposure based on allometric scaling of male monkey single doselast updated clinical data from NOG111330		Predicted exposure cover in humans		
Dose (mg/kg)	C_{max} (µg AUC$_{0\text{-}inf}$ (µgxh/mL)	AUC$_{0\text{-}inf}$ (µgxhr Cmax (µg/mL)	Based on dose ratio[a]	Based on AUC ratio[b]	Based C_{max} ratio[c]
0.01	0.113 71.4	22.2 0.3	30000x 50000 x	22026x 12465 x	89646x 51333 x
0.1	1.13 714.3	222 2.6	3000x 5000 x	2203x 1246 x	8965x 5133 x
1	11.37 142.8	2216 26.3	300x 500 x	220x1 24.6 x	896x 513 x
5	56 35714.3	11078 131.5	60x 100 x	44x 25 x	179x 117 x
15	169 107149.0	33233 394.4	20x 33 x	15x 8 x	60x 39 x

a.Monkey NOAEL / human dose (mg/kg)
b.Monkey NOAEL AUC$_{0\text{-}2weeks}$ / human AUC$_{0\text{-}inf}$
c.Monkey NOAEL Cmax / human Cmax

Table 4

NEW TEXT

Table 4 2nd Dose of the Two Doses Separated by 28 days, Part 2: Predicted safety cover of planned 2nd dose in humans (Part 2), using a NOAEL in monkeys of 500 mg/kg once every 2 weeks for 52 weeks.

Proposed clinical 2nd dose of GSK1223249	Estimated human exposure based on last updated clinical data from NOG111330		Predicted exposure cover in humans		
Dose (mg/kg)	AUC_{0-T} (μgxh/mL)	Cmax (μg/mL)	Based on dose ratio[a]	Based on AUC ratio[b]	Based C_{max} ratio[c]
0.5	3070	14.7	1000 x	290 x	1048 x
2.5	16915	73.3	200 x	53 x	210 x
15	92096	440.1	33 x	10 x	35 x

AUC_{0-T} represent the AUC from 0 to 4 weeks
a.Monkey NOAEL / human 2nd dose (mg/kg)
b.Monkey NOAEL $AUC_{0-2weeks}$ / human $AUC_{0-4weeks}$
c.Monkey NOAEL Cmax/ human Cmax

Section 2 Objectives and Endpoints of the Study

Table Title

PREVIOUS TEXT

Table 4 Objectives and Endpoints of the Study

REVISED TEXT

Table ~~4~~ 5 Objectives and Endpoints of the Study

Section 3.1 Study Design/Study Schematic

PREVIOUS TEXT

Table 6 Schematic of FTIH Design

2-Part Study: Randomised Placebo-Controlled Double-Blind Sequential Dose Escalation Design																			
**Part 1: Single Dose Escalation (N ~ 40). Doses in mg/kg i.v. A = Active, P = Placebo. Open muscle biopsy (◆) before and after treatment in Cohort 3 (1mg/kg), are voluntary collections, but in Cohort 5 (15mg/kg) they are required collections ('low and high dose muscle biopsy cohorts').**																			
Cohort 1 (2P, 6A)	0.01																		
Cohort 2 (2P, 6A)		0.1																	
Cohort 3 (2P, 6A)			◆	1	◆														
Cohort 4 (2P, 6A)						5													
Cohort 5 (2P, 6A)							◆	15	◆										
**Part 2: Repeat Dose Escalation – open muscle biopsy (◆) before and after treatment (n~ 36)**																			
Cohort 6 (3P, 9A)										◆	0.5	0.5	◆						
Cohort 7 (3P, 9A)													◆	2.5	2.5	◆			
Cohort 8 (3P, 9A)																◆	7.5	7.5	◆

REVISED TEXT

Table 6 Schematic of FTIH Design

2-Part Study: Randomised Placebo-Controlled Double-Blind Sequential Repeat Dose Escalation Design																
**Part 1: Single Dose Escalation (N ~ 40). Doses in mg/kg i.v. A = Active, P = Placebo. Open muscle biopsy (◆) before and after treatment in Cohort 3 (1mg/kg), are voluntary collections, but in Cohort 5 (15mg/kg) they are required collections ('low and high dose muscle biopsy cohorts').**																
Cohort 1 (2P, 6A)	0.01															
Cohort 2 (2P, 6A)		0.1														
Cohort 3 (2P, 6A)			◆	1	◆											
Cohort 4 (2P, 6A)						5										
Cohort 5 (2P, 6A)							◆	15	◆							
**Part 2: Repeat Dose Escalation – open muscle biopsy (◆) before and after treatment (n~ 36)**																
Cohort 6 (3P, 9A)							◆	**0.5**	**0.5**	◆						
Cohort 7 (3P, 9A)										◆	2.5	2.5	◆			
Cohort 8 (3P, 9A)													◆	**15**	◆	**15**

Section 3.2 Discussion of Design

PREVIOUS TEXT

Part 2 will also be of a sequential dose escalating design, with subjects in each of the planned 3 cohorts (3 placebo, 9 active in each cohort) receiving 2 repeat i.v. doses separated by approximately 4 weeks. The planned repeat doses in Part 2 are dependent on demonstration of the safety of equivalent or higher doses in Part 1 (see Section 3.3.1 for data required for dose escalation decisions and Section 3.3.4 for criteria for progression from Part 1 to Part 2). In Part 2, safety and PK will be evaluated, and open skeletal muscle biopsies and blood samples will be collected from patients at pre-dose and at week 8 (unless emerging data suggests the post dose muscle biopsy and blood sample collection should be collected at an alternative week) to demonstrate whether or not GSK1223249 binds to its target and produces any measurable pharmacodynamic (PD) effect.

REVISED TEXT

Part 2 will also be of a sequential dose escalating design, with subjects in each of the planned 3 cohorts (3 placebo, 9 active in each cohort) receiving 2 repeat i.v. doses separated by approximately 4 weeks. The planned repeat doses in Part 2 are dependent on demonstration of the safety of equivalent or higher doses in Part 1 (see Section 3.3.1 for data required for dose escalation decisions and Section 3.3.4 for criteria for progression from Part 1 to Part 2). In Part 2, safety and PK will be evaluated.~~, and open skeletal muscle biopsies and blood samples will be collected from patients at pre-dose and at week 8 (unless emerging data suggests the post dose muscle biopsy and blood sample collection should be collected at an alternative week) to demonstrate whether or not GSK1223249 binds to its target and produces any measurable pharmacodynamic (PD) effect.~~

- **In cohorts 6 and 7 open skeletal muscle biopsies and biomarker blood samples will be collected. Muscle biopsies and blood sample will be collected from patients at pre-dose and at post dose Week 8.**
- **In cohort 8 open skeletal muscle biopsies and biomarker blood samples will be collected. Muscle biopsies and blood sample will be collected from patients at the pre-dose visit and at one timepoint after the first dose, either Day 1(+24hr), Day 8 or Week 4 (Day 22-28): subjects will assigned to a timepoint based on subject preference determined at screening (see Section 6.6.1). If the subject has the Day (1+24hr) collection then the pre-dose muscle biopsy and blood sample must be collected at least 8 days before Day 1.**

(All post dose muscle biopsies collections should be as above unless emerging data suggests these should be collected at an alternative week).

The purpose of collecting muscle biopsies and biomarker blood samples is to demonstrate whether or not GSK1223249 binds to its target and produces any measurable pharmacodynamic (PD) effect.

Section 3.4 Replacement of subjects

PREVIOUS TEXT

However, if necessary, in order to better characterize the safety and tolerability or pharmacokinetics of a given dose, the numbers of subjects per cohort may be increased to 4 additional subjects.

REVISED TEXT

However, if necessary, in order to better characterize the safety and tolerability or pharmacokinetics/**pharmacodynamics** of a given dose, the numbers of subjects per cohort may be increased to 4 additional subjects.

Section 3.5 Treatment Assignment

PREVIOUS TEXT

Randomization constitutes enrollment into the study. The randomization schedule will be computer generated using the Randall system by Discovery Biometrics, GSK.

REVISED

The randomization schedule will be computer generated using the Randall system by Discovery Biometrics, GSK. **In addition, subjects in cohort 8 will be assigned to a post-dose timepoint for the muscle biopsy sample based on subject preference determined at screening (see Section 6.6.1).**

Section 3.6 Description of Investigational Product

Table Title

PREVIOUS TEXT

Table 6 Formulation details of GSK1223249 and Placebo

REVISED TEXT

Table ~~6~~7 Formulation details of GSK1223249 and Placebo

Time and Event Tables

Table Titles

PREVIOUS TEXT

Table 7 Time and Events Table for Subjects in Part 1

REVISED TEXT

Table ~~7~~**8** Time and Events Table for Subjects in Part 1

PREVIOUS TEXT

Table 8 Time and Events Table for Subjects in Part 2

REVISED TEXT

Table ~~8~~**9** Time and Events Table for Subjects in Part 2

Table 8 Time and Events Table for Subjects in Part 1 (Continued) Footnote 6

PREVIOUS TEXT

6. Study assessments to follow PK sampling at end of the infusion

REVISED TEXT

6. Study assessments to follow PK sampling at end of the infusion. **(The 1hr PK sample is to be collected directly at the end of the infusion, cohorts 2-8)**

Table 9 Time and Events Table for Subjects in Part 2

PREVIOUS TEXT

Procedure[11]	Screening	Day-1/or Day 1[2]	Week 1 Day 1 (Dose 1) (Assessment window for 2h-24h is ±15mins)												
**Day/hour relative to Dose 1 (Note: Day is the start of each week)**	**Within 28 days of Day 1**	**Pre-dose**	**Dose**	**1h[6]**	**2h**	**3h**	**4h**	**5h**	**6h**	**8h**	**10h**	**12h**	**14h**	**16h**	**24h**
**Deltoid muscle biopsy[9]**	x[9]														
**Blood sample for biomarkers[9]**		x													

Procedure[11]	Week 2	Week 4	Week 5	Week 5 Day 29 (Dose 2) (Assessment window for 2h-6h is ±15mins)							Week 6	Wk 8	Wk 10	Wk 12	Week 16	Early With-drawal	FU[8]
**Day/hour relative to Dose 1 (Note: Day is the start of each week)**	**Day 8**	**Day 22-28[1]**	**Day 28 or Day 29 Pre-dose[2]**	**Dose**	**1h[6]**	**2h**	**3h**	**4h**	**5h**	**6h**	**Day 36-42[1]**	**Day 50-56[1]**	**Day 64-70[1]**	**Day 78-84[1]**	**Day 106-112[1]**	**Before Week 16**	
**Deltoid muscle biopsy[9]**												x					
**Blood sample for biomarkers[9]**		x										x					

6. Study assessments to follow PK sampling at end of infusion.
9. In Part 2, the pre-dose muscle biopsy should only be done when the subject has passed all screening assessments and eligibility reconfirmed. This means the pre-dose biopsy can be done at any appropriate time before Day 1**.** The post dose muscle biopsy and blood collection is scheduled for collection at week 8, (unless emerging data suggests the post dose muscle biopsy and blood sample collection should be collected at alternative week.
10. Pre-dose 12 lead ECGs will be measured in triplicate and the mean taken as the baseline measurement.
11. The precise timing of safety, PD assessments and PK blood sampling may be altered during the course of the study based on emerging data. If the profile indicates that more sampling or assessments are needed, additional timepoints will be added

REVISED TEXT

**Procedure[12]**	**Screening**	**Day-1/or Day 1[2]**	**Week 1 Day 1 (Dose 1) (Assessment window for 2h-24h is ±15mins)**												
**Day/hour relative to Dose 1 (Note: Day is the start of each week)**	**Within 28 days of Day 1**	**Pre-dose**	**Dose**	**1h[6]**	**2h**	**3h**	**4h**	**5h**	**6h**	**8h**	**10h**	**12h**	**14h**	**16h**	**24h**
**Deltoid muscle biopsy[9] (Cohorts 6-7)**	**x[9]**														
**Deltoid muscle biopsy[10] (Cohort 8 only)**	**x**														**x[10]**
**Blood sample for biomarkers**		x													**x**

**Procedure[12]**	**Wk 2**	**Week 4**	**Week 5**	**Week 5 Day 29 (Dose 2) (Assessment window for 2h-6h is ±15mins)**							**Week 6**	**Wk 8**	**Wk 10**	**Wk 12**	**Week 16**	**Early With-drawal**	**FU[8]**
**Day/hour relative to Dose 1 (Note: Day is the start of each week)**	**Day 8**	**Day 22-28[1]**	**Day 28 or Day 29 Pre-dose[2]**	**Dose**	**1h[6]**	**2h**	**3h**	**4h**	**5h**	**6h**	**Day 36-42[1]**	**Day 50-56[1]**	**Day 64-70[1]**	**Day 78-84[1]**	**Day 106-112[1]**	**Before Week 16**	
**Deltoid muscle biopsy[9] (Cohorts 6 -7)**												**x[9]**					
**Deltoid muscle biopsy[10] (Cohort 8 only)**	**x[10]**	**x[10]**															
**Blood sample for biomarkers**	**X**	**x**										**x[9]**					

6. Study assessments to follow PK sampling at end of infusion**. (The 1hr PK sample is to be collected directly at the end of the infusion, cohorts 2-8)**
9. In Part 2, **Cohorts 6 and 7** the pre-dose muscle biopsy **and blood sample** should only be done when the subject has passed all screening assessments and eligibility reconfirmed. This means the pre-dose biopsy **and blood sample** can be done at any appropriate time before Day 1**.** The post dose muscle biopsy and blood ~~collection~~**sample** is scheduled for collection at **Week** 8, (unless emerging data suggests the post dose muscle biopsy and blood sample ~~collection~~should be collected at an alternative week).
10. **In Part 2 Cohort 8, muscle biopsies and blood sample will be collected from subjects at pre-dose and at one timepoint after the first dose, Subjects will be assigned for a post dose muscle biopsy and blood sample collection at either Day 1(+24hr), Day 8 or Week 4 (Day 22-24) based on subject preference determined at screening (see Section 6.6.1). If the subject has the Day 1(+24hr) collection then the pre-dose muscle biopsy and blood sample must be collected at least 8 days before Day 1.**
11. Pre-dose 12 lead ECGs will be measured in triplicate and the mean taken as the baseline measurement.
12. The precise timing of safety, PD assessments and PK blood sampling may be altered during the course of the study based on emerging data. If the profile indicates that more sampling or assessments are needed, additional timepoints will be added

Section 4.1 Number of Subjects

PREVIOUS TEXT

However, if necessary, in order to better characterize the safety and tolerability or pharmacokinetics of a given dose, up to 4 additional subjects may be recruited per cohort.

REVISED TEXT

However, if necessary, in order to better characterize the safety and tolerability or pharmacokinetics/**pharmacodynamics** of a given dose, up to 4 additional subjects may be recruited per cohort.

Section 4.2.1 Inclusion Criteria for Parts 1 and 2

PREVIOUS TEXT

4. If on any medication (including riluzole), these must have been stable within the previous one month. (See also 'Concomitant Medications' – Section 8).

REVISED TEST

4. If on any medication (including riluzole), these must have been stable within ~~the previous one month~~**28 days prior to dosing.** (See also 'Concomitant Medications' – Section 8).

Section 6.6.1 Skeletal Muscle Biopsies and Blood Samples

PREVIOUS TEXT

Blood samples and skeletal muscle biopsy samples will be taken from patients for evaluation of a variety of pharmacodynamic (PD) biomarkers as described in the objectives and endpoint table (Table 4).

REVISED TEXT

~~Blood~~**Biomarker blood** samples and skeletal muscle biopsy samples will be taken from patients for evaluation of a variety of pharmacodynamic (PD) biomarkers as described in the objectives and endpoint table (Table **5**).

NEW TEXT

Subjects having muscle biopsies (as identified in Table 6) will have two muscle biopsies taken: one at pre-dose and one post the first dose; in addition a biomarker blood sample will be drawn.

- **<u>For cohorts 3 and 5: the post-dose timepoint is Week 4 Days 22-28 (see Table 8)</u>**
- **<u>For cohorts 6 and 7: the post-dose timepoint is Week 4 Days 22-28 (see Table 9)</u>**
- **<u>For cohort 8 only, the schedule for the post dose muscle biopsies will comprise of 3 subgroups. Each subgroup (or stratum) will be assigned to a specific timepoint and will include 4 subjects; within each stratum, subjects will be randomized to placebo or active in a 1:3 ratio (stratified randomization). Subjects will be assigned to a subgroup based on subject preference determined at screening (see Table 10)</u>**

Table10 Cohort 8 Muscle Biopsies Randomization Schedule

<u>Cohort 8 (n=12)</u>	<u>n=4</u>	<u>Stratum 1</u>	<u>Day 1 (+ 24hr) after 1st dose</u>
	<u>n=4</u>	<u>Stratum 2</u>	<u>Day 8 after 1st dose</u>
	<u>n=4</u>	<u>Stratum 3</u>	<u>Day 22-28 after 1st dose</u>

Section 8.1 Permitted Medications

PREVIOUS TEXT

Subjects taking riluzole, statins or other prescribed medication prior to screening are permitted to continue receiving that medication; however, no dose changes should be made within the 1 month prior to screening or during the subject’s study participation.

REVISED TEXT

Subjects taking riluzole, statins or other prescribed medication prior to screening are permitted to continue receiving that medication; however, no dose changes should be made within ~~the 1 month~~**<u>28 days</u>** prior to ~~screening~~**<u>dosing</u>** or during the subject’s study participation.

Section 10.2 Blinding and Dose Escalation Committee (DEC)

Table Title

PREVIOUS TEXT

Table 9 Dose Escalation Committee

REVISED TEXT

Table ~~9~~ **<u>11</u>** Dose Escalation Committee

AMENDMENT 3

Where the Amendment Applies

This amendment applies to all countries and sites.

Summary of Amendment Changes with Rationale

Three eligibility criteria are being modified on recommendation of the experts in ALS:

- The inclusion criterion for onset of muscle weakness is being extended to 60 months to allow those patients with slow disease progression to be included in the study.
- The inclusion criterion for Slow Inspiratory Vital Capacity is being changed to allow patients that are NOT compromised by respiratory insufficiency to be included into the study. Therefore those patients who have low SVC below that what is predicted for age and sex can be included into the study at the discretion of the investigator as long as they are clinically NOT respiratory insufficient.
- The inclusion criterion for Body Mass Index is being made discretionary, as it was thought to be unnecessarily restrictive, given some patients will fall below this and that there are no safety or scientific concerns for omitting the criterion. Nevertheless, the baseline BMI will be recorded and databased.

Handedness is now being recorded and databased as the key opinion leaders viewed this data as important to put MMT scores and muscle-biopsy findings into context.

The ethics committee for the site in France have requested that we amend the protocol to ensure that cohorts 7 & 8 (where the doses will be higher than in cohort 4) will not begin until the data from Part 1 have been reviewed.

Clarification of the data to be consolidated from the interim analysis.

List of Specific Changes:

Section 4.2.1 Inclusion Criteria for Parts 1 and 2, Inclusion 2

PREVIOUS TEXT

2. Onset of muscle weakness within 36 months of study entry.

REVISED TEXT

2. Onset of muscle weakness within ~~36~~ **60** months of study entry.

Section 4.2.1 Inclusion Criteria for Parts 1 and 2, Inclusion 3

PREVIOUS TEXT

3. Slow Inspiratory Vital Capacity (SVC) greater than or equal to 70% predicted for age and sex.

REVISED TEXT

4. ~~Slow Inspiratory Vital Capacity (SVC) greater than or equal to 70% predicted for age and sex.~~ **Patients who have low Slow Inspiratory Vital Capacity (SVC) below that what is predicted for age and sex can be included into the study at the discretion of the investigator as long as they are clinically NOT respiratory insufficient.**

Section 4.2.1 Inclusion Criteria for Parts 1 and 2, Inclusion 9

PREVIOUS TEXT

9. Body Mass Index ≥ 19.0 kgm^2.

REVISED TEXT

9. **A** Body Mass Index ~~≥ 19.0 kgm^2.~~**that at the discretion of the investigator is acceptable for inclusion into the study.**

Section 6.5.1 Functional Assessments

PREVIOUS TEXT

Medical Research Council Muscle Strength Scale

Subjects will be required (with the assistance of the investigator or designee) to complete the Manual Muscle Testing Scale as described in the Time and Events Table, Table 8 and Table 9. The subjects muscle strength will be assessed via Manual Muscle Testing (MMT) by trained personnel. The biopsy sample will be taken from the deltoid muscle whose strength should be grade 3 or 4 on the MRC scale. Patients with wasted deltoids (MRC score ≤2) and normal deltoids MRC score 5 shall be excluded from the study. For details of the MRC Manual Muscle Testing and how to score, see the SPM.

REVISED TEXT

Medical Research Council Muscle Strength Scale

Subjects will be required (with the assistance of the investigator or designee) to complete the Manual Muscle Testing Scale as described in the Time and Events Table, Table 8 and Table 9. The subjects muscle strength will be assessed via Manual Muscle Testing (MMT) by trained personnel. **The subject's handedness will be recorded.** The biopsy sample will be taken from the deltoid muscle whose strength should be grade 3 or 4 on the MRC scale. Patients with wasted deltoids (MRC score ≤2) and normal deltoids MRC score 5 shall be excluded from the study. For details of the MRC Manual Muscle Testing and how to score, see the SPM.

Section 3.3.4 Criteria for Progression from Part 1 to Part 2 Paragraph 1

PREVIOUS TEXT

The decision for the study to proceed to Part 2 will be taken by the DEC after the interim analysis at the end of cohort 4. Provided there are no safety concerns cohort 5 in part 1 will run in parallel with cohort 6 in part 2. Cumulative data (focusing on safety and PK) collected from all subjects up to and including the Day 8 Visit will be evaluated blinded and non-Quality Controlled (QC'ed). Considering the time it will take to collect this data and have a thorough evaluation by the DEC, there will be an appropriate period between dosing of the last patient in cohort 4 and dosing of the first patients in cohort 5 and cohort 6. The planned total dose in cohort 6 is dependent on demonstration of the safety of equivalent or higher single doses in Part 1 as shown in Table 5. A blinded summary of the safety data from the single doses administered in Part 1 will be sent to the Chair of the GSK Global Safety Board for information. If required, this blinded data summary may also be sent to appropriate Regulatory Authorities, IRBs or IECs, for their information along with the decision to proceed. However, it is proposed that the study will proceed to Part 2 **without the need for further approval** from such bodies.

REVISED TEXT

The decision for the study to proceed to Part 2 will be taken by the DEC after the interim analysis at the end of cohort 4. Provided there are no safety concerns cohort 5 in part 1 will run in parallel with cohort 6 in part 2. **Focusing on safety and PK, cumulative blinded and non-Quality Controlled (QC'ed) data will be analysed from all subjects up to and including the Day 8 Visit, at a minimum, prior to proceeding between cohorts.** ~~Cumulative data (focusing on safety and PK) collected from all subjects up to and including the Day 8 Visit will be evaluated blinded and non-Quality Controlled (QC'ed).~~ Considering the time it will take to collect this data and have a thorough evaluation by the DEC, there will be an appropriate period between dosing of the last patient in cohort 4 and dosing of the first patients in cohort 5 and cohort 6. The planned total dose in cohort 6 is dependent on demonstration of the safety of equivalent or higher single doses in Part 1 as shown in Table 5. **In addition, cohort 7 will not begin until the data collected from all subjects (as a minimum up to and including the Day 8 Visit) from cohort 5 have also been evaluated.** A blinded summary of the safety data from the single doses administered in **cohorts 1 to 4 of** Part 1 will be sent to the Chair of

the GSK Global Safety Board for information. If required, this blinded data summary may also be sent to appropriate Regulatory Authorities, IRBs or IECs, for their information along with the decision to proceed. However, it is proposed that the study will proceed to Part 2 **without the need for further approval** from such bodies.

Section 5.3.1 Interim Analysis

PREVIOUS TEXT

A formal interim analysis is planned to consolidate all safety and pharmacokinetic data at the end of cohort 4 from Part 1 of the study.

REVISED TEXT

A **blinded** interim analysis is planned to consolidate ~~all~~**the** safety and pharmacokinetic data ~~at the end of~~ **for** cohort**s** **1 to** 4 from Part 1 of the study.

AMENDMENT 2

Where the Amendment Applies

This amendment applies to all countries and sites.

Summary of Amendment Changes with Rationale

Inclusion of IND number which was not available at for the previous version of the protocol.

There are currently 2 cohorts (3 and 5) in part I of the protocol which require biopsies. Updated feasibility assessments suggest that especially in the lower dose cohort 3, in which patients will be dosed with 1mg/kg of GSK1223249, patients are reluctant to enter this cohort, from which they will unlikely derive any benefit.

Although biomarker assessments using biopsies are a pivotal part in this trial to determine the pharmacodynamic effect of the IP and facilitate PK-PD modelling, biopsy data from the single-dose cohort 5 and all multiple dose cohorts (6-8) in this protocol are sufficient to allow for such modelling. Therefore the biopsies in cohort 3 of part 1 are now voluntary.

The text specifying the data to be reviewed for escalation to part 2 of the study has been revised to enhance clarity.

Currently, a safety review meeting at the end of cohort 5, which also constitutes the formal interim analysis between Part 1 and 2 of the overall study, is planned. However, as the total doses to be administered in the first two cohorts of part 2 (cohorts 6 and 7) will have already been tested in cohorts 3 and 4 of part 1, the interim analysis and safety review meeting to endorse proceeding to part 2 is now rescheduled to the end of cohort 4. Provided there are no safety concerns, cohort 5 will then recruit in parallel to cohort 6. A regular dose-escalation meeting will then be held following cohort 6 as well as cumulative safety data review from cohorts 1 - 6 to determine further progression into cohort 7. Dosing in cohort 7 will not start until the data has been evaluated by the dose escalation committee.

The post dose biopsy at week 8 in part 2 may not be the optimal time for collection. Emerging data from this study might suggest that an alternative week would be more suitable, hence the timing of this muscle biopsy has been made more flexible.

The post dose window for some visits has been extended to allow flexibility around subjects returning for assessments.

A pharmacodynamic interaction between a vaccine, such as the influenza-vaccine with the GSK anti-Nogo specific mAb is highly unlikely given their very different modes of action. Hence, changing the time window defining the interval between administration of a vaccine and the GSK1223249 mAb does not alter the benefit:risk ratio for patients enrolling in the NOG111330 trial. The time period between the administration of the vaccine and GSK1223249 can be based on evidence and practical consideration of being able to distinguish between adverse events following a vaccination and those following administration of this investigational product; therefore the minimum time period

between the dosing of a vaccine and GSK1223249 has been reduced from eight to three weeks, adding an additional week to the recommended minimum of two weeks for a more conservative safety cover in a population affected by a severe disease, such as ALS in this instance.

Modification of exclusion criterion 14 to be clear about which subjects the muscle biopsy applies to.

Removal of a sentence relating to full disclosures of ECG, which is not relevant for this study.

Additional text to the Liver Chemistry Testing Procedure in the light of new GSK standards.

Jens Wurthner was added as an author.

List of Specific Changes

Regulatory Agency Identifying Number(s):

PREVIOUS TEXT

IND No. To be confirmed.

REVISED TEXT

IND No. **102832**

Section 3.1 Study Design/Schematic, Table 5 Schematic of FTIH Design

PREVIOUS TEXT

2-Part Study: Randomized Placebo-Controlled Double-Blind Sequential Dose Escalation Design
Part 1: Single Dose Escalation (N ~ 40). Doses in mg/kg i.v. A = Active, P = Placebo.<br>Open muscle biopsy (◆) before and after treatment with 1 and 15 mg/kg ('low and high dose m. biopsy cohorts').

REVISED TEXT

2-Part Study: Randomized Placebo-Controlled Double-Blind Sequential Dose Escalation Design
Part 1: Single Dose Escalation (N ~ 40). Doses in mg/kg i.v. A = Active, P = Placebo.<br>Open muscle biopsy (◆) before and after treatment ~~with~~ **in Cohort 3 (1mg/kg) are voluntary collections but in Cohort 5 (15mg/kg) they are required collections** ('low and high dose m**uscle** biopsy cohorts').

Section 3.2 Discussion of Design Paragraph 3

PREVIOUS TEXT

In Part 1, single escalating intravenous (i.v.) doses of GSK1223249 will be evaluated in sequential cohorts consisting of 2 placebo and 6 active treated subjects per cohort to determine single dose safety and pharmacokinetics (PK). In addition, open skeletal muscle biopsies and blood samples will be collected from patients (in the cohorts receiving 1 and 15 mg/kg only) at pre-dose and at week 4 to demonstrate whether or not GSK1223249 binds to its target and produces any measurable pharmacodynamic (PD) effect.

REVISED TEXT

In Part 1, single escalating intravenous (i.v.) doses of GSK1223249 will be evaluated in sequential cohorts consisting of 2 placebo and 6 active treated subjects per cohort to determine single dose safety and pharmacokinetics (PK). In addition, open skeletal muscle biopsies, ~~and blood samples~~ **which are voluntary collections in Cohort 3 (1mg/kg) and required collections in Cohort 5 (15mg/kg)** ~~will~~ **are to** be collected from patients ~~(in the cohorts receiving 1 and 15 mg/kg only)~~ at pre-dose and at week 4 to demonstrate whether or not GSK1223249 binds to its target and produces any measurable pharmacodynamic (PD) effect. Blood samples at pre-dose and at week 4 will be collected regardless of whether or not a muscle biopsy will be taken.

Section 3.2 Discussion of Design Paragraph 6

PREVIOUS TEXT

In Part 2, safety and PK will be evaluated, and open skeletal muscle biopsies and blood samples will be collected from patients at pre-dose and at week 8 to demonstrate whether or not GSK1223249 binds to its target and produces any measurable pharmacodynamic (PD) effect.

REVISED TEXT

In Part 2, safety and PK will be evaluated, and open skeletal muscle biopsies and blood samples will be collected from patients at pre-dose and at week 8, **(unless emerging data suggests the post dose muscle biopsy and blood sample collection should be collected at an alternative week)** to demonstrate whether or not GSK1223249 binds to its target and produces any measurable pharmacodynamic (PD) effect.

Section 3.3.1 Planned Doses and Data Required for Dose Escalation Paragraph 2 and 3

PREVIOUS TEXT

Based on careful evaluation of all emerging cumulative data (safety, PK and other data), escalation may be to a lower dose than planned, but escalation will not be to a higher dose than planned. The decision to escalate to the next dose will be made by the DEC based on evaluation of all cumulative data (focusing on safety and PK) from the current dose (and preceding doses, where applicable). **In Part 1**, all cumulative data (focusing on safety and PK) collected from all subjects up to and including the Day 8 Visit will be evaluated blinded and non-Quality Controlled (QC'ed) for the dose-escalation decision. Considering the time it will take to collect this data and have a thorough evaluation by the DEC, it is estimated that there will be approximately 4 weeks between dosing of the last patient in the current cohort and dosing of the first patient in the next cohort. **In Part 2**, all cumulative data (focusing on safety and PK) collected from at least six subjects up to and including the Day 36-38 Visit will be evaluated (blinded and non-QC'ed) for the dose-escalation decision.

In both Parts 1 and 2, although all cumulative data will be reviewed, the main data for the dose escalation decision will be the safety and tolerability data. Safety consideration will include any obvious trends in deterioration of Slow inspirational Vital Capacity (SVC), Manual Muscle Testing (MMT), ALSFRS-R and MUNE, where applicable. Escalation will not be stopped based solely on PK grounds alone, unless the predicted AUC for the next dose may exceed the safety cover associated with the NOAEL in primates (see Table 3). PK considerations may thus influence a decision to escalate to a lower dose than planned.

REVISED TEXT

Based on careful evaluation of ~~all~~ emerging cumulative data (safety, PK and other data), escalation may be to a lower dose than planned, but escalation will not be to a higher dose than planned. The decision to escalate to the next dose will be made by the DEC based on evaluation of ~~all~~ cumulative data (focusing on safety and PK) from the current dose (and preceding doses, where applicable). **In Part 1**, ~~all~~ cumulative data (focusing on safety and PK) collected from all subjects up to and including the Day 8 Visit will be evaluated blinded and non-Quality Controlled (QC'ed) for the dose-escalation decision. Considering the time it will take to collect this data and have a thorough evaluation by the DEC, it is estimated that there will be approximately 4 weeks between dosing of the last patient in the current cohort and dosing of the first patient in the next cohort. **In Part 2**, ~~all~~ cumulative data (focusing on safety and PK) collected from at least six subjects up to

and including the Day ~~36-38~~ **36-42** Visit will be evaluated (blinded and non-QC'ed) for the dose-escalation decision.

In both Parts 1 and 2, ~~although all cumulative data will be reviewed,~~ the main data for the dose escalation decision will be cumulative safety and tolerability data from preceding cohorts. Safety consideration will include any obvious trends in deterioration of Slow inspirational Vital Capacity (SVC), Manual Muscle Testing (MMT), ALSFRS-R and MUNE, where applicable. Escalation will not be stopped based solely on PK grounds alone, unless the predicted AUC for the next dose may exceed the safety cover associated with the NOAEL in primates (see Table 3). PK considerations may thus influence a decision to escalate to a lower dose than planned.

Section 3.3.4 Criteria for Progression from Part 1 to Part 2 Paragraph 1

PREVIOUS TEXT

The decision for the study to proceed to Part 2 will be taken by the DEC after the highest dose in Part 1 has been administered. All cumulative data (focusing on safety and PK) collected from all subjects up to and including the Day 8 Visit will be evaluated blinded and non-Quality Controlled (QC'ed). Considering the time it will take to collect this data and have a thorough evaluation by the DEC, there will be an appropriate period between dosing of the last patient in Part 1 and dosing of the first patient in Part 2. The planned repeat doses in Part 2 are dependent on demonstration of the safety of equivalent or higher single doses in Part 1 as shown in Table 5

REVISED TEXT

The decision for the study to proceed to Part 2 will be taken by the DEC after the **interim analysis at the end of cohort 4. Provided there are no safety concerns cohort 5 in part 1 will run in parallel with cohort 6 in part 2.** ~~highest dose in Part 1 has been administered. All c~~**C**umulative data (focusing on safety and PK) collected from all subjects up to and including the Day 8 Visit will be evaluated blinded and non-Quality Controlled (QC'ed). Considering the time it will take to collect this data and have a thorough evaluation by the DEC, there will be an appropriate period between dosing of the last patient in **cohort 4** ~~Part 1~~ and dosing of the first **patients in cohort 5 and cohort 6**~~. Part 2~~. The planned ~~repeat~~ **total dose in cohort 6** ~~Part 2~~ is dependent on demonstration of the safety of equivalent or higher single doses in Part 1 as shown in Table 5

Table 7 Time and Events Table for Subject in Part 1 Visit Windows and Footnotes

PREVIOUS TEXT

Day relative to dose 1 (Note: Day is the start of each week)	Day 8	Day 22-24[1]	Day 36-38[1]	Day 50-52[1]	Day 78-80[1]

1. 1. Visit window is 3 days. All assessments are to be completed within a 48 hour period
2. 9 Muscle biopsies and blood samples for biomarkers before and after treatment will be taken only in the Cohorts that receive 1 and 15 mg/kg or placebo in this part of the study. NOTE: The pre-dose muscle biopsy should only be done when the subject has passed all screening assessments and eligibility reconfirmed. This means the pre-dose biopsy can be done at any appropriate time before Day 1.

REVISED TEXT

Day relative to dose 1 (Note: Day is the start of each week)	Day 8	Day 22-~~24~~**28**[1]	Day 36-~~38~~**42**[1]	Day 50-~~52~~**56**[1]	Day 78-~~80~~**84**[1]

1. 1. Visit window is ~~3~~ **7**days. All assessments are to be completed within a 48 hour period
2. 9 Muscle biopsies ~~and blood samples~~, **which are voluntary collections in cohort 3 (1mg/kg) and required collections in cohort 5 (15mg/kg) are to be collected pre-dose and at week 4**. ~~for biomarkers before and after treatment will be taken only in the Cohorts that receive 1 and 15 mg/kg or placebo in this part of the study. NOTE:~~ The pre-dose muscle biopsy should only be done when the subject has passed all screening assessments and eligibility reconfirmed. This means the pre-dose biopsy can be done at any appropriate time before Day 1. **Blood samples at pre-dose and at week 4 will be collected regardless of whether or not a muscle biopsy will be taken.**

Table 8 Time and Events Table for Subject in Part 2 Visit Windows and Footnotes

PREVIOUS TEXT

Day/hour relative to Dose 1 (Note: Day is the start of each week)	Day 8	Day 22-24[1]	Day 28 or Day 29 Pre-dose[2]	Dose	1h[6]	2h	3h	4h	5h	6h	Day 36-38[1]	Day 50-52[1]	Day 64-66[1]	Day 78-80[1]	Day 106-108[1]

1. Visit window is 3 days. All assessments are to be completed within a 48 hour period

9 In Part 2, the pre-dose muscle biopsy should only be done when the subject has passed all screening assessments and eligibility reconfirmed. This means the pre-dose biopsy can be done at any appropriate time before Day 1.

REVISED TEXT

Day/hour relative to Dose 1 (Note: Day is the start of each week)	Day 8	Day 22-~~24~~**28** [1]	Day 28 or Day 29 Pre-dose [2]	Dose	1h [6]	2 h	3 h	4 h	5 h	6 h	Day 36-~~38~~**42** [1]	Day 50-~~52~~**56** [1]	Day 64-~~66~~**70** [1]	Day 78-~~80~~**84** [1]	Day 106-~~108~~**112** [1]

1. 1. Visit window is ~~3~~ 7days. All assessments are to be completed within a 48 hour period
2. 9 In Part 2, the pre-dose muscle biopsy should only be done when the subject has passed all screening assessments and eligibility reconfirmed. This means the pre-dose biopsy can be done at any appropriate time before Day 1**. The post dose muscle biopsy and blood collection is scheduled for collection at week 8, (unless emerging data suggests the post dose muscle biopsy and blood sample collection should be collected at an alternative week.**

Section 4.2.2 Exclusion Criteria for Parts 1 and 2, Exclusion 12

PREVIOUS TEXT

12. Patients who have received any type of vaccination in the last two months before study entry.

REVISED TEXT

12. Patients who have received any type of vaccination in the last ~~two months~~ **3 weeks** before study drug administration.~~entry~~.

Section 4.2.2 Exclusion Criteria for Parts 1 and 2, Exclusion 14

PREVIOUS TEXT

14. A subject will not be eligible for inclusion in Part 1 (in the cohorts receiving 1 and 15 mg/kg only) and Part 2 of the study if any of the following criteria apply:
    - Patients with wasted deltoids (MRC score ≤ 2) and patients with normal deltoids (MRC score 5).
    - Patients who cannot achieve normal coagulation in the peri-operative period and those who may otherwise be at higher risk of bleeding complications.

REVISED TEXT

14. ~~A subject~~ **Subjects who will undergo muscle biopsies (cohorts 3-optional 5, 6, 7 and 8** will not be eligible for inclusion ~~in Part 1 (in the cohorts receiving 1 and 15 mg/kg only) and Part 2 of the study~~ if any of the following criteria apply:

- Patients with wasted deltoids (MRC score ≤ 2) and patients with normal deltoids (MRC score 5).
- Patients who cannot achieve normal coagulation in the peri-operative period and those who may otherwise be at higher risk of bleeding complications.

Section 5.3.1 Interim Analysis Paragraph 2

PREVIOUS TEXT

A formal interim analysis is planned to consolidate all safety and pharmacokinetic data from Part 1 of the study.

REVISED TEXT

A formal interim analysis is planned to consolidate all safety and pharmacokinetic data **at the end of cohort 4** from Part 1 of the study.

Section 6.4.2 Electrocardiogram (ECG), Paragraph 3

PREVIOUS TEXT

Continuous Lead II ECG monitoring (using telemetry or a bedside ECG monitor) will also be performed during the study as described in the Time and Events Table, Table 8 and Table 9. Any abnormal findings on continuous ECG monitoring should be confirmed by 12-lead ECG. Full disclosures of the ECG's will be maintained as part of the subject's source documents and will be reviewed in detail.

REVISED TEXT

Continuous Lead II ECG monitoring (using telemetry or a bedside ECG monitor) will also be performed during the study as described in the Time and Events Table, Table 8 and Table 9. Any abnormal findings on continuous ECG monitoring should be confirmed by 12-lead ECG. ~~Full disclosures of the ECG's will be maintained as part of the subject's source documents and will be reviewed in detail.~~

Section 12 LIVER CHEMISTRY TESTING PROCEDURES

NEW TEXT AT THE END OF SECTION 12

The procedures listed below are to be followed if a subject meets the criterion of ALT ≥3xULN and <5xULN and bilirubin <2xULN, but does not exhibit hepatitis symptoms or rash:

- Notify the GSK medical monitor within 24 hours of learning of abnormality to discuss subject safety.
- If the subject can be monitored weekly for 4 weeks, continue investigational product and collect liver chemistries within 1 week. Continue weekly collection of liver chemistries up to 4 weeks. If the ALT remains 3xULN after 4 weeks, continue to monitor the liver chemistries weekly until values resolve, stabilize or return to baseline values. If ALT <3xULN at 4 weeks, continue to monitor liver chemistry twice monthly until values normalize or return to baseline.
- If the subject cannot be monitored weekly for 4 weeks, stop investigational product and collect liver chemistries within 1 week. Attempt to collect subsequent liver chemistries weekly if possible, but at a minimum, collection should be twice per month until values resolve, stabilize or return to within baseline values.

Author(s)

NEW TEXT AT AUTHOR(S) SECTION

Wurthner Jens (DM)

AMENDMENT 1

Where the Amendment Applies

This amendment applies to all sites.

Summary of Amendment Changes with Rationale

This amendment was produced at the request of the French regulatory authority, AFSSAPS, to include the procedure for disseminating serious adverse events to all Investigators and study sites. Additionally, the following information was amended:

- Combined 2 bullet points in Appendix 3 (Events Common to ALS) which have related meaning.
- Added urine alcohol test to Section 6.2.7 to enable sites to have a choice of test.
- Included change of Medical Monitor contact details.
- Implemented other minor changes to clarify wording including pre-dose biopsy sampling.

All changes are incorporated into the body of the main protocol.

List of Specific Changes

3.2 Discussion of Design

PREVIOUS TEXT, PARAGRAPHS 3 AND 6:

In Part 1, single escalating intravenous (i.v.) doses of GSK1223249 will be evaluated in sequential cohorts consisting of 2 placebo and 6 active treated subjects per cohort to determine single dose safety and pharmacokinetics (PK). In addition, open skeletal muscle biopsies and blood samples will be collected from patients (in the cohorts receiving 1 and 15 mg/kg only) at screening and at week 4 to demonstrate whether or not GSK1223249 binds to its target and produces any measurable pharmacodynamic (PD) effect.

Part 2 will also be of a sequential dose escalating design, with subjects in each of the planned 3 cohorts (3 placebo, 9 active in each cohort) receiving 2 repeat i.v. doses separated by approximately 4 weeks. The planned repeat doses in Part 2 are dependent on demonstration of the safety of equivalent or higher doses in Part 1 (see Section 3.3.1 for data required for dose escalation decisions and Section 3.3.4 for criteria for progression from Part 1 to Part 2). In Part 2, safety and PK will be evaluated, and open skeletal muscle biopsies and blood samples will be collected from patients at screening and at week 8 to demonstrate whether or not GSK1223249 binds to its target and produces any measurable pharmacodynamic (PD) effect.

REVISED TEXT:

In Part 1, single escalating intravenous (i.v.) doses of GSK1223249 will be evaluated in sequential cohorts consisting of 2 placebo and 6 active treated subjects per cohort to determine single dose safety and pharmacokinetics (PK). In addition, open skeletal muscle biopsies and blood samples will be collected from patients (in the cohorts receiving 1 and 15 mg/kg only) at ~~screening~~ **pre-dose** and at week 4 to demonstrate whether or not GSK1223249 binds to its target and produces any measurable pharmacodynamic (PD) effect.

Part 2 will also be of a sequential dose escalating design, with subjects in each of the planned 3 cohorts (3 placebo, 9 active in each cohort) receiving 2 repeat i.v. doses separated by approximately 4 weeks. The planned repeat doses in Part 2 are dependent on demonstration of the safety of equivalent or higher doses in Part 1 (see Section 3.3.1 for data required for dose escalation decisions and Section 3.3.4 for criteria for progression from Part 1 to Part 2). In Part 2, safety and PK will be evaluated, and open skeletal muscle biopsies and blood samples will be collected from patients at ~~screening~~ **pre-dose** and at week 8 to demonstrate whether or not GSK1223249 binds to its target and produces any measurable pharmacodynamic (PD) effect.

PREVIOUS

Table 7 Time and Events Table for Subjects in Part 1:

Procedure[11]	Screening	Day-1/or Day1[2]	Week 1 Day 1 (Assessment window for 2h-24h is ±15mins)												
**Day/hour relative to dose 1 (Note: Day is the start of each week)**	**Within 28 days of Day 1**	**Pre-dose**	**Dose**	**1h[6]**	**2h**	**3h**	**4h**	**5h**	**6h**	**8h**	**10h**	**12h**	**14h**	**16h**	**24h**
**Informed Consent**	X														
**Medical History and ALS History**	X														
**Concomitant Medication Review**	X	x													x
**Pregnancy test (females only)[5]**	X	x													
**Urine Drug Screen[5]**	X	x													
**Alcohol Test[5]**	X	x													
**Physical examination**	X	x													x
**Neurological Examination**	X														
**ALSFRS-R**	X														
**Manual Muscle strength Test**	X														
**Electrophysiology (MUNE)**	X														
**Slow Inspiratory Vital Capacity (SVC)**	X														
**Randomization**		x													
**Dosing-Slow IV Infusion**			x												
**Adverse Events[4]**	X	x	←--→												
**Clinical laboratory tests**	X	x[2]													x
**Vital Signs (supine and standing)[3]**	X	x[3]		x	x	x	x		x	x		x			x
**12-Lead ECG (QT and other intervals)**	X	x[10]			x		x			x		x			x
**Continuous ECG Monitoring (Telemetry or Bedside ECG Monitor)[7]**		←--→													
**Deltoid muscle biopsy[9]**	X														
**Blood sample for biomarkers[9]**		x													
**Blood sample for immunogenicity**		x													
**Blood sample for PK**		x		x							x				x
**Blood sample for PGx**		x													

Table 7 Time and Events Table for Subjects in Part 1 (Continued)

Procedure[11]	Week 2	Week 4	Week 6	Week 8	Week 12	Early Withdrawal	FU[8]
Day relative to dose 1 (Note: Day is the start of each week)	Day 8	Day 22-24[1]	Day 36-38[1]	Day 50-52[1]	Day 78-80[1]	Before Week 12	
Informed Consent							
Medical History and ALS History							
Concomitant Medication Review	x	x	x	x	x	x	
Pregnancy test (females only)[5]		x			x	x	
Urine Drug Screen[5]							
Alcohol Test[5]							
Physical examination					x	x	
Neurological Examination							
ALSFRS-R			x		x	x	
Manual Muscle strength Test			x		x	x	
Electrophysiology (MUNE)			x		x	x	
Slow Inspiratory Vital Capacity (SVC)			x		x	x	
Randomization							
Dosing-Slow IV Infusion							
Adverse Events[4]	x	x	x	x	x	x	
Clinical laboratory tests	x	x	x	x	x	x	
Vital Signs (supine and standing)[3]	x	x	x	x	x	x	
12-Lead ECG (QT and other intervals)	x	x	x	x	x	x	
Continuous ECG Monitoring (Telemetry or Bedside ECG Monitor)							
Deltoid muscle biopsy[9]		x					
Blood sample for biomarkers[9]		x					
Blood sample for immunogenicity	x	x		x	x	x	x
Blood sample for PK	x	x	x	x	x	x	x
Blood sample for PGx							

Continued

Table 7 Time and Events Table for Subjects in Part 1 (Continued)

1. Visit window is 3 days. All assessments are to be completed within a 48 hour period
2. It is recommended that patients be admitted into the clinical unit the day before dosing day. This will allow enough time for some pre-dosing assessments. However, if necessary, patients may be admitted into the clinical unit in the morning of the dosing day. Pre-dose clinical laboratory tests will be done at **both** the Central Lab (Quest) and the Local Site Lab. Data from the Local Lab will be used to ensure the patient's clinical labs are still within the study entry range before dosing. Data from the Central Lab will be used for the database and any comparison with post dose data.
3. Pre-dose vital signs will be measured in triplicate and the mean taken as the baseline measurement. Vital signs are to be taken immediately prior to start of infusion.
4. Only SAEs related to study participation will be collected prior to start of IP. Once the IP infusion begins, all AEs and SAEs are collected through the last follow up visit.
5. Only the screening pregnancy test will be a blood test done at the central lab. All other pregnancy tests will be urine pregnancy tests done at the local lab. The drug screen and alcohol test will be performed at the local lab.
6. Study assessments to follow PK sampling at end of the infusion.
7. Continuous Lead II ECG to commence approximately 1hr pre-dose on Day 1 until 24 hrs post dose.
8. The number and schedule of follow-up visits after Week 12 for each subject will vary depending on plasma concentrations of GSK1223249 reaching a low enough level to allow a final blood sample to be assayed for immunogenicity.
9. Muscle biopsies and blood samples for biomarkers before and after treatment will be taken only in the Cohorts that receive 1 and 15 mg/kg or placebo in this part of the study. NOTE: The pre-dose muscle biopsy should only be done when the subject has passed all other screening assesments – this may require a visit on another day.
10. Pre-dose 12 lead ECGs will be measured in triplicate and the mean taken as the baseline measurement.
11. The precise timing of safety, PD assessments and PK blood sampling may be altered during the course of the study based on emerging data. If the profile indicates that more sampling or assessments are needed, additional timepoints will be added.

REVISED

Table 7 Time and Events Table for Subjects in Part 1

Procedure[11]	Screening	Day-1/or Day1[2]	Week 1 Day 1 (Assessment window for 2h-24h is ±15mins)												
Day/hour relative to dose 1 (Note: Day is the start of each week)	Within 28 days of Day 1	Pre-dose	Dose	1h[6]	2h	3h	4h	5h	6h	8h	10h	12h	14h	16h	24h
Informed Consent	X														
Medical History and ALS History	X														
Concomitant Medication Review	X	x													x
Pregnancy test (females only)[5]	X	x													
Urine Drug Screen[5]	X	x													
Alcohol Test[5]	X	x													
Physical examination	X	x													x
Neurological Examination	X														
ALSFRS-R	X														
Manual Muscle strength Test	X														
Electrophysiology (MUNE)	X														
Slow Inspiratory Vital Capacity (SVC)	X														
Randomization		x													
Dosing-Slow IV Infusion			x												
Adverse Events[4]	X	x	←---→												
Clinical laboratory tests	X	x[2]													x
Vital Signs (supine and standing)[3]	X	x[3]		x	x	x	x		x	x		x			x
12-Lead ECG (QT and other intervals)	X	x[10]			x		x			x		x			x
Continuous ECG Monitoring (Telemetry or Bedside ECG Monitor)[7]		←---→													
Deltoid muscle biopsy[9]	X[9]														
Blood sample for biomarkers[9]		x													
Blood sample for immunogenicity		x													
Blood sample for PK		x		x							x				x
Blood sample for PGx		x													

Table 7 Time and Events Table for Subjects in Part 1 (Continued)

Procedure[11]	Week 2	Week 4	Week 6	Week 8	Week 12	Early Withdrawal	FU[8]
Day relative to dose 1 (Note: Day is the start of each week)	Day 8	Day 22-24[1]	Day 36-38 [1]	Day 50-52 [1]	Day 78-80 [1]	Before Week 12	
Informed Consent							
Medical History and ALS History							
Concomitant Medication Review	x	x	x	x	x	x	
Pregnancy test (females only)[5]		x			x	x	
Urine Drug Screen[5]							
Alcohol Test[5]							
Physical examination					x	x	
Neurological Examination							
ALSFRS-R			x		x	x	
Manual Muscle strength Test			x		x	x	
Electrophysiology (MUNE)			x		x	x	
Slow Inspiratory Vital Capacity (SVC)			x		x	x	
Randomization							
Dosing-Slow IV Infusion							
Adverse Events[4]	x	x	x	x	x	x	
Clinical laboratory tests	x	x	x	x	x	x	
Vital Signs (supine and standing)[3]	x	x	x	x	x	x	
12-Lead ECG (QT and other intervals)	x	x	x	x	x	x	
Continuous ECG Monitoring (Telemetry or Bedside ECG Monitor)							
Deltoid muscle biopsy[9]		x					
Blood sample for biomarkers[9]		x					
Blood sample for immunogenicity	x	x		x	x	x	x
Blood sample for PK	x	x	x	x	x	x	x
Blood sample for PGx							

Continued

Table 7 Time and Events Table for Subjects in Part 1 (Continued)

1. Visit window is 3 days. All assessments are to be completed within a 48 hour period
2. It is recommended that patients be admitted into the clinical unit the day before dosing day. This will allow enough time for some pre-dosing assessments. However, if necessary, patients may be admitted into the clinical unit in the morning of the dosing day. Pre-dose clinical laboratory tests will be done at **both** the Central Lab (Quest) and the Local Site Lab. Data from the Local Lab will be used to ensure the patient's clinical labs are still within the study entry range before dosing. Data from the Central Lab will be used for the database and any comparison with post dose data.
3. Pre-dose vital signs will be measured in triplicate and the mean taken as the baseline measurement. Vital signs are to be taken immediately prior to start of infusion.
4. Only SAEs related to study participation will be collected prior to start of IP. Once the IP infusion begins, all AEs and SAEs are collected through the last follow up visit.
5. Only the screening pregnancy test will be a blood test done at the central lab. All other pregnancy tests will be urine pregnancy tests done at the local lab. The drug screen and alcohol test will be performed at the local lab.
6. Study assessments to follow PK sampling at end of the infusion.
7. Continuous Lead II ECG to commence approximately 1hr pre-dose on Day 1 until 24 hrs post dose.
8. The number and schedule of follow-up visits after Week 12 for each subject will vary depending on plasma concentrations of GSK1223249 reaching a low enough level to allow a final blood sample to be assayed for immunogenicity.
9. Muscle biopsies and blood samples for biomarkers before and after treatment will be taken only in the Cohorts that receive 1 and 15 mg/kg or placebo in this part of the study. NOTE: The pre-dose muscle biopsy should only be done when the subject has passed all ~~other~~ screening assessments **and eligibility reconfirmed. This means the pre-dose biopsy can be done at any appropriate time before Day 1.** ~~this may require a visit on another day~~.
10. Pre-dose 12 lead ECGs will be measured in triplicate and the mean taken as the baseline measurement.
11. The precise timing of safety, PD assessments and PK blood sampling may be altered during the course of the study based on emerging data. If the profile indicates that more sampling or assessments are needed, additional timepoints will be added.

PREVIOUS Table 8 Time and Events Table for Subjects in Part 2

Procedure[11]	Screening	Day-1/or Day 1[2]	Week 1 Day 1 (Dose 1) (Assessment window for 2h-24h is ±15mins)												
Day/hour relative to Dose 1 (Note: Day is the start of each week)	Within 28 days of Day 1	Pre-dose	Dose	1h[6]	2h	3h	4h	5h	6h	8h	10h	12h	14h	16h	24h
Informed Consent	X														
Medical History and ALS History	X														
Concomitant Medication Review	X	x													x
Pregnancy test (females only)[5]	X	x													
Urine Drug Screen[5]	X	x													
Alcohol Test[5]	X	x													
Physical examination	X	x													x
Neurological Examination	X														
ALSFRS-R	X														
Manual Muscle strength Test	X														
Electrophysiology (MUNE)	X														
Slow Inspiratory Vital Capacity (SVC)	X														
Randomization		x													
Dosing-Slow IV Infusion			X												
Adverse Events[4]	X	x	←--→												
Clinical laboratory tests	X	x[2]													x
Vital Signs (supine and standing)[3]	X	x[3]		x	x	x	x		x	x		x			x
12-Lead ECG (QT and other intervals)	X	x[10]			x		x			x		x			x
Continuous ECG Monitoring (Telemetry or Bedside ECG Monitor)[7]		←--→													
Deltoid muscle biopsy[9]	X														
Blood sample for biomarkers[9]		x													
Blood sample for immunogenicity		x													
Blood sample for PK		x		x							x				x
Blood sample for PGx		x													

Continued

Table.8 Time and Events Table for Subjects in Part 2 (Continued)

Procedure[11]	Week 2	Week 4	Week 5	Week 5 Day 29 (Dose 2) (Assessment window for 2h-6h is ±15mins)							Week 6	Week 8	Week 10	Week 12	Week 16	Early With-drawal	FU[8]
Day/hour relative to Dose 1 (Note: Day is the start of each week)	Day 8	Day 22-24[1]	Day 28 or Day 29 Pre-dose[2]	Dose	1h[6]	2h	3h	4h	5h	6h	Day 36-38[1]	Day 50-52[1]	Day 64-66[1]	Day 78-80[1]	Day 106-108[1]	Before Week 16	
Informed Consent																	
Medical History and ALS History																	
Concomitant Medication Review	x	x	X								x	x	x	x	x	x	
Pregnancy test (female s only)[5]		x											x		x	x	
Urine Drug Screen[5]			X														
Alcohol Test[5]			X														
Physical examination			X							x					x	x	
Neurological Examination																	
ALSFRS-R		x										x			x	x	
Manual Muscle strength Test		x										x			x	x	
Electrophysiology (MUNE)		x										x			x	x	
Slow Inspiratory Vital Capacity (SVC)		x										x			x	x	
Randomization																	
Dosing-Slow IV Infusion				x													
Adverse Events[4]	x	x	X	←---→							x	x	x	x	x	x	
Clinical laboratory tests	x		x[2]							x	x		x		x	x	
Vital Signs (supine and standing)[3]	x	x	x[3]		x	x	x	x	x	x	x	x	x	x	x	x	
12-Lead ECG (QT and other intervals)	x	x	x[10]			x		x		x	x		x		x	x	

Continued

Table.8 Time and Events Table for Subjects in Part 2 (Continued)

Procedure[11]	Week 2	Week 4	Week 5	Week 5 Day 29 (Dose 2) (Assessment window for 2h-6h is ±15mins)							Week 6	Wk 8	Wk 10	Wk 12	Week 16	Early With-drawal	FU[8]
Day/hour relative to Dose 1 (Note: Day is the start of each week)	Day 8	Day 22-24[1]	Day 28 or Day 29 Pre-dose[2]	Dose	1h[6]	2h	3h	4h	5h	6h	Day 36-38[1]	Day 50-52[1]	Day 64-66[1]	Day 78-80[1]	Day 106-108[1]	Before Week 16	
Continuous ECG Monitoring (Telemetry or Bedside ECG Monitor)[7]			←--→														
Deltoid muscle biopsy[9]												x					
Blood sample for biomarkers[9]		x										x					
Blood sample for immunogenicity	x		X									x	x	x	x	x	x
Blood sample for PK	x	x	X		x					x		x	x	x	x	x	x
Blood sample for PGx																	

1. Visit window is 3 days. All assessments are to be completed within a 48hour period
2. It is recommended that patients be admitted into the clinical unit the day before dosing day. This will allow enough time for some pre-dosing assessments. However, if necessary, patients may be admitted into the clinical unit in the morning of the dosing day. Pre-dose clinical laboratory tests will be done at **both** the Central Lab (Quest) and the Local Site Lab. Data from the Local Lab will be used to ensure the patient's clinical labs are still within the study entry range before dosing. Data from the Central Lab will be used for the database and any comparison with post dose data.
3. Pre-dose vital signs will be measured in triplicate and the mean taken as the baseline measurement.. Vital signs are to be taken immediately prior to start of infusion.
4. Only SAEs related to study participation will be collected prior to start of IP. Once the IP infusion begins, all AEs and SAEs are collected through the last follow up visit.
5. Only the screening pregnancy test will be a blood test done at the central lab. All other pregnancy tests will be urine pregnancy tests done at the local lab. The drug screen and alcohol test will be performed at the local lab.
6. Study assessments to follow PK sampling at end of infusion.
7. Continuous Lead II ECG to commence approximately 1 hr pre-dose until 24 hrs post Dose 1 and for 6 hrs post dose 2.
8. The number and schedule of follow-up visits after Week 16 for each subject will vary depending on plasma concentrations of GSK1223249 reaching a low enough level to allow a final blood sample to be taken for immunogenicity assays.
9. In Part 2, the pre-dose muscle biopsy should only be done when the subject has passed all other screening assesments – this may require a visit on another day.
10. Pre-dose 12 lead ECGs will be measured in triplicate and the mean taken as the baseline measurement.
11. The precise timing of safety, PD assessments and PK blood sampling may be altered during the course of the study based on emerging data. If the profile indicates that more sampling or assessments are needed, additional timepoints will be added

REVISED Table 8 Time and Events Table for Subjects in Part 2

Procedure[11]	Screening	Day-1/or Day 1[2]	Week 1 Day 1 (Dose 1) (Assessment window for 2h-24h is ±15mins)												
Day/hour relative to Dose 1 (Note: Day is the start of each week)	Within 28 days of Day 1	Pre-dose	Dose	1h[6]	2h	3h	4h	5h	6h	8h	10h	12h	14h	16h	24h
Informed Consent	X														
Medical History and ALS History	X														
Concomitant Medication Review	X	x													x
Pregnancy test (females only)[5]	X	x													
Urine Drug Screen[5]	X	x													
Alcohol Test[5]	X	x													
Physical examination	X	x													x
Neurological Examination	X														
ALSFRS-R	X														
Manual Muscle strength Test	X														
Electrophysiology (MUNE)	X														
Slow Inspiratory Vital Capacity (SVC)	X														
Randomization		x													
Dosing-Slow IV Infusion			X												
Adverse Events[4]	X	x	←--→												
Clinical laboratory tests	X	x[2]													x
Vital Signs (supine and standing)[3]	X	x[3]		x	x	x	x		x	x		x			x
12-Lead ECG (QT and other intervals)	X	x[10]			x		x			x		x			x
Continuous ECG Monitoring (Telemetry or Bedside ECG Monitor)[7]		←--→													
Deltoid muscle biopsy[9]	X[9]														
Blood sample for biomarkers[9]		x													
Blood sample for immunogenicity		x													
Blood sample for PK		x		x							x				x
Blood sample for PGx		x													

Continued

Table.8 Time and Events Table for Subjects in Part 2 (Continued)

Procedure[11]	Week 2	Week 4	Week 5	Week 5 Day 29 (Dose 2) (Assessment window for 2h-6h is ±15mins)						Week 6	Week 8	Week 10	Week 12	Week 16	Early With-drawal	FU[8]
Day/hour relative to Dose 1 (Note: Day is the start of each week)	Day 8	Day 22-24[1]	Day 28 or Day 29 Pre-dose[2]	Dose	1h[6]	2h	3h	4h	5h	6h	Day 36-38[1]	Day 50-52[1]	Day 64-66[1]	Day 78-80[1]	Day 106-108[1]	Before Week 16	
Informed Consent																	
Medical History and ALS History																	
Concomitant Medication Review	x	x	X								x	x	x	x	x	x	
Pregnancy test (female s only)[5]		x											x		x	x	
Urine Drug Screen[5]			X														
Alcohol Test[5]			X														
Physical examination			X							x					x	x	
Neurological Examination																	
ALSFRS-R		x										x			x	x	
Manual Muscle strength Test		x										x			x	x	
Electrophysiology (MUNE)		x										x			x	x	
Slow Inspiratory Vital Capacity (SVC)		x										x			x	x	
Randomization																	
Dosing-Slow IV Infusion				x													
Adverse Events[4]	x	x	X	←--→							x	x	x	x	x	x	
Clinical laboratory tests	x		x[2]							x	x		x		x	x	
Vital Signs (supine and standing)[3]	x	x	x[3]		x	x	x	x	x	x	x	x	x	x	x	x	
12-Lead ECG (QT and other intervals)	x	x	x[10]			x		x		x	x		x		x	x	

Continued

Table.8 Time and Events Table for Subjects in Part 2 (Continued)

Procedure[11]	Week 2	Week 4	Week 5	Week 5 Day 29 (Dose 2) (Assessment window for 2h-6h is ±15mins)							Week 6	Wk 8	Wk 10	Wk 12	Week 16	Early With-drawal	FU[8]
Day/hour relative to Dose 1 (Note: Day is the start of each week)	Day 8	Day 22-24[1]	Day 28 or Day 29 Pre-dose[2]	Dose	1h[6]	2h	3h	4h	5h	6h	Day 36-38[1]	Day 50-52[1]	Day 64-66[1]	Day 78-80[1]	Day 106-108[1]	Before Week 16	
Continuous ECG Monitoring (Telemetry or Bedside ECG Monitor)[7]			←--→														
Deltoid muscle biopsy[9]												x					
Blood sample for biomarkers[9]		x										x					
Blood sample for immunogenicity	x		X									x	x	x	x	x	x
Blood sample for PK	x	x	X		x					x		x	x	x	x	x	x
Blood sample for PGx																	

1. Visit window is 3 days. All assessments are to be completed within a 48hour period
2. It is recommended that patients be admitted into the clinical unit the day before dosing day. This will allow enough time for some pre-dosing assessments. However, if necessary, patients may be admitted into the clinical unit in the morning of the dosing day. Pre-dose clinical laboratory tests will be done at **both** the Central Lab (Quest) and the Local Site Lab. Data from the Local Lab will be used to ensure the patient's clinical labs are still within the study entry range before dosing. Data from the Central Lab will be used for the database and any comparison with post dose data.
3. Pre-dose vital signs will be measured in triplicate and the mean taken as the baseline measurement.. Vital signs are to be taken immediately prior to start of infusion.
4. Only SAEs related to study participation will be collected prior to start of IP. Once the IP infusion begins, all AEs and SAEs are collected through the last follow up visit.
5. Only the screening pregnancy test will be a blood test done at the central lab. All other pregnancy tests will be urine pregnancy tests done at the local lab. The drug screen and alcohol test will be performed at the local lab.
6. Study assessments to follow PK sampling at end of infusion.
7. Continuous Lead II ECG to commence approximately 1 hr pre-dose until 24 hrs post Dose 1 and for 6 hrs post dose 2.
8. The number and schedule of follow-up visits after Week 16 for each subject will vary depending on plasma concentrations of GSK1223249 reaching a low enough level to allow a final blood sample to be taken for immunogenicity assays.
9. In Part 2, the pre-dose muscle biopsy should only be done when the subject has passed all ~~other~~ screening assessments ~~– this may require a visit on another day~~ **and eligibility reconfirmed. This means the pre-dose biopsy can be done at any appropriate time before Day 1.**
10. Pre-dose 12 lead ECGs will be measured in triplicate and the mean taken as the baseline measurement.
11. The precise timing of safety, PD assessments and PK blood sampling may be altered during the course of the study based on emerging data. If the profile indicates that more sampling or assessments are needed, additional timepoints will be added

6.2.2 – Neurological Examination

Purpose is to clarify what data is required and where it will be recorded.

- PREVIOUS TEXT:

A complete neurological examination will be performed by medically qualified personnel at those time points as described in the Time and Events Table, Table 8 and Table 9. The examination will include sensory, motor, and autonomic symptoms, pin and vibration sensations, strength and tendon reflexes. All data will be recorded in the eCRF.

REVISED TEXT:

A complete neurological examination will be performed **according to local site protocol** by medically qualified personnel at those time points as described in the Time and Events Tables, Table 8 and Table 9. ~~The examination will include sensory, motor, and autonomic symptoms, pin and vibration sensations, strength and tendon reflexes.~~ ~~All data will be recorded in the eCRF.~~ **All findings are to be documented in the source documents.**

6.2.4 – Electrocardiogram (ECG)

Purpose is to clarify what data is to be recorded. Changes are made to the fourth paragraph, second to last sentence.

PREVIOUS TEXT:

Additionally, PR, QRS, QT, RR and QTc(B) intervals will be recorded in the eCRF.

REVISED TEXT:

Additionally, PR, QRS, QT **and** RR ~~and QTc(B)~~ intervals will be recorded in the eCRF.

6.2.7 Alcohol Test

PREVIOUS TEXT:

An alcohol breath test will be collected and tested at the local lab at screening and on entry to the unit pre-dose (see SPM for details).

REVISED TEXT:

An alcohol **<u>urine or</u>** breath test will be collected and tested at the local lab at screening and on entry to the unit pre-dose (see SPM for details).

11.8 Prompt Reporting of SAEs to GSK

NEW TEXT AFTER PARAGRAPH 3:

The Sponsor's Medical Monitor shall ensure that any SAE report received from one site, will be notified to all participating Investigators of all other active sites immediately and within 24 hours at the latest after being received by GSK. This notification to other Investigators will be made by email and a follow-up telephone call to ensure that they have received and read the email.

Appendix 3: Events common to ALS

PREVIOUS TEXT:

- Airway Secretion/Obstruction
- Mucous

REVISED TEXT:

- **mucous/airway secretion/obstruction**
